# Loneliness and social isolation as risk factors for incident depression in type 2 diabetes mellitus: Evidence from a prospective cohort study

**DOI:** 10.1192/j.eurpsy.2026.12244

**Published:** 2026-07-29

**Authors:** Xin Qi, Li Liu, Chuyu Pan, Wenming Wei, Shiqiang Cheng, Yifan Gou, Yan Wen, Feng Zhang

**Affiliations:** 1 Department of Psychiatry, the First Affiliated Hospital of Xi’an Jiaotong University, China; 2 NHC Key Laboratory of Environment and Endemic Diseases, School of Public Health, Health Science Center, Xi’an Jiaotong University, China; 3 Precision Medicine Center, the First Affiliated Hospital of Xi’an Jiaotong University, China

**Keywords:** depression, loneliness, social isolation, type 2 diabetes mellitus (T2DM)

## Abstract

**Background:**

The role of loneliness and social isolation in the progression from incident type 2 diabetes mellitus (T2DM) to subsequent depression remains underexplored. Further investigation is essential to better understand how these factors influence disease progression.

**Methods:**

This study enrolled 427,594 participants without T2DM and depression at baseline. Multistate models were employed to assess the impact of loneliness and social isolation on transitions from a healthy state to incident T2DM, then to subsequent depression, and finally to death. These associations were further examined across individuals with varying glycemic statuses, including normal glucose tolerance and prediabetes.

**Results:**

Over a median follow-up period of 13.66 years, 14,141 incident cases of T2DM, 1,149 subsequent cases of depression, and 33,024 death events were recorded. Loneliness and social isolation were associated with elevated risks of disease progression across most transitions. For the transition from incident T2DM to subsequent depression, the loneliness group exhibited a significantly higher risk (HR = 1.802, 95% CI: 1.521–2.135) compared to the no-loneliness group. Similarly, the most isolated group had an HR of 1.390 (95% CI: 1.185–1.632) compared to the least isolated group. Additionally, participants with prediabetes faced a higher risk of developing depression following T2DM compared to those with normal glucose tolerance. Interaction analyses revealed that employment status and history of cancer modified the association between loneliness and the transition from baseline to death.

**Conclusion:**

These findings underscore the importance of targeted interventions to address loneliness and social isolation, potentially reducing the risk of disease progression and mortality in individuals with T2DM.

## Introduction

Type 2 diabetes mellitus (T2DM) and depression are increasingly significant global public health issues, exacerbated by rapid socioeconomic development and profound lifestyles changes [1, 2]. Among adults aged 20–79 years, the global prevalence of diabetes was 10.5% (536.6 million people) in 2021, with a projected increase to 12.2% (783.2 million individuals) by 2045 [1]. Furthermore, the worldwide prevalence of prediabetes, characterized by impaired glucose tolerance or impaired fasting glucose, is ~9%, with a projected upward trend [3]. Depression has been the leading cause of years lived with disability since 2010, affecting ~280 million people worldwide [4]. Compared to individuals with normal glucose metabolism, the prevalence of depression is moderately increased in prediabetic patients, and significantly higher in those previously diagnosed with T2DM [5]. A systematic review revealed that the occurrence of depression is two to three times more prevalent in individuals with T2DM than in those without diabetes [6]. Besides, research has confirmed that depression is significantly associated with increased all-cause mortality in patients with T2DM [7]. Both T2DM and depression are chronic diseases that constitute a substantial and increasing proportion of the global disease burden [8]. Given the high prevalence and impact of depression in individuals with T2DM, enhancing T2DM surveillance and identifying risk factors for the progression from incident T2DM to subsequent depression are crucial for implementing effective depression prevention strategies and interventions.

Loneliness and social isolation are the most formidable contemporary public health challenges [9]. Their prevalence is extensive, with approximately one-fourth of adults aged 65 years and older reporting social isolation and one-third of adults aged 45 years and older reporting feelings of loneliness [10]. Loneliness is primarily characterized by subjective emotional experiences of isolation, reflecting individuals’ authentic social interactions, while social isolation is determined by objective circumstances such as limited contact with others and disengagement from social groups and activities [11, 12]. Loneliness and social isolation are associated with a spectrum of adverse health consequences, encompassing T2DM and depression, which can impact individuals throughout their lifespan [13, 14]. Two large prospective cohorts conducted in Europe and East Asia revealed a significant association between loneliness, social isolation, and an increased risk of T2DM [15]. Both experimental and observational data support the notion that loneliness and social isolation can lead to immune inflammatory response and alterations in the hypothalamic–pituitary–adrenal (HPA) axis, potentially triggering the onset of T2DM and increasing the likelihood of severe hyperglycemic events that may lead to depression [16, 17]. However, limited research has systematically focused on the impact of loneliness and social isolation on the transitions from a healthy state to incident T2DM, then to subsequent depression, and finally to death, highlighting the need for further investigation to inform targeted prevention strategies for disease progression.

While loneliness and social isolation increase the risk of both depression and T2DM, the trajectory from incident T2DM to subsequent depression remains underexplored. Chronic inflammation links T2DM and depression, as T2DM induces elevation of TNF-α, IL-6, and high-sensitivity C-reactive protein (hsCRP), and elevated hsCRP is associated with depressive severity, poor treatment response, and suicidality, suggesting that T2DM-derived inflammation synergizes with loneliness- or isolation-amplified dysregulation to increase depression risk [18–21]. Furthermore, loneliness often precedes the onset of T2DM and is strongly associated with depression among diabetic patients [22, 23]. In particular, a T2DM diagnosis may interact with preexisting loneliness through chronic stress and metabolic burden to trigger subsequent depression [24]. Therefore, we conceptualize T2DM not as a mediator but as an intermediate disease state to examine the influence of loneliness and social isolation on the progression from incident T2DM to subsequent depression in the UK Biobank cohort.

To achieve this goal, we initially employed multistate models to explore the separate and joint effects of loneliness and social isolation on various disease transitions, including the progression from baseline to incident T2DM, then to subsequent depression, and ultimately to mortality. Additionally, we investigated the relationships between loneliness, social isolation, and the risk of transitioning from incident T2DM to subsequent depression under different glycemic metabolism statuses, including normal glucose tolerance (NGT) and prediabetes, while accounting for potential covariates. Finally, we adjusted the multistate models for glucose and lipid metabolic measures and excluded participants developing T2DM within the first year to validate the associations of loneliness and social isolation with the different disease transitions.

## Methods

### Study design and participants

This study utilized data from a largely prospective cohort sourced from the UK Biobank, which recruited over 500,000 participants aged 40–69 years between 2006 and 2010, conducting multiple follow-up assessments [25]. Upon enrollment, participants completed touchscreen questionnaires, underwent physical measurements, and submitted blood samples. Subsequent follow-ups were conducted regularly, with the database receiving frequent updates. Approval for the UK Biobank study was granted by the North West Multicenter Research Ethics Committee (11/NW/0382), and all participants provided written informed consent. This study is identified by application number 46478.

**Figure 1.**
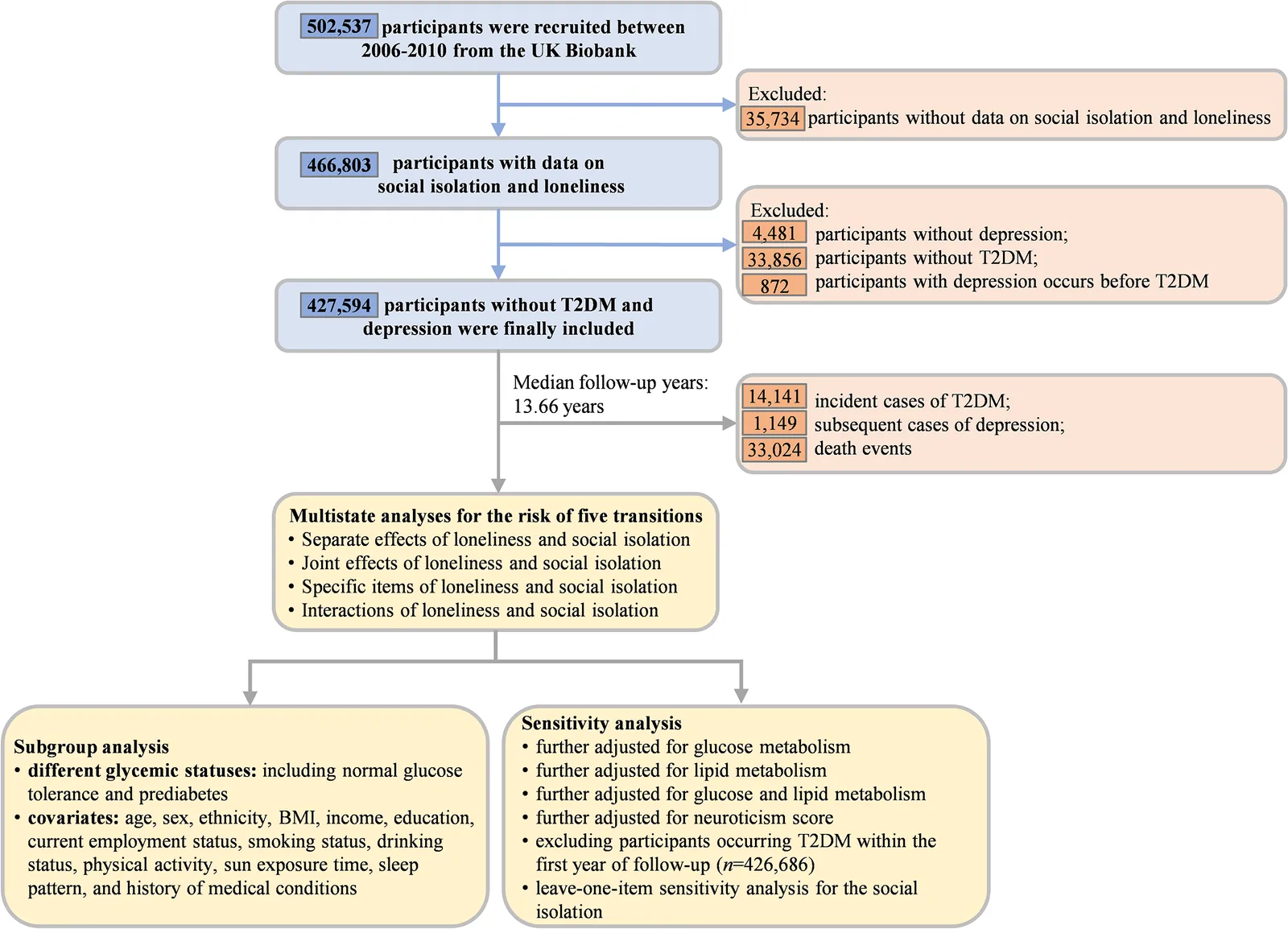
Flowchart of the study design: participant selection, multistate analysis, and robustness evaluation. BMI, body mass index; T2DM, type 2 diabetes mellitus. Figure 1. long description.

A total of 502,537 participants were included at baseline. We excluded 35,734 participants due to missing values related to loneliness and social isolation. Furthermore, we excluded 4,481 participants with a previous diagnosis of depression, 33,856 with a previous diagnosis of T2DM, and 872 participants who experienced depression before being diagnosed with T2DM. The diagnosis of T2DM at baseline was determined by the following criteria: a diagnosis made by doctor (Field ID 2443), use of anti-diabetic medications (Field ID 20003, 6153, and 6177), records from hospital inpatient admissions (E10–14), fasting blood glucose (FBG, Field ID 30740) ≥ 7.0 mmol/L, or glycosylated hemoglobin A1c (HbA1C, Field ID 30750) ≥ 6.5%. The diagnosis of depression at baseline was based on hospital inpatient records (F32–33). Ultimately, 427,594 participants were included to evaluate the impact of loneliness and social isolation on different disease transitions (Figure 1).

### Assessment of exposures

The variables examined in this study encompassed loneliness and social isolation. Loneliness was assessed using a scale ranging from 0 to 2 points, derived from responses to two specific questionnaire items: (1) “Do you often feel lonely?” (Field ID 2020). Participants indicating “yes” were assigned 1 point, while those responding “no” were allocated 0 points. (2) “How often are you able to confide in someone close to you?” (Field ID 2110). Participants reporting “never or almost never” were scored 1 point, whereas those reporting varying frequencies from “once every few months,” “once a month,” “once a week,” “2–4 times a week,” or “almost daily” received 0 points. Subsequently, participants were categorized into either the no-loneliness group (0–1 point) or the loneliness group (2 points) [15].

Social isolation scores were evaluated on a scale of 0–3 points, based on responses to three specific questions within the questionnaires: (1) “How often do you visit friends or family or have them visit you?” (Field ID 1031). Responses including “once a month,” “once every few months,” “never or almost never,” or “no friends or family outside the household” were scored 1 point, while responses like “once a week,” “2–4 times a week,” and “almost daily” were scored 0 points. (2) “Which of the following leisure or social activities do you engage in once a week or more often?” (Field ID 6160). Participants selecting “none of above” were scored 1 point, whereas those engaging in activities including “sports club or gym,” “pub or social club,” “religious group,” “adult education class,” or “other group activity” were scored 0 point. (3) “Including yourself, how many people live in your household? Include those who usually live in the house such as students living away from home during term time, and partners in the armed forces or in professions such as pilots” (Field ID 709). Respondents indicating “1” were given 1 point, while those reporting more than one individual were given 0 points. The scores from these questions were aggregated to determine the social isolation scores, leading to the classification of participants into the least isolated group (0 points), moderately isolated group (1 point), and most isolated group (2–3 points) [15].

### Assessment of outcome

The primary outcomes investigated in this study included incident T2DM, subsequent depression, and death. These outcomes were identified and confirmed through hospital admissions and death records data, which were obtained from the National Health Service (NHS), Hospital Episode Statistics (HES), and the International Classification of Diseases-10 (ICD-10; Field ID 41270 and 41280). Participants were diagnosed with T2DM using the code E11, while subsequent depression was identified using the codes F32 and F33. For participants without T2DM or depression at baseline of the study, follow-up continued until the end of their follow-up period, which was determined by the earliest occurrence of either the date of death (Field ID 40000), loss to follow-up (Field ID 191), or the end of the current follow-up period (October 31, 2022).

### Assessment of covariates

This study included a variety of covariates, encompassing age, sex, ethnicity, body mass index (BMI), household income, educational attainment, current employment status, smoking status, drinking status, physical activity, sun exposure time, healthy sleep score, history of medical conditions, glucose (FBG and HbA1c) or lipid metabolic measures obtained from laboratory tests on blood samples, and neuroticism score. Detailed descriptions of these covariates, along with their corresponding field IDs, can be found in Supplement 1 and Supplementary Table S1.

For missing covariate data, linear regression models were employed to impute continuous variables, while logistic regression models were utilized to impute categorical variables using the “Mice” package in R. Multiple imputations via chained equations with five imputations were conducted to fill in the missing values of covariates. The proportions of missing data for each covariate are presented in Supplementary Table S2.

### Statistical analyses

The baseline characteristics were presented as either the means ± standard errors or the medians (interquartile ranges) for continuous variables, and as frequencies (percentages) for categorical variables. When the continuous data met the assumptions of normal distribution and homogeneity of variance, statistical differences were evaluated using Student’s *t*-tests for comparisons between two groups and ANOVA tests for comparisons among three groups. Otherwise, comparisons between two groups were conducted using Mann–Whitney *U* tests, and comparisons among three groups were performed using Kruskal–Wallis *K* tests. For categorical variables, *χ* ^2^ tests were employed to examine the statistical variances.

**Figure 2.**
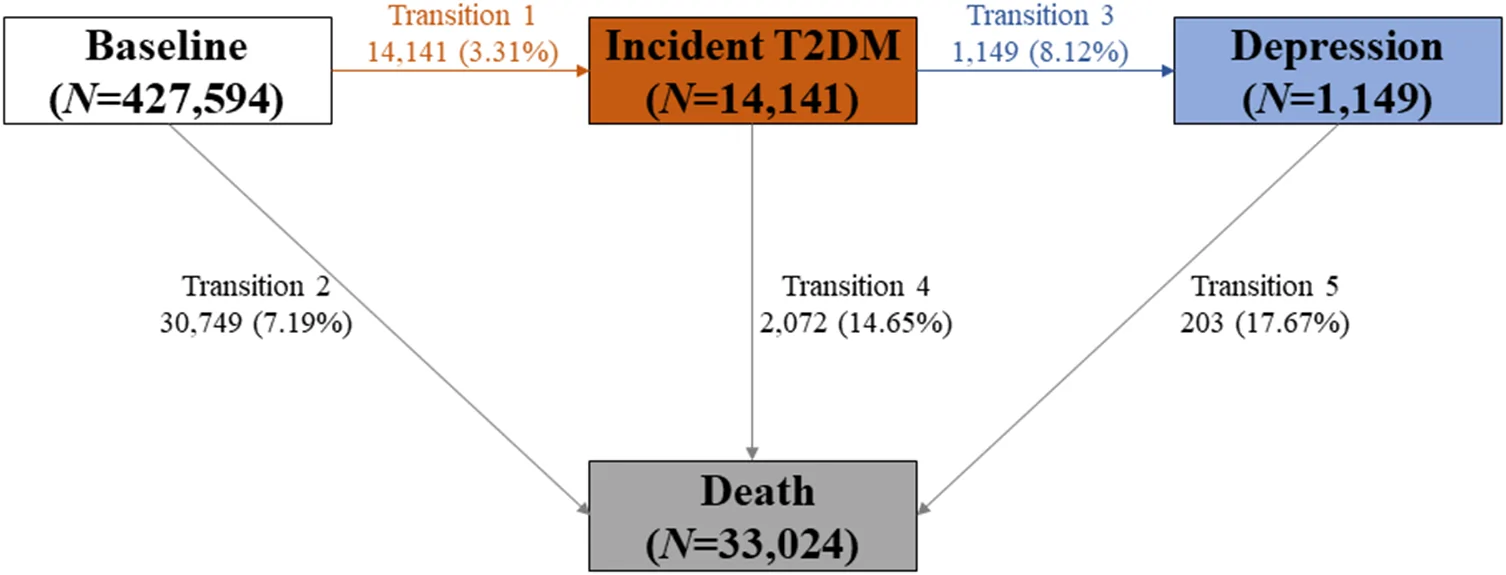
Numbers (percentages) of participants in transitions from baseline to incident type 2 diabetes mellitus (T2DM), further to subsequent depression and finally to death. Figure 2. long description.

Multistate models were employed to evaluate the effects of loneliness and social isolation on the progression from baseline to incident T2DM, further to subsequent depression, and ultimately leading to mortality. The multistate model, characterized by its probabilistic nature, accommodates multiple states, allowing for the analysis of transition rates between them [26, 27]. Expanding the Cox proportional hazards model to a multistate model enables the inclusion of multiple endpoints, providing a more comprehensive understanding of the disease trajectory [28]. Specifically, our study delineated five transitions within the multistate models (Figure 2), which include: (1) the transition from baseline to incident T2DM; (2) the transition from baseline to death; (3) the transition from incident T2DM to subsequent depression; (4) the transition from incident T2DM to death; and (5) the transition from subsequent depression to death. In cases where participants reported multiple diagnoses or events on the same day, we adopted the convention of considering the theoretical entry date of the preceding state as half a day before the entry date of the subsequent state, in accordance with previous research [26, 27, 29]. For instance, if a participant succumbed to incident T2DM, the date of the incident T2DM was recorded as half a day before the date of death. A variety of baseline covariates were included and categorized into two models: Model 1 adjusted for age, sex, ethnicity, BMI, household income, educational attainment, current employment status, smoking status, drinking status, physical activity, sun exposure time, and healthy sleep score; and Model 2, which additionally accounted for a history of cardiovascular disease, hypertension, and cancer. We examined the separate and joint associations of loneliness and social isolation with the risk of five transitions. Furthermore, we explored the specific items of loneliness and social isolation that influenced the risk of these transitions, as well as the trends in transition risks associated with increasing scores of loneliness and social isolation. In addition, we investigated the risk of the five transitions based on different levels of social isolation within varying categories of loneliness, and vice versa.

In addition to examining the entire population, we further explored the separate and joint associations of loneliness and social isolation on the risk of five transitions, as well as the interactions between loneliness and social isolation in relation to these transitions, among individuals with diverse glycemic statuses, including normal glucose tolerance (NGT) and prediabetes. The participants classified as having prediabetes met the criteria of a FBG level of 5.6–6.9 mmol/L or an HbA1c level of 5.7–6.4% [30].

Subgroup analyses were conducted based on sex (female or male), age (≤65 years or > 65 years), ethnicity (white or non-white), BMI (normal, overweight or obesity), household income (<£52,000 or ≥ £52,000), current employment status (employed or others), education level (college and above or others), smoking status (never or smoker), drinking status (never or drinker), physical activity (less than the median or above the median), sleep pattern (unhealthy or healthy), sun exposure time (less than the median or above the median), cardiovascular disease (no or yes), hypertension (no or yes), and cancer (no or yes). The subgroup analysis models were adjusted for the same covariates as Model 2, except for the stratification variable when used for stratification purposes. Interaction terms between the stratifying variable and loneliness or social isolation were included to assess differences between subgroups [31].

To ensure the reliability of our results, we conducted sensitivity analyses to examine the separate and combined effects of loneliness and social isolation on the risk of five transitions, as well as the interactions between loneliness and social isolation in relation to these transitions. We conducted additional models developed with adjustments for glucose metabolism (FBG and HbA1c), lipid metabolism (TC, TG, LDL, HDL, and Lp(a)), and glucose and lipid metabolism. Besides, participants diagnosed with T2DM within the first year of follow-up were excluded to minimize the potential impact of reverse causality (*n* = 426,686). Moreover, with additional adjustment for neuroticism score, we examined the separate and combined effects of loneliness and social isolation on the risk of five transitions. Finally, we performed a leave-one-item sensitivity analysis for the social isolation score. Specifically, we iteratively removed each component item of the social isolation score and recalculated the sum score using the remaining items. We examined the association between each reduced score and the risk of five transitions.

Data cleaning and analyses were performed using R version 4.4.1 in this study. The “mstate” R package was predominantly utilized to implement the multistate models. Statistical significance was defined as a *p*-value of <0.05.

## Results

### Baseline characteristics

A total of 427,594 participants were included in this study. The median age of the participants was 57 years, and 44.5% were male. The baseline characteristics of participants, categorized by loneliness and social isolation and various disease statuses during follow-up, are presented in Table 1 and Supplementary Table S3, respectively. Individuals with higher levels of social isolation or loneliness tended to be male, have obesity, have a low household income, have a history of cardiovascular disease or hypertension, and exhibit unhealthier lifestyle behaviors, including being previous/current smokers, less physical activity, and unhealthier sleep patterns.

**Table 1.** Baseline characteristics of the study population grouped by loneliness and social isolation Table 1. long description.

Variables	All	Loneliness			Social isolation			
		No loneliness	Loneliness	*P*-value	Least isolated	Moderate isolated	Most isolated	*P*-value
*N*	427,594	408,434	19,160		196,010	172,200	59,384	
Sex, *n* (%)				<0.001				< 0.001
Female	237,308 (55.5)	227,657 (55.7)	9,651 (50.4)		110,785 (56.5)	96,355 (56.0)	30,168 (50.8)	
Male	190,286 (44.5)	180,777 (44.3)	9,509 (49.6)		85,225 (43.5)	75,845 (44.0)	29,216 (49.2)	
Age, median (Q1, Q3)	57 (50, 63)	57 (50, 63)	56 (49, 62)	<0.001	58 (50, 63)	57 (50, 63)	57 (49, 62)	<0.001
Ethnicity, *n* (%)				<0.001				<0.001
White	409,647 (95.8)	391,533 (95.9)	18,114 (94.5)		189,265 (96.6)	164,553 (95.6)	55,829 (94.0)	
Non-white	17,947 (4.2)	16,901 (4.1)	1,046 (5.5)		6,745 (3.4)	7,647 (4.4)	3,555 (6.0)	
BMI, median (Q1, Q3)	26.6 (24, 29.4)	26.6 (24, 29.3)	27.1 (24.5, 30.4)	<0.001	26.5 (24.1, 29.2)	26.6 (24, 29.5)	26.7 (23.9, 29.7)	<0.001
Household income, *n* (%)				<0.001				<0.001
Low	311,409 (72.8)	295,659 (72.4)	15,750 (82.2)		136,586 (69.7)	128,383 (74.6)	46,440 (78.2)	
High	116,185 (27.2)	112,775 (27.6)	3,410 (17.8)		59,424 (30.3)	43,817 (25.4)	12,944 (21.8)	
Current employment status, *n* (%)				<0.001				<0.001
Employed	255,947 (59.9)	245,081 (60.0)	10,866 (56.7)		112,480 (57.4)	105,412 (61.2)	38,055 (64.1)	
Others	171,647 (40.1)	163,353 (40.0)	8,294 (43.3)		83,530 (42.6)	66,788 (38.8)	21,329 (35.9)	
Education level, *n* (%)				<0.001				<0.001
College or above	172,534 (40.3)	165,962 (40.6)	6,572 (34.3)		77,752 (39.7)	69,614 (40.4)	25,168 (42.4)	
Others	255,060 (59.7)	242,472 (59.4)	12,588 (65.7)		118,258 (60.3)	102,586 (59.6)	34,216 (57.6)	
Smoking status, *n* (%)				<0.001				<0.001
Never	237,088 (55.4)	227,696 (55.7)	9,392 (49.0)		111,979 (57.1)	94,620 (54.9)	30,489 (51.3)	
Previous	146,814 (34.3)	140,378 (34.4)	6,436 (33.6)		68,610 (35)	58,663 (34.1)	19,541 (32.9)	
Current	43,692 (10.2)	40,360 (9.9)	3,332 (17.4)		15,421 (7.9)	18,917 (11)	9,354 (15.8)	
Drinking status, *n* (%)				<0.001				<0.001
Never	16,077 (3.8)	15178 (3.7)	899 (4.7)		6,453 (3.3)	6,706 (3.9)	2,918 (4.9)	
Previous	13,690 (3.2)	12,702 (3.1)	988 (5.2)		4,557 (2.3)	5,819 (3.4)	3,314 (5.6)	
Current	397,827 (93.0)	380,554 (93.2)	17,273 (90.2)		185,000 (94.4)	159,675 (92.7)	53,152 (89.5)	
Physical activity, *n* (%)				<0.001				<0.001
Low	213,797 (50.0)	203,427 (49.8)	10,370 (54.1)		89,921 (45.9)	89,499 (52.0)	34,377 (57.9)	
High	213,797 (50.0)	205,007 (50.2)	8,790 (45.9)		106,089 (54.1)	82,701 (48.0)	25,007 (42.1)	
Sleep pattern, *n* (%)				<0.001				<0.001
Unhealthy	213,823 (50.0)	201,068 (49.2)	12,755 (66.6)		92,335 (47.1)	87,818 (51.0)	33,670 (56.7)	
Healthy	213,771 (50.0)	207,366 (50.8)	6,405 (33.4)		103,675 (52.9)	84,382 (49.0)	25,714 (43.3)	
Sun exposure time, Median (Q1, Q3)	2.5 (1.5, 3.5)	2.5 (1.5, 3.5)	2.5 (1.5, 3.5)	<0.001	2.5 (1.5, 4.0)	2.5 (1.5, 3.5)	2.5 (1.5, 3.5)	<0.001
CVD, *n* (%)				<0.001				<0.001
No	288,756 (67.5)	276,953 (67.8)	11,803 (61.6)		133,073 (67.9)	116,228 (67.5)	39,455 (66.4)	
Yes	138,838 (32.5)	131,481 (32.2)	7,357 (38.4)		62,937 (32.1)	55,972 (32.5)	19,929 (33.6)	
Hypertension, *n* (%)				<0.001				<0.001
No	271,077 (63.4)	260,087 (63.7)	10,990 (57.4)		125,750 (64.2)	108,547 (63.0)	36,780 (61.9)	
Yes	156,517 (36.6)	148,347 (36.3)	8,170 (42.6)		70,260 (35.8)	63,653 (37.0)	22,604 (38.1)	
Cancer, *n* (%)				<0.001				<0.001
No	332,842 (77.8)	317,727 (77.8)	15,115 (78.9)		152,125 (77.6)	134,224 (77.9)	46,493 (78.3)	
Yes	94,752 (22.2)	90,707 (22.2)	4,045 (21.1)		43,885 (22.4)	37,976 (22.1)	12,891 (21.7)	
FBG, mmol/L	4.9 (4.6, 5.2)	4.9 (4.6, 5.2)	4.9 (4.6, 5.2)	0.021	4.9 (4.6, 5.2)	4.9 (4.6, 5.2)	4.9 (4.6, 5.2)	0.002
HbA1c, %	5.3 (5.2, 5.5)	5.3 (5.2, 5.5)	5.4 (5.2, 5.6)	<0.001	5.3 (5.1, 5.5)	5.3 (5.2, 5.6)	5.3 (5.2, 5.6)	<0.001
TC, mmol/L	5.8 (5.1, 6.4)	5.8 (5.1, 6.4)	5.8 (5.0, 6.4)	<0.001	5.8 (5.1, 6.4)	5.8 (5.1, 6.4)	5.8 (5.0, 6.4)	<0.001
TG, mmol/L	1.5 (1.1, 2.1)	1.5 (1.1, 2.0)	1.6 (1.1, 2.2)	<0.001	1.5 (1.1, 2.0)	1.5 (1.1, 2.1)	1.6 (1.1, 2.1)	<0.001
LDL, mmol/L	3.6 (3.1, 4.1)	3.6 (3.1, 4.1)	3.6 (3.0, 4.1)	0.585	3.6 (3.1, 4.1)	3.6 (3.1, 4.1)	3.6 (3.1, 4.1)	0.664
HDL, mmol/L	1.5 (1.2, 1.6)	1.5 (1.2, 1.7)	1.4 (1.2, 1.6)	<0.001	1.5 (1.2, 1.7)	1.5 (1.2, 1.6)	1.5 (1.2, 1.6)	<0.001
Lp(a), mmol/L	41.1 (12.1, 44.9)	41.1 (12.1, 44.9)	42.3 (12.3, 44.9)	0.862	41.0 (12.1, 44.9)	41.2 (12.2, 44.9)	41.2 (12, 44.9)	0.417
Neuroticism score, Median (Q1, Q3)	4.1 (2, 6)	4 (2, 5)	7(4.1, 10)	<0.001	4 (2, 5)	4.1 (2, 6)	4.1 (2, 6)	<0.001

Abbreviations: CVD, cardiovascular disease; FBG, fasting blood glucose; HbA1c, glycated hemoglobin; HDL, high-density lipoprotein cholesterol; LDL, low-density lipoprotein cholesterol; Lp(a), lipoprotein A; T2DM, type 2 diabetes mellitus; TC, cholesterol; TG, triglycerides.

Over a median follow-up period of 13.66 years, there were 14,141 documented cases of incident T2DM, 1,149 subsequent cases of depression, and 33,024 recorded death events. Participants who experienced adverse events tended to exhibit higher levels of loneliness and social isolation, obesity, low educational attainment, low household income, a history of smoking (either previous or current), and a history of cardiovascular disease, hypertension, or cancer.

### Loneliness, social isolation, risk of disease transitions: multistate analyses

The multistate analyses revealed that loneliness and social isolation were associated with varying degrees of increased risk for disease progression, with the exception of the transition from subsequent depression to death (Table 2 and Supplementary Table S4). Notably, the impact of loneliness and social isolation on the progression from incident T2DM to subsequent depression was most pronounced among the four significant transitions. Notably, for the transition from incident T2DM to subsequent depression, individuals in the loneliness group exhibited a significantly higher risk, with a hazard ratio (HR) of 1.802 (95% confidence interval, CI: 1.521–2.135), compared to those in the no-loneliness group after adjusting for sociodemographic characteristics and lifestyle factors in Model 1 (Table 2). This HR slightly decreased to 1.793 (1.513–2.124) after accounting for comorbidities in Model 2 (Supplementary Table S4). Similarly, participants in the most isolated group had an HR of 1.390 (1.185–1.632) compared to those in the least isolated group, with a significant trend (P-trend = 1.08 × 10^−4^) in Model 1 (Table 2).

**Table 2.** Separate and joint association of loneliness and social isolation with the risk of five transitions (Model 1) Table 2. long description.

	*N*	Baseline to incident T2DM		Baseline to death		Incident T2DM to subsequent depression		Incident T2DM to death		Subsequent depression to death	
		HR (95% CI)	*P*-value	HR (95% CI)	*P*-value	HR (95% CI)	*P*-value	HR (95% CI)	*P-*value	HR (95% CI)	*P*-value
Separate effects											
Loneliness											
No loneliness	408,434	As reference		As reference		As reference		As reference		As reference	
Loneliness	19,160	**1.240 (1.161, 1.324)**	**1.50 × 10** ^**−10**^	**1.196 (1.139, 1.255)**	**4.54 × 10** ^**−13**^	**1.802 (1.521, 2.135)**	**1.01 × 10** ^**−11**^	0.999 (0.841, 1.186)	9.89 × 10^−1^	1.131 (0.758, 1.687)	5.46 × 10^−1^
Social isolation											
Least isolated	196,010	As reference		As reference		As reference				As reference	
Moderate isolated	172,200	**1.094 (1.054, 1.134)**	**1.53 × 10** ^**−6**^	**1.152 (1.123, 1.181)**	**1.03 × 10** ^**−28**^	1.103 (0.965, 1.261)	1.52 × 10^−1^	**1.186 (1.077, 1.306)**	**5.47 × 10** ^**−4**^	0.922 (0.666, 1.276)	6.23 × 10^−1^
Most isolated	59,384	**1.184 (1.128, 1.243)**	**9.99 × 10** ^**−12**^	**1.405 (1.360, 1.452)**	**5.88 × 10** ^**−93**^	**1.390 (1.185, 1.632)**	**5.31 × 10** ^**−5**^	**1.249 (1.102, 1.417)**	**5.26 × 10** ^**−4**^	1.158 (0.802, 1.671)	4.34 × 10^−1^
*P* trend			**6.94 × 10** ^**−13**^		**1.35 × 10** ^**−92**^		**1.08 × 10** ^**−4**^		**9.13 × 10** ^**−5**^		5.11 × 10^−1^
Joint effects											
No loneliness											
Least isolated	191,058	As reference		As reference		As reference		As reference		As reference	
Moderate isolated	163,986	**1.089 (1.049, 1.131)**	**8.34 × 10** ^**−6**^	**1.147 (1.118, 1.176)**	**5.92 × 10** ^**−26**^	1.091 (0.947, 1.256)	2.29 × 10^−1^	**1.198 (1.085, 1.322)**	**3.59 × 10** ^**−4**^	0.909 (0.645, 1.281)	5.86 × 10^−1^
Most isolated	53,390	**1.166 (1.107, 1.227)**	**5.95 × 10** ^**−9**^	**1.394 (1.347, 1.442)**	**5.56 × 10** ^**−81**^	**1.331 (1.116, 1.587)**	**1.44 × 10** ^**−3**^	**1.261 (1.104, 1.44)**	**6.36 × 10** ^**−4**^	1.133 (0.756, 1.698)	5.45 × 10^−1^
Loneliness											
Least isolated	4,952	**1.219 (1.064, 1.398)**	**4.47 × 10** ^**−3**^	1.102 (0.99, 1.227)	7.43 × 10^−2^	**1.958 (1.369, 2.799)**	**2.30 × 10** ^**−4**^	1.092 (0.762, 1.565)	6.33 × 10^−1^	1.019 (0.439, 2.367)	9.65 × 10^−1^
Moderate isolated	8,214	**1.312 (1.186, 1.452)**	**1.28 × 10** ^**−7**^	**1.319 (1.224, 1.422)**	**3.60 × 10** ^**−13**^	**1.876 (1.434, 2.454)**	**4.38 × 10** ^**−6**^	1.076 (0.817, 1.418)	6.00 × 10^−1^	1.035 (0.528, 2.030)	9.20 × 10^−1^
Most isolated	5,994	**1.398 (1.253, 1.560)**	**1.85 × 10** ^**−9**^	**1.540 (1.424, 1.665)**	**1.90 × 10** ^**−27**^	**2.127 (1.620, 2.792)**	**5.38 × 10** ^**−8**^	1.209 (0.918, 1.592)	1.76 × 10^−1^	1.253 (0.681, 2.303)	4.69 × 10^−1^
*P* trend			**8.94 × 10** ^**−20**^		**4.07 × 10** ^**−79**^		**3.92 × 10** ^**−13**^		**1.23 × 10** ^**−2**^		4.12 × 10^−1^

Abbreviations: CI, confidence interval; HR, hazard ratio; T2DM, type 2 diabetes mellitus.

In terms of the joint effects of loneliness and social isolation, individuals experiencing both loneliness and the most isolated had a significantly elevated risk of transitioning from incident T2DM to subsequent depression, with an HR of 2.127 (1.620–2.792) compared to those in the no-loneliness and least isolated group. Furthermore, the analysis of potential interactions between loneliness and social isolation revealed that loneliness had a greater effect size than social isolation across the five transitions (Supplementary Tables S5 and S6).

In addition, positive dose-gradient associations were identified between loneliness and social isolation scores and an increased risk of transitions from baseline to incident T2DM, from baseline to death, and from T2DM to subsequent depression (*P* trend < 0.001; Supplementary Tables S7 and S8). Furthermore, for the items related to loneliness and social isolation, most of the specific elements significantly associated with an increased risk of transitioning from incident T2DM to subsequent depression in Model 2, including feeling lonely (HR = 1.786, 95% CI: 1.578–2.021), being unwilling to confide (HR = 1.282, 95% CI: 1.115–1.475), less social activities (HR = 1.176, 95% CI: 1.043–1.327), and living alone (HR = 1.214, 95% CI: 1.061–1.388) (Supplementary Table S8).

### Loneliness, social isolation, risk of disease transitions: under different glycemic statuses

**Figure 3.**
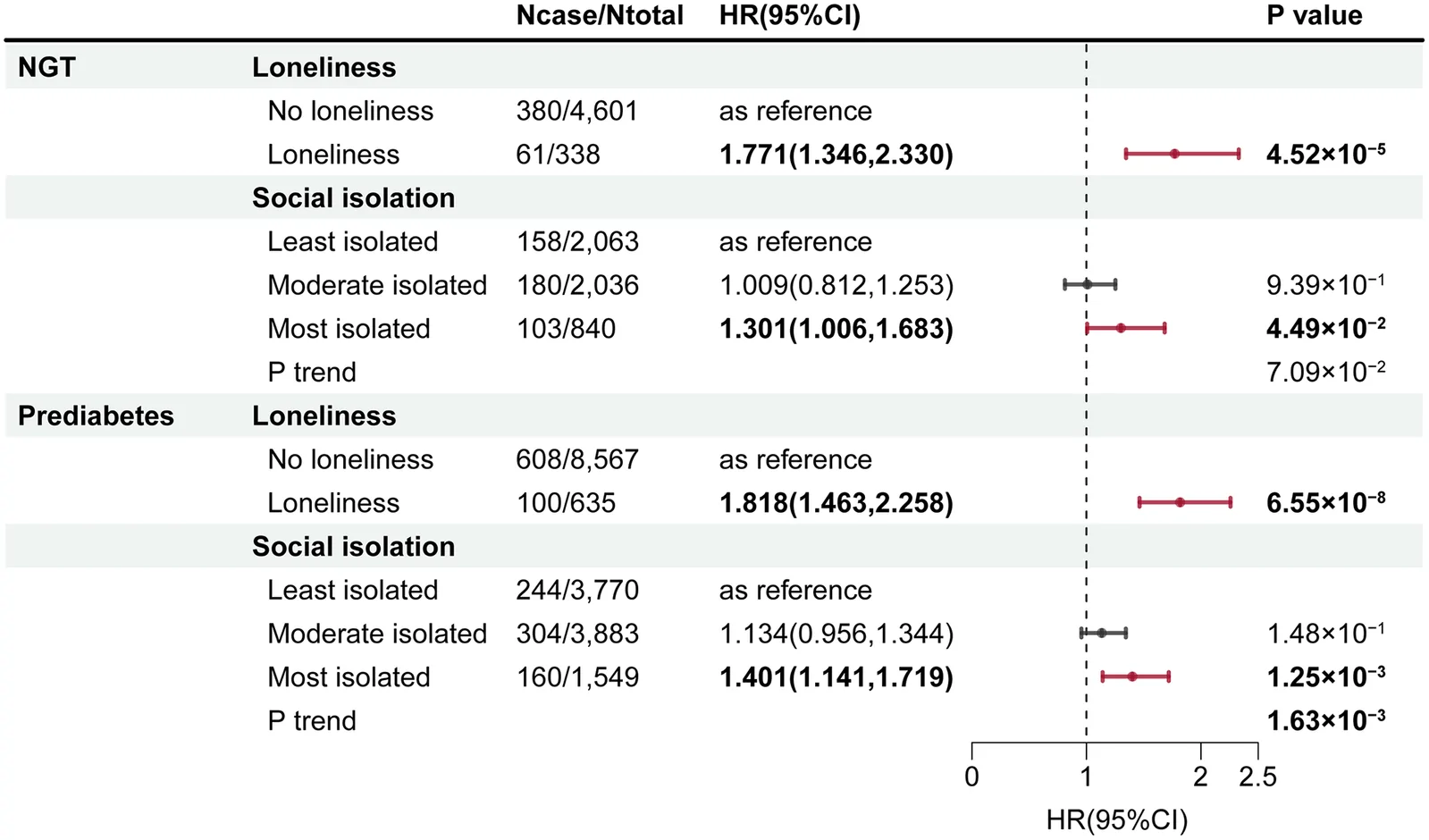
Separate effects of social isolation and loneliness on the risk of depression among subjects with T2DM under different glycemic statuses (Model 2). The analysis was performed in Model 2 (adjusted for sex, age, ethnicity, body mass index, household income, current employment status, education level, smoking status, drinking status, physical activity, sleep pattern, sun exposure time, history of CVD, history of hypertension, and history of cancer). Abbreviations: CI, confidence interval; CVD, cardiovascular disease; HR, hazard ratio; NGT, normal glucose tolerance; T2DM, type 2 diabetes mellitus. Figure 3. long description.

Regardless of participants’ glycemic status, the association between loneliness, social isolation, and increased risk of disease progression remained statistically significant, except for the transition from subsequent depression to death (Figure 3; Supplementary Table S9). The participants with prediabetes exhibited a greater risk of incident depression among T2DM compared to those with normal glucose tolerance (loneliness: HR [95% CI] = 1.818 [1.463–2.258] in prediabetes vs. 1.771 [1.346–2.330] in NGT; social isolation: HR [95% CI] = 1.401 [1.141–1.719] in prediabetes vs. 1.301 [1.006–1.683] in NGT for the most isolated). However, loneliness and social isolation in participants with normal glucose tolerance (HR [95% CI] = 1.172 [1.048–1.310] for loneliness; 1.067 [1.003–1.135] for the moderate isolated and 1.205 [1.110–1.308] for the most isolated) were more strongly associated with the risk of the transition from baseline to incident T2DM than in those with prediabetes (HR [95% CI] = 1.119 [1.031–1.214] for loneliness; 1.060 [1.013–1.109] for the moderate isolated and 1.092 [1.028–1.160] for the most isolated).

Joint analysis for social isolation and loneliness indicated a positive correlation between loneliness, social isolation, and the risk of transitioning from incident T2DM to subsequent depression in both the normal glucose tolerance and prediabetes groups (*P* trend<0.001; Supplementary Table S10). Moreover, the impact of loneliness and social isolation was more pronounced in the prediabetes group compared to the normal glucose tolerance group (Supplementary Tables S10 and S11).

### Subgroup analysis

Subgroup analyses for the separate associations of social isolation and loneliness with risk of five transitions are presented in Supplementary Tables S12 and S13. No significant interactions were observed between the majority of covariates and either social isolation or loneliness related to the risk of five transitions. However, employment status (*P*_interaction_ = 0.001) and having a history of cancer (*P*_interaction_ < 0.001) exhibited interactions with loneliness in the transition from baseline to death. On the other hand, covariates, including sex, BMI, household income, employment status, smoking status, physical activity, and a history of cancer, displayed significant interactions with social isolation in the transition from baseline to death. Additionally, ethnicity and drinking status had interactions with social isolation in the transition from baseline to incident T2DM.

### Sensitivity analysis

For separate associations, joint associations, and potential interactions of loneliness and social isolation in relation to the five transitions, the findings were consistent in models that included additional adjustments for glucose metabolism, lipid metabolism, as well as glucose and lipid metabolic biomarkers (Supplementary Tables S14–S19). Furthermore, even after excluding participants diagnosed with T2DM within the first year, our results retained their significance (Supplementary Tables S20 and 21). Besides, the results remained consistent after additional adjustment for neuroticism score, confirming that our main conclusions were not confounded by this personality trait (Supplementary Table S22). Moreover, the leave-one-item analysis for the social isolation score yielded consistent effect estimates across all iterations, with no single item driving the observed associations (Supplementary Table S23).

## Discussion

In this study, we have discovered that both loneliness and social isolation are found to be independently and jointly associated with varying degrees of increased risk for disease progression, as indicated by multistate analyses, except for the progression from subsequent depression to death. Particularly striking was the pronounced impact of loneliness and social isolation on the transition from incident T2DM to subsequent depression among the four significant transitions observed. The relationships between social isolation, loneliness, and the increased risk of transitioning from incident T2DM to subsequent depression remained statistically significant even after adjusting for glucose and lipid metabolic biomarkers in the models. Furthermore, the connections between loneliness, social isolation, and the elevated risk of progressing from incident T2DM to subsequent depression were notably stronger in individuals with prediabetes compared to those with normal glucose tolerance.

Consistent with previous research findings, our results indicate that loneliness and social isolation correlate with an increased risk of T2DM and depression. Two longitudinal studies demonstrated that individuals experiencing social isolation and loneliness faced an ~20% greater risk of developing incident T2DM, independent of the genetic predisposition to T2DM [15, 32]. Furthermore, individuals with greater social isolation exhibited a higher prevalence of both newly diagnosed and previously diagnosed T2DM, while a lack of social engagement was linked to pre-diabetes and previously diagnosed T2DM [33]. Additionally, previous investigations have highlighted that individuals who are socially inactive and lonely are at a heightened risk of depression compared to those with socially active and non-lonely [34, 35]. From a clinical perspective, these findings suggest that routine screening for social isolation and loneliness could be integrated into primary care settings, particularly for individuals at risk of metabolic or mental disorders, enabling early detection and targeted psychosocial support.

In addition, our findings revealed that loneliness and social isolation could increase the risk of transitioning from incident T2DM to subsequent depression, with loneliness exerting a more pronounced influence on this progression compared to social isolation. Network analysis highlighted that loneliness was predominantly influenced by social isolation [36]. The transition from social isolation to loneliness may link metabolic dysregulation to affective disturbance through several interconnected pathways. Both loneliness and social isolation have been associated with chronic stress, hyperactivation of the HPA axis, and elevated pro-inflammatory cytokines, like interleukin-6 and C-reactive protein [37–40]. Among patients with T2DM, loneliness may amplify maladaptive cognitive appraisals, such as perceived loss of control and stigma, perpetuating HPA axis dysfunction and disrupting glucose–insulin homeostasis, thereby creating a bidirectional metabolic–affective loop [24]. Moreover, loneliness and social isolation correlate with poorer medication adherence, dietary compliance, and physical activity, leading to suboptimal glycemic control, a factor significantly linked to depression [41–43]. Loneliness also erodes a sense of mastery and belonging, reducing diabetes-related self-efficacy and fostering helplessness [44]. Furthermore, socially isolated individuals are less likely to attend regular follow-ups or seek help for emerging mood symptoms [45]. Understanding the interplay between loneliness, social isolation, and the progression from incident T2DM to subsequent depression could provide valuable insights for the prevention of depression in individuals with T2DM. A key public health implication is that interventions targeting loneliness, such as cognitive-behavioral therapy for social cognition or peer-support programs, might be more effective than simply reducing objective social isolation, particularly among recently diagnosed patients.

Previous studies have demonstrated that social isolation and loneliness are more strongly associated with an increased risk of T2DM in individuals with normoglycemia than in those with prediabetes, a finding consistent with our results [13]. This suggests that social isolation and loneliness may independently contribute to a higher risk of T2DM in individuals without prediabetes. However, our study further revealed that, compared to individuals with normal glucose tolerance, those with prediabetes exhibited a significantly higher risk of incident depression among patients with T2DM when analyzed across different glycemic statuses. This underscores the pivotal role of glycemic status as a critical determinant in the development of depression, particularly in prediabetes [46]. Emerging evidence suggests that hyperglycemia and hyperinsulinemia in prediabetes may induce neuroendocrine alterations, which could ultimately contribute to the pathogenesis of depression [47, 48]. Furthermore, research has demonstrated that glycemic variability is closely associated with cognitive impairment, a key factor implicated in the progression of depression [49, 50]. Clinically, these findings highlight that individuals with prediabetes should be considered a high-risk group for depression following T2DM diagnosis, warranting routine mental health surveillance and early psychological intervention even before overt T2DM develops. From a public health standpoint, integrating loneliness assessment into diabetes prevention programs could improve both metabolic and mental health outcomes simultaneously.

The findings also highlight significant differences in the impact of loneliness and social isolation across various subgroups. The adverse effects of loneliness and social isolation on mortality were stronger among unemployed individuals, partly due to the protective role of purposeful employment [51], but weaker among cancer patients, who often adopt healthier lifestyles [52]. Social isolation also posed a higher risk of incident T2DM in drinkers than in nondrinkers, suggesting that alcohol consumption may exacerbate the adverse health outcomes [53]. In young adults, problematic digital use is linked to emotional loneliness and boredom, which mediate the relationship between depressive symptoms and digital behaviors [54, 55]. Extending this, younger individuals may be more susceptible to loneliness- and isolation-related distress, potentially strengthening the transition from new-onset T2DM to depression. These findings highlight the need to consider individual circumstances and behaviors when assessing loneliness and social isolation across disease transitions. Accordingly, age-specific strategies targeting emotional regulation and boredom coping may help interrupt the pathway from T2DM onset to depression in younger populations.

Several limitations should be acknowledged. First, loneliness and social isolation were assessed only at baseline using brief, nonvalidated indices, failing to capture their temporal dynamics or multidimensional nature. Future studies should employ repeated, validated measures and serial biomarkers to test mediation. Second, depression was defined using hospital-recorded ICD-10 codes (F32–F33), capturing primarily moderate-to-severe cases and missing milder or primary-care-managed depression. Moreover, delays between symptom onset and recorded diagnosis may introduce nondifferential misclassification of outcome timing. Third, despite covariate adjustment, residual confounding from unmeasured factors cannot be excluded. Additionally, although multiple imputation was appropriately performed, high missing rates may introduce imputation uncertainty. Fourth, while participants with T2DM diagnosed within the first follow-up year were excluded, prodromal or subclinical depression at baseline may still have influenced self-reported loneliness or social isolation, leaving directionality unresolved. Fifth, loneliness and depression share phenomenological features, raising construct overlap concerns. Although we adjusted for baseline mental health indicators, this conceptual proximity limits causal interpretation. Consequently, our findings represent robust associations rather than proof of causality, which cannot be established from this observational study alone. Lastly, the UK Biobank cohort is predominantly White, healthier, and of higher socioeconomic status than the general population [56]. Thus, generalizability to more diverse, deprived, or clinically vulnerable populations may be limited, though internal validity remains strong.

In conclusion, our findings demonstrate that loneliness and social isolation are significantly associated with an elevated risk of progression from baseline to incident T2DM, to subsequent depression and further to death. Notably, the impact of loneliness and social isolation on the transition from incident T2DM to subsequent complications was more pronounced among individuals with prediabetes compared to those with normal glucose tolerance. A more comprehensive understanding of these associations can guide the development of targeted interventions aimed at mitigating loneliness and social isolation, thereby reducing the risk of disease progression and mortality.

## Supporting information

10.1192/j.eurpsy.2026.12244.sm001 Qi et al. supplementary material Qi et al. supplementary material

## Data Availability

All of our study samples were derived from the UK Biobank (application ID 46478). The UKB data are available through the UK Biobank Access Management System (https://www.ukbiobank.ac.uk/).

## Supplementary material

The supplementary material for this article can be found at http://doi.org/10.1192/j.eurpsy.2026.12244.

## Long descriptions

### Figure 1 Long description

The flowchart begins at the top with 502,537 participants recruited between 2006-2010 from the UK Biobank.

An arrow points down to the next stage, while a side arrow to the right indicates the exclusion of 35,734 participants without data on social isolation and loneliness.

The second central box shows 466,803 participants with data on social isolation and loneliness. An arrow points down, while a side arrow to the right indicates exclusions: 4,481 participants without depression; 33,856 participants without T2 DM; and 872 participants where depression occurred before T2 D M.

The third central box shows 427,594 participants without T2 DM and depression were finally included. Below this, a median follow-up of 13.66 years is noted. A side arrow to the right lists outcomes: 14,141 incident cases of T2 DM; 1,149 subsequent cases of depression; and 33,024 death events.

The fourth central box describes Multistate analyses for the risk of five transitions, including:

* Separate effects of loneliness and social isolation

* Joint effects of loneliness and social isolation

* Specific items of loneliness and social isolation

* Interactions of loneliness and social isolation

The flow then splits into two parallel boxes at the bottom:

1. Left box: Subgroup analysis covering different glycemic statuses and covariates such as age, sex, ethnicity, BM I, income, education, employment, smoking, drinking, physical activity, sun exposure, sleep, and medical history.

2. Right box: Sensitivity analysis including adjustments for glucose and lipid metabolism, neuroticism score, exclusion of early T2 DM cases (n=426,686), and leave-one-item analysis for social isolation.

Navigate back to Figure 1.

### Figure 2 Long description

The flowchart consists of four rectangular boxes representing health states and five labeled arrows representing transitions.

Health States:

* Baseline: White box at the top left, N equals 427,594.

* Incident T2 DM: Orange box at the top center, N equals 14,141.

* Depression: Blue box at the top right, N equals 1,149.

* Death: Gray box at the bottom center, N equals 33,024.

Transitions:

* Transition 1: An orange arrow points from Baseline to Incident T2 D M, labeled 14,141 or 3.31 percent.

* Transition 2: A gray arrow points from Baseline down to Death, labeled 30,749 or 7.19 percent.

* Transition 3: A blue arrow points from Incident T2 DM to Depression, labeled 1,149 or 8.12 percent.

* Transition 4: A gray arrow points from Incident T2 DM down to Death, labeled 2,072 or 14.65 percent.

* Transition 5: A gray arrow points from Depression down to Death, labeled 203 or 17.67 percent.

Navigate back to Figure 2.

### Table 1 Long description

The table is structured with columns for All participants, Loneliness (No loneliness, Loneliness, P-value), and Social isolation (Least isolated, Moderate isolated, Most isolated, P-value).

Key data points include:

* Sample size (N): Total 427,594. Loneliness group: 19,160. Most isolated group: 59,384.

* Sex: Females make up 55.5% of the total population but only 50.4% of the loneliness group.

* Age: Median age is 57 across most groups.

* Ethnicity: 95.8% are White.

* Household income: 72.8% are in the Low category. This rises to 82.2% in the loneliness group and 78.2% in the most isolated group.

* Lifestyle factors: The loneliness group has a higher percentage of current smokers (17.4% vs 9.9% in the no loneliness group) and unhealthy sleep patterns (66.6% vs 49.2%).

* Clinical variables: P-values for most characteristics (Sex, Age, Ethnicity, BM I, Income, Employment, Education, Smoking, Drinking, Physical activity, Sleep, CV D, Hypertension, Cancer, HbA1 c, T C, T G, HD L, and Neuroticism) are less than 0.001, indicating significant differences across groups.

* Neuroticism score: Significantly higher in the loneliness group (median 7) compared to the no loneliness group (median 4).

Navigate back to Table 1.

### Table 2. Long description

The table is structured with 12 columns. The first column lists categories, the second lists sample size N, and the remaining 10 columns are paired as H R (95% CI) and P-value for five transitions: Baseline to incident T2 D M, Baseline to death, Incident T2 DM to subsequent depression, Incident T2 DM to death, and Subsequent depression to death.

Key data points from the Separate effects section:

* Loneliness: For the transition from Baseline to incident T2 D M, the HR is 1.240 (1.161, 1.324) with a P-value of 1.50 times 10 super minus 10. For Baseline to death, the HR is 1.196 (1.139, 1.255).

* Social isolation: The ‘Most isolated’ group shows an HR of 1.184 for T2 DM and 1.405 for death compared to the ‘Least isolated’ reference group. The P trend for social isolation across most transitions is highly significant (e.g., 6.94 times 10 super minus 13 for T2 DM).

Key data points from the Joint effects section:

* No loneliness and Most isolated: HR for Baseline to incident T2 DM is 1.166 (1.107, 1.227).

* Loneliness and Most isolated: This group shows the highest risks, with an HR of 1.398 (1.253, 1.560) for Baseline to incident T2 D M, 1.540 (1.424, 1.665) for Baseline to death, and 2.127 (1.620, 2.792) for Incident T2 DM to subsequent depression.

Boldface indicates statistically significant associations. Abbreviations used: HR for hazard ratio, CI for confidence interval, and T2 DM for type 2 diabetes mellitus.

Navigate back to Table 2..

### Figure 3 Long description

The forest plot is divided into two primary vertical sections: NGT and Prediabetes. A vertical dashed line at HR equals 1 serves as the null effect reference.

In the NGT section:

* Loneliness: ‘No loneliness’ is the reference. ‘Loneliness’ has an HR of 1.771 (1.346, 2.330) with a P value of 4.52 times 10 super minus 5. The error bar is entirely to the right of the null line.

* Social isolation: ‘Least isolated’ is the reference. ‘Moderate isolated’ has an HR of 1.009 (0.812, 1.253) with a P value of 9.39 times 10 super minus 1. ‘Most isolated’ has an HR of 1.301 (1.006, 1.683) with a P value of 4.49 times 10 super minus 2. The P trend is 7.09 times 10 super minus 2.

In the Prediabetes section:

* Loneliness: ‘No loneliness’ is the reference. ‘Loneliness’ has an HR of 1.818 (1.463, 2.258) with a P value of 6.55 times 10 super minus 8. The error bar is entirely to the right of the null line.

* Social isolation: ‘Least isolated’ is the reference. ‘Moderate isolated’ has an HR of 1.134 (0.956, 1.344) with a P value of 1.48 times 10 super minus 1. ‘Most isolated’ has an HR of 1.401 (1.141, 1.719) with a P value of 1.25 times 10 super minus 3. The P trend is 1.63 times 10 super minus 3.

The X-axis at the bottom represents HR (95% CI) on a scale from 0 to 2.5.

Navigate back to Figure 3.
