# Supplementary material for: Loneliness and social isolation as risk factors for incident depression in type 2 diabetes mellitus: Evidence from a prospective cohort study
Source: Eur Psychiatry. 2026 Jul 29;69(1):e83. doi: 10.1192/j.eurpsy.2026.12244 (PMC13735759; doi:10.1192/j.eurpsy.2026.12244)
Supplement: Supplementary file 1 [file S0924933826122445sup001.docx]

**Assessment of covariates**

**Supplementary table legends**

**Supplementary Table 1.** Information of UK Biobank cohort about variables used for adjustment in this study.

**Supplementary Table 2.** Missing values of variables in this study.

**Supplementary Table 3.** Baseline characteristics of the study population grouped by different status during follow-up.

**Supplementary Table 4.** Separate and joint effects of loneliness and social isolation on the risk of five transitions (Model 2).

**Supplementary Table 5.** Risk of the five transitions according to groups of social isolation in different category of loneliness and groups of loneliness in different category of social isolation (Model 1).

**Supplementary Table 6.** Risk of the five transitions according to groups of social isolation in different category of loneliness and groups of loneliness in different category of social isolation (Model 2).

**Supplementary Table 7.** The effects of the scores and items of loneliness and social isolation on the risk of five transitions (Model 1).

**Supplementary Table 8.** The effects of the scores and items of loneliness and social isolation on the risk of five transitions (Model 2).

**Supplementary Table 9.** Separate effects of loneliness and social isolation on the risk of five transitions under different glycemic statuses (Model 2).

**Supplementary Table 10.** Joint effects of loneliness and social isolation on the risk of five transitions under different glycemic statuses (Model 2).

**Supplementary Table 11.** Risk of the five transitions according to groups of social isolation in different category of loneliness and groups of loneliness in different category of social isolation under different glycemic statuses (Model 2).

**Supplementary Table 12.** Subgroup analyses for the effects of loneliness on the risk of five transitions.

**Supplementary Table 13.** Subgroup analyses for the effects of social isolation on the risk of five transitions.

**Supplementary Table 14.** Separate and joint effects of loneliness and social isolation on the risk of five transitions after further adjusting for glucose metabolism measures.

**Supplementary Table 15.** Risk of the five transitions according to groups of social isolation in different category of loneliness and groups of loneliness in different category of social isolation after further adjusting for glucose metabolism measures.

**Supplementary Table 16.** Separate and joint effects of loneliness and social isolation on the risk of five transitions after further adjusting for lipid metabolism measures.

**Supplementary Table 17.** Risk of the five transitions according to groups of social isolation in different category of loneliness and groups of loneliness in different category of social isolation after further adjusting for lipid metabolism measures.

**Supplementary Table 18.** Separate effects of loneliness and social isolation on the risk of five transitions after further adjusting for glucose and lipid metabolism measures.

**Supplementary Table 19.** Risk of the five transitions according to groups of social isolation in different category of loneliness and groups of loneliness in different category of social isolation after further adjusting for glucose and lipid metabolism measures.

**Supplementary Table 20.** Separate and joint effects of loneliness and social isolation on the risk of five transitions after excluding those occurring T2DM within the first year (n=426,686, Model 2).

**Supplementary Table 21.** Risk of the five transitions according to groups of social isolation in different category of loneliness and groups of loneliness in different category of social isolation after excluding those occurring T2DM within the first year (n=426,686, Model 2).

**Supplementary Table 22.** Separate and joint association of loneliness and social isolation with the risk of five transitions after further adjusting for neuroticism score.

**Supplementary Table 23.** Leave‑one‑item sensitivity analysis for the social isolation score assessing associations with the risk of five transitions (Model 2).

# Assessment of covariates

Specifically, ethnicity was classified into white and non-white. BMI was categorized as underweight or normal weight (<25 kg/m^2^), overweight (25 to <30 kg/m^2^), or obese (≥30 kg/m^2^). Annual household income was segmented into five levels: less than £18,000, £18,000–£30,999, £31,000–£51,999, £52,000–£100,000, and greater than £100,000. Educational attainment was distinguished as college or above versus other qualifications. Current employment status was divided into employed individuals and others. Smoking and alcohol consumption statuses were classified as never, previous or current. Physical activity was quantified by summing the days engaged in walking or moderate to vigorous activities. Sun exposure time was computed as the average hours spent outdoors during summer (Field ID 1050) and winter (Field ID 1060). A healthy sleep score was determined based on five components: insomnia (Field ID 1200), sleep duration (Field ID 1160), snoring (Field ID 1210), chronotype (Field ID 1180), and daytime sleepiness (Field ID 1220). Responses indicating “never/rarely or sometimes insomnia symptoms”, “sleep 7-8 h per day”, “no self-reported snoring”, “morning person”, and “never/rarely or sometimes daytime sleeping” were assigned 1 point, while 0 points were given if the criteria were not met. The healthy sleep score ranged from 0 to 5, with a score of 4 or higher defining a healthy sleep pattern[1]. Laboratory tests conducted on blood samples included measurements of FBG (mmol/L), HbA1c (%), total cholesterol (TC, mmol/L), triglyceride (TG, mmol/L), low-density lipoprotein cholesterol (LDL-C, mmol/L), high-density lipoprotein cholesterol (HDL-C, mmol/L), and lipoprotein (a) (Lp (a), nmol/L). The history of medical conditions, including cardiovascular disease (CVD), hypertension, and cancer, was ascertained through touchscreen queries, verbal interviews, and hospital records at baseline. Neuroticism score was a summary score of neuroticisms, based on 12 neurotic behaviour domains (Field ID 20127). Detailed information regarding the field IDs and ICD codes for these medical conditions can be found in Supplementary Table 1.

References:

1.	Li, X., et al., *Healthy Sleep Patterns and Risk of Incident Arrhythmias.* J Am Coll Cardiol, 2021. **78**(12): p. 1197-1207.

# Supplementary Table 1. Information of UK Biobank cohort about variables used for adjustment in this study.

| **Variables** | **Field ID** |
| --- | --- |
| **Exposure** |  |
| Social isolation | 709, 1031 and 6160 |
| Loneliness | 2020 and 2110 |
| **Outcome** |  |
| Type 2 diabetes mellitus | 41270 and 41280, ICD-10 E11 |
| Depression | 41270 and 41280, ICD-10 F32 to F33 |
| **Covariates** |  |
| Age | 21003 |
| Sex | 31 |
| Ethnicity | 21000 |
| Body mass index | 21001 |
| Household income | 738 |
| Current employment status | 6142 |
| Education level | 6138 |
| Smoking status | 20116 |
| Drinking status | 20117 |
| Physical activity | 22033 |
| Sleep pattern | 1160, 1180, 1200, 1210 and 1220 |
| Sun exposure time | 1050 and 1060 |
| Cardiovascular disease | 6150, 41270 and 41280, ICD-10 I00-I09, I25-I99 |
| Hypertension | 6150, 41270 and 41280, ICD-10 I10-I15 |
| Cancer | 2453, 41270 and 41280, ICD-10 C00-C97 |
| Fasting blood glucose, FBG | 30740 |
| Glycated haemoglobin, HbA1c | 30750 |
| Cholesterol, TC | 30690 |
| Triglycerides, TG | 30870 |
| Low-density lipoprotein cholesterol, LDL | 30780 |
| High-density lipoprotein cholesterol, HDL | 30760 |
| Lipoprotein A, Lp(a) | 30790 |
| Neuroticism score | 20127 |

# Supplementary Table 2. Missing values of variables in this study.

| **Covariates** | **Missing number** | **Missing percentage** |
| --- | --- | --- |
| Ethnicity | 1,158 | 0.27% |
| Body mass index | 8,829 | 2.06% |
| Household income | 54,984 | 12.86% |
| Current employment status | 3,125 | 0.73% |
| Education level | 70,573 | 16.51% |
| Smoking status | 1,236 | 0.29% |
| Drinking status | 259 | 0.06% |
| Physical activity | 65,643 | 15.35% |
| Sleep pattern | 64,235 | 15.02% |
| Sun exposure time | 24,221 | 5.66% |
| Fasting blood glucose, FBG | 57,688 | 13.49% |
| Glycated haemoglobin, HbA1c | 25,996 | 6.08% |
| Cholesterol, TC | 23,774 | 5.56% |
| Triglycerides, TG | 24,049 | 5.63% |
| Low-density lipoprotein cholesterol, LDL | 24,449 | 5.72% |
| High-density lipoprotein cholesterol, HDL | 57,413 | 13.43% |
| Lipoprotein A, Lp(a) | 102,907 | 24.07% |
| Neuroticism score | 73,266 | 17.13% |

# Supplementary Table 3. Baseline characteristics of the study population grouped by different status during follow-up.

| Variables | All | Incident T2DM | | | Subsequent depression | | | Death | | |
| --- | --- | --- | --- | --- | --- | --- | --- | --- | --- | --- |
|  |  | Yes | No | P value | Yes | No | P value | Yes | No | P value |
| *N* | 427,594 | 14,141 | 413,453 |  | 1,149 | 12,992 |  | 33,024 | 394,570 |  |
| Sex, *n* (%) |  |  |  | < 0.001 |  |  | < 0.001 |  |  | < 0.001 |
| Female | 237,308 (55.5) | 6,170 (43.6) | 231,138 (55.9) |  | 608 (52.9) | 5,562 (42.8) |  | 14,268 (43.2) | 223,040 (56.5) |  |
| Male | 190,286 (44.5) | 7,971 (56.4) | 182,315 (44.1) |  | 541 (47.1) | 7,430 (57.2) |  | 18,756 (56.8) | 171,530 (43.5) |  |
| Age, Median (Q1,Q3) | 57 (50, 63) | 61 (54, 65) | 57 (50, 63) | < 0.001 | 58 (51, 64) | 61 (55, 65) | < 0.001 | 63 (58, 67) | 57 (49, 62) | < 0.001 |
| Social isolation |  |  |  | < 0.001 |  |  | < 0.001 |  |  | < 0.001 |
| Least isolated | 196,010 (45.8) | 5,833 (41.2) | 190,177 (46.0) |  | 402 (35.0) | 5,431 (41.8) |  | 13,421 (40.6) | 182,589 (46.3) |  |
| Moderate isolated | 172,200 (40.3) | 5,919 (41.9) | 166,281 (40.2) |  | 484 (42.1) | 5,435 (41.8) |  | 13,691 (41.5) | 158,509 (40.2) |  |
| Most isolated | 59,384 (13.9) | 2,389 (16.9) | 56,995 (13.8) |  | 263 (22.9) | 2,126 (16.4) |  | 5,912 (17.9) | 53,472 (13.6) |  |
| Loneliness |  |  |  | < 0.001 |  |  | < 0.001 |  |  | < 0.001 |
| No Loneliness | 408,434 (95.5) | 13,168 (93.1) | 395,266 (95.6) |  | 988 (86.0) | 12,180 (93.8) |  | 31,088 (94.1) | 377,346 (95.6) |  |
| Loneliness | 19,160 (4.5) | 973 (6.9) | 18,187 (4.4) |  | 161 (14.0) | 812 (6.2) |  | 1,936 (5.9) | 17,224 (4.4) |  |
| Ethnicity, *n* (%) |  |  |  | < 0.001 |  |  | 0.062 |  |  | < 0.001 |
| White | 409,647 (95.8) | 12,876 (91.1) | 396,771 (96.0) |  | 1,064 (92.6) | 11,812 (90.9) |  | 32,213 (97.5) | 377,434 (95.7) |  |
| Non-white | 17,947 (4.2) | 1,265 (8.9) | 16,682 (4.0) |  | 85 (7.4) | 1,180 (9.1) |  | 811 (2.5) | 17,137 (4.3) |  |
| BMI, Median (Q1,Q3) | 26.6 (24.0, 29.4) | 29.9 (27.0, 33.5) | 26.5 (24.0, 29.2) | < 0.001 | 30.5 (27.1, 34.4) | 29.9 (27.0, 33.5) | 0.001 | 27.0 (24.5, 30.0) | 26.5 (24.0, 29.3) | < 0.001 |
| Household income, *n* (%) |  |  |  | < 0.001 |  |  | < 0.001 |  |  | < 0.001 |
| Low | 311,409 (72.8) | 11,813 (83.5) | 299,596 (72.5) |  | 1,002 (87.2) | 1,0811 (83.2) |  | 27,767 (84.1) | 283,642 (71.9) |  |
| High | 116,185 (27.2) | 2,328 (16.5) | 113,857 (27.5) |  | 147 (12.8) | 2,181 (16.8) |  | 5,257 (15.9) | 110,928 (28.1) |  |
| Current employment status, *n* (%) |  |  |  | < 0.001 |  |  | < 0.001 |  |  | < 0.001 |
| Employed | 255,947 (59.9) | 6,512 (46.1) | 249,435 (60.3) |  | 463 (40.3) | 6,049 (46.6) |  | 11,476 (34.8) | 244,471 (62) |  |
| Others | 171,647 (40.1) | 7,629 (53.9) | 164,018 (39.7) |  | 686 (59.7) | 6,943 (53.4) |  | 21,548 (65.2) | 150,099 (38) |  |
| Education level, *n* (%) |  |  |  | < 0.001 |  |  | 0.804 |  |  | < 0.001 |
| College or above | 172,534 (40.3) | 4,594 (32.5) | 167,940 (40.6) |  | 369 (32.1) | 4,225 (32.5) |  | 12,043 (36.5) | 160,491 (40.7) |  |
| Others | 255,060 (59.7) | 9,547 (67.5) | 245,513 (59.4) |  | 780 (67.9) | 8,767 (67.5) |  | 20,981 (63.5) | 234,079 (59.3) |  |
| Smoking status, *n* (%) |  |  |  | < 0.001 |  |  | < 0.001 |  |  | < 0.001 |
| Never | 237,088 (55.4) | 6,379 (45.1) | 230,709 (55.8) |  | 492 (42.8) | 5,887 (45.3) |  | 13,305 (40.3) | 223,783 (56.7) |  |
| Previous | 146,814 (34.3) | 5,690 (40.2) | 141,124 (34.1) |  | 437 (38.0) | 5,253 (40.4) |  | 13,533 (41.0) | 133,281 (33.8) |  |
| Current | 43,692 (10.2) | 2,072 (14.7) | 41,620 (10.1) |  | 220 (19.1) | 1,852 (14.3) |  | 6,186 (18.7) | 37,506 (9.5) |  |
| Drinking status, *n* (%) |  |  |  | < 0.001 |  |  | < 0.001 |  |  | < 0.001 |
| Never | 16,077 (3.8) | 905 (6.4) | 15,172 (3.7) |  | 70 (6.1) | 835 (6.4) |  | 1,370 (4.1) | 14,707 (3.7) |  |
| Previous | 13,690 (3.2) | 767 (5.4) | 12,923 (3.1) |  | 113 (9.8) | 654 (5.0) |  | 1,795 (5.4) | 11,895 (3.0) |  |
| Current | 397,827 (93.0) | 12,469 (88.2) | 385,358 (93.2) |  | 966 (84.1) | 11,503 (88.5) |  | 29,859 (90.4) | 367,968 (93.3) |  |
| Physical activity, *n* (%) |  |  |  | < 0.001 |  |  | 0.254 |  |  | < 0.001 |
| Low | 213,797 (50.0) | 7,717 (54.6) | 206,080 (49.8) |  | 646 (56.2) | 7,071 (54.4) |  | 17,099 (51.8) | 196,698 (49.9) |  |
| High | 213,797 (50.0) | 6,424 (45.4) | 207,373 (50.2) |  | 503 (43.8) | 5,921 (45.6) |  | 15,925 (48.2) | 197,872 (50.1) |  |
| Sleep pattern, *n* (%) |  |  |  | < 0.001 |  |  | < 0.001 |  |  | < 0.001 |
| Unhealthy | 213,823 (50.0) | 8,700 (61.5) | 205,123 (49.6) |  | 819 (71.3) | 7,881 (60.7) |  | 18,378 (55.7) | 195,445 (49.5) |  |
| Healthy | 213,771 (50.0) | 5,441 (38.5) | 208,330 (50.4) |  | 330 (28.7) | 5,111 (39.3) |  | 14,646 (44.3) | 199,125 (50.5) |  |
| Sun exposure time, Median (Q1,Q3) | 2.5 (1.5, 3.5) | 2.9 (2.0, 4.0) | 2.5 (1.5, 3.5) | < 0.001 | 2.9 (2.0, 4.0) | 2.9 (2.0, 4.0) | 0.344 | 2.9 (2.0, 4.0) | 2.5 (1.5, 3.5) | < 0.001 |
| CVD, *n* (%) |  |  |  | < 0.001 |  |  | < 0.001 |  |  | < 0.001 |
| No | 288,756 (67.5) | 5,123 (36.2) | 283,633 (68.6) |  | 321 (27.9) | 4,802 (37.0) |  | 11,048 (33.5) | 277,708 (70.4) |  |
| Yes | 138,838 (32.5) | 9,018 (63.8) | 129,820 (31.4) |  | 828 (72.1) | 8,190 (63.0) |  | 21,976 (66.5) | 116,862 (29.6) |  |
| Hypertension, *n* (%) |  |  |  | < 0.001 |  |  | 0.004 |  |  | < 0.001 |
| No | 271,077 (63.4) | 3,258 (23.0) | 267,819 (64.8) |  | 225 (19.6) | 3,033 (23.3) |  | 14,863 (45.0) | 256,214 (64.9) |  |
| Yes | 156,517 (36.6) | 10,883 (77.0) | 145,634 (35.2) |  | 924 (80.4) | 9,959 (76.7) |  | 18,161 (55.0) | 138,356 (35.1) |  |
| Cancer, *n* (%) |  |  |  | < 0.001 |  |  | 0.234 |  |  | < 0.001 |
| No | 332,842 (77.8) | 9,331 (66.0) | 323,511 (78.2) |  | 777 (67.6) | 8,554 (65.8) |  | 12,542 (38.0) | 320,300 (81.2) |  |
| Yes | 94,752 (22.2) | 4,810 (34.0) | 89,942 (21.8) |  | 372 (32.4) | 4,438 (34.2) |  | 20,482 (62.0) | 74,270 (18.8) |  |
| FBG, mmol/L | 4.9 (4.6, 5.2) | 5 (4.8, 5.5) | 4.9 (4.6, 5.2) | < 0.001 | 5 (4.8, 5.5) | 5 (4.8, 5.5) | 0.132 | 4.9 (4.7, 5.3) | 4.9 (4.6, 5.2) | < 0.001 |
| HbA1c, % | 5.3 (5.2, 5.5) | 5.7 (5.4, 6) | 5.3 (5.1, 5.5) | < 0.001 | 5.7 (5.4, 6) | 5.7 (5.4, 6) | < 0.001 | 5.4 (5.2, 5.6) | 5.3 (5.1, 5.5) | < 0.001 |
| TC, mmol/L | 5.8 (5.1, 6.4) | 5.6 (4.8, 6.3) | 5.8 (5.1, 6.4) | < 0.001 | 5.7 (4.9, 6.3) | 5.6 (4.8, 6.3) | 0.091 | 5.7 (4.9, 6.3) | 5.8 (5.1, 6.4) | < 0.001 |
| TG, mmol/L | 1.5 (1.1, 2.1) | 1.9 (1.5, 2.8) | 1.5 (1.1, 2) | < 0.001 | 2 (1.5, 2.8) | 1.9 (1.5, 2.8) | 0.02 | 1.6 (1.1, 2.2) | 1.5 (1.1, 2) | < 0.001 |
| LDL, mmol/L | 3.6 (3.1, 4.1) | 3.6 (2.9, 4.1) | 3.6 (3.1, 4.1) | < 0.001 | 3.6 (3, 4.1) | 3.6 (2.9, 4.1) | 0.097 | 3.6 (2.9, 4.1) | 3.6 (3.1, 4.1) | < 0.001 |
| HDL, mmol/L | 1.5 (1.2, 1.6) | 1.3 (1.1, 1.5) | 1.5 (1.2, 1.7) | < 0.001 | 1.3 (1, 1.5) | 1.3 (1.1, 1.5) | 0.802 | 1.4 (1.2, 1.6) | 1.5 (1.2, 1.7) | < 0.001 |
| Lp(a), mmol/L | 41.1 (12.1, 44.9) | 44.7 (12.4, 44.9) | 40.9 (12.1, 44.9) | 0.014 | 44.7 (12, 44.9) | 44.7 (12.4, 44.9) | 0.615 | 43.6 (12.5, 44.9) | 40.9 (12.1, 44.9) | 0.020 |
| Neuroticism score | 4.1 (2, 6) | 4.1 (2, 6) | 4.1 (2, 6) | < 0.001 | 5 (4.1, 8) | 4.1 (2, 6) | < 0.001 | 4.1 (2, 5) | 4.1 (2, 6) | < 0.001 |

Abbreviations: Type 2 diabetes mellitus, T2DM; Cardiovascular disease, CVD; Fasting blood glucose, FBG; Glycated haemoglobin, HbA1c; Cholesterol, TC; Triglycerides, TG; Low-density lipoprotein cholesterol, LDL; High-density lipoprotein cholesterol, HDL; Lipoprotein A, Lp(a).

# Supplementary Table 4. Separate and joint effects of loneliness and social isolation on the risk of five transitions (Model 2).

|  | *N* | Baseline to incident T2DM | | Baseline to death | | Incident T2DM to subsequent depression | | Incident T2DM to death | | Subsequent depression to death | |
| --- | --- | --- | --- | --- | --- | --- | --- | --- | --- | --- | --- |
|  |  | HR(95%CI) | P value | HR(95%CI) | P value | HR(95%CI) | P value | HR(95%CI) | P value | HR(95%CI) | P value |
| **Separate effects** |  |  |  |  |  |  |  |  |  |  |  |
| Loneliness |  |  |  |  |  |  |  |  |  |  |  |
| No loneliness | 408,434 | as reference |  | as reference |  | as reference |  | as reference |  | as reference |  |
| Loneliness | 19,160 | **1.160(1.086,1.239)** | **9.38E-06** | **1.169(1.114,1.227)** | **2.60E-10** | **1.793(1.513,2.124)** | **1.58E-11** | 1.025(0.863,1.217) | 7.82E-01 | 1.193(0.797,1.784) | 3.91E-01 |
| Social isolation |  |  |  |  |  |  |  |  |  |  |  |
| Least isolated | 196,010 | as reference |  | as reference |  | as reference |  | as reference |  | as reference |  |
| Moderate isolated | 172,200 | **1.078(1.039,1.118)** | **6.03E-05** | **1.151(1.123,1.180)** | **2.10E-28** | 1.090(0.954,1.246) | 2.06E-01 | **1.165(1.058,1.283)** | **1.92E-03** | 0.981(0.706,1.361) | 9.07E-01 |
| Most isolated | 59,384 | **1.167(1.112,1.226)** | **5.04E-10** | **1.404(1.359,1.450)** | **3.81E-92** | **1.369(1.167,1.607)** | **1.16E-04** | **1.268(1.118,1.438)** | **2.19E-04** | 1.267(0.876,1.833) | 2.08E-01 |
| P trend |  |  | **1.25E-10** |  | **1.08E-91** |  | **2.39E-04** |  | **6.11E-05** |  | 2.53E-01 |
| **Joint effects** |  |  |  |  |  |  |  |  |  |  |  |
| No loneliness |  |  |  |  |  |  |  |  |  |  |  |
| Least isolated | 191,058 | as reference |  | as reference |  | as reference |  | as reference |  | as reference |  |
| Moderate isolated | 163,986 | **1.075(1.035,1.116)** | **1.59E-04** | **1.147(1.118,1.176)** | **5.00E-26** | 1.079(0.937,1.243) | 2.92E-01 | **1.180(1.069,1.303)** | **1.02E-03** | 0.996(0.704,1.409) | 9.82E-01 |
| Most isolated | 53,390 | **1.156(1.098,1.217)** | **3.96E-08** | **1.397(1.350,1.445)** | **8.35E-82** | **1.316(1.104,1.569)** | **2.20E-03** | **1.270(1.112,1.451)** | **4.24E-04** | 1.271(0.847,1.909) | 2.47E-01 |
| Loneliness |  |  |  |  |  |  |  |  |  |  |  |
| Least isolated | 4,952 | **1.148(1.001,1.316)** | **4.80E-02** | 1.095(0.984,1.219) | 9.77E-02 | **1.983(1.387,2.836)** | **1.74E-04** | 1.141(0.796,1.636) | 4.72E-01 | 1.362(0.581,3.189) | 4.77E-01 |
| Moderate isolated | 8,214 | **1.214(1.097,1.343)** | **1.71E-04** | **1.288(1.195,1.388)** | **3.19E-11** | **1.858(1.421,2.431)** | **6.11E-06** | 1.040(0.790,1.370) | 7.80E-01 | 1.073(0.545,2.109) | 8.39E-01 |
| Most isolated | 5,994 | **1.298(1.164,1.448)** | **2.92E-06** | **1.497(1.385,1.619)** | **3.60E-24** | **2.066(1.574,2.713)** | **1.74E-07** | 1.298(0.985,1.710) | 6.43E-02 | 1.389(0.752,2.565) | 2.94E-01 |
| P trend |  |  | **4.02E-13** |  | **2.67E-73** |  | **1.39E-12** |  | **5.44E-03** |  | 2.03E-01 |

Abbreviations: Type 2 diabetes mellitus, T2DM; Hazard ratio, HR; Confidence interval, CI.

# Supplementary Table 5. Risk of the five transitions according to groups of social isolation in different category of loneliness and groups of loneliness in different category of social isolation (Model 1).

|  | *N* | Baseline to incident T2DM | | Baseline to death | | | | Incident T2DM to subsequent depression | | Incident T2DM to death | | Subsequent depression to death | | |
| --- | --- | --- | --- | --- | --- | --- | --- | --- | --- | --- | --- | --- | --- | --- |
|  |  | HR(95%CI) | P value | HR(95%CI) | | | P value | HR(95%CI) | P value | HR(95%CI) | P value | HR(95%CI) | | P value |
| No loneliness |  |  |  |  | | |  |  |  |  |  |  | |  |
| Least isolated | 191,058 | as reference |  | as reference | | |  | as reference |  | as reference |  | as reference | |  |
| Moderate isolated | 163,986 | **1.089(1.049,1.131)** | **8.45E-06** | **1.146(1.117,1.176)** | | | **8.03E-26** | 1.087(0.943,1.252) | 2.49E-01 | **1.201(1.087,1.326)** | **3.04E-04** | 0.912(0.646,1.285) | | 5.98E-01 |
| Most isolated | 53,390 | **1.165(1.106,1.227)** | **7.06E-09** | **1.393(1.346,1.442)** | | | **1.47E-80** | **1.321(1.107,1.575)** | **2.00E-03** | **1.264(1.106,1.444)** | **5.74E-04** | 1.155(0.769,1.734) | | 4.86E-01 |
| P trend |  |  | **3.47E-10** |  | | | **8.60E-81** |  | **3.25E-03** |  | **6.21E-05** |  | | 6.11E-01 |
| Loneliness |  |  |  |  |  | |  |  |  |  |  |  | |  |
| Least isolated | 4,952 | as reference |  | as reference | |  | | as reference |  | as reference |  | as reference |  | |
| Moderate isolated | 8,214 | 1.074(0.909,1.269) | 3.98E-01 | **1.192(1.048,1.357)** | | | **7.57E-03** | 0.942(0.615,1.441) | 7.82E-01 | 1.032(0.659,1.615) | 8.91E-01 | 0.820(0.271,2.483) | | 7.25E-01 |
| Most isolated | 5,994 | 1.154(0.968,1.376) | 1.10E-01 | **1.382(1.208,1.582)** | | | **2.56E-06** | 1.094(0.708,1.691) | 6.86E-01 | 1.165(0.736,1.845) | 5.14E-01 | 0.840(0.289,2.444) | | 7.49E-01 |
| P trend |  |  | 1.06E-01 |  | | | **1.93E-06** |  | 5.92E-01 |  | 4.82E-01 |  | | 7.86E-01 |
| Least isolated |  |  |  |  | | |  |  |  |  |  |  | |  |
| No loneliness | 191,058 | as reference |  | as reference |  | | | as reference |  | as reference |  | as reference |  | |
| Loneliness | 4,952 | **1.221(1.065,1.401)** | **4.27E-03** | 1.111(0.998,1.237) | | | 5.48E-02 | **1.952(1.362,2.797)** | **2.69E-04** | 1.079(0.752,1.549) | 6.80E-01 | 1.069(0.452,2.529) | | 8.79E-01 |
| Moderate isolated |  |  |  |  | | |  |  |  |  |  |  | |  |
| No loneliness | 164,000 | as reference |  | as reference |  | | | as reference |  | as reference |  | as reference |  | |
| Loneliness | 8,214 | **1.202(1.086,1.330)** | **3.59E-04** | **1.146(1.063,1.235)** | | | **3.67E-04** | **1.769(1.355,2.309)** | **2.76E-05** | 0.891(0.677,1.172) | 4.10E-01 | 1.118(0.562,2.225) | | 7.51E-01 |
| Most isolated |  |  |  |  | | |  |  |  |  |  |  | |  |
| No loneliness | 53,390 | as reference |  | as reference |  | | | as reference |  | as reference |  | as reference |  | |
| Loneliness | 5,994 | **1.199(1.068,1.345)** | **2.03E-03** | 1.074(0.991,1.165) | | | 8.33E-02 | **1.535(1.151,2.047)** | **3.57E-03** | 0.971(0.728,1.296) | 8.43E-01 | 1.091(0.565,2.107) | | 7.96E-01 |
| P interaction |  |  | 0.811 |  | | | 0.876 |  | 0.478 |  | 0.551 |  | | 0.794 |

Abbreviations: Type 2 diabetes mellitus, T2DM; Hazard ratio, HR; Confidence interval, CI.

# Supplementary Table 6. Risk of the five transitions according to groups of social isolation in different category of loneliness and groups of loneliness in different category of social isolation (Model 2).

|  | *N* | Baseline to incident T2DM | | Baseline to death | | Incident T2DM to subsequent depression | | Incident T2DM to death | | Subsequent depression to death | | |
| --- | --- | --- | --- | --- | --- | --- | --- | --- | --- | --- | --- | --- |
|  |  | HR(95%CI) | P value | HR(95%CI) | P value | HR(95%CI) | P value | HR(95%CI) | P value | HR(95%CI) | | P value |
| No loneliness |  |  |  |  |  |  |  |  |  |  | |  |
| Least isolated | 191,058 | as reference |  | as reference |  | as reference |  | as reference |  | as reference | |  |
| Moderate isolated | 163,986 | **1.075(1.035,1.116)** | **1.69E-04** | **1.146(1.117,1.176)** | **8.48E-26** | 1.074(0.933,1.238) | 3.20E-01 | **1.183(1.071,1.306)** | **9.05E-04** | 0.971(0.685,1.375) | | 8.67E-01 |
| Most isolated | 53,390 | **1.155(1.096,1.216)** | **5.01E-08** | **1.395(1.348,1.444)** | **4.23E-81** | **1.304(1.093,1.556)** | **3.17E-03** | **1.272(1.113,1.453)** | **4.04E-04** | 1.262(0.839,1.898) | | 2.64E-01 |
| P trend |  |  | **8.90E-09** |  | **3.73E-81** |  | **5.37E-03** |  | **6.57E-05** |  | | 3.38E-01 |
| Loneliness |  |  |  |  |  |  |  |  |  |  | |  |
| Least isolated | 4,952 | as reference |  | as reference |  | as reference |  | as reference |  | as reference |  | |
| Moderate isolated | 8,214 | 1.064(0.901,1.257) | 4.64E-01 | **1.177(1.034,1.339)** | **1.36E-02** | 0.921(0.602,1.409) | 7.04E-01 | 0.958(0.611,1.502) | 8.51E-01 | 0.457(0.145,1.440) | | 1.81E-01 |
| Most isolated | 5,994 | 1.149(0.964,1.370) | 1.20E-01 | **1.367(1.195,1.565)** | **5.36E-06** | 1.046(0.676,1.618) | 8.41E-01 | 1.202(0.754,1.917) | 4.39E-01 | 0.566(0.187,1.716) | | 3.15E-01 |
| P trend |  |  | 1.12E-01 |  | **3.65E-06** |  | 7.42E-01 |  | 3.69E-01 |  | | 4.71E-01 |
| Least isolated |  |  |  |  |  |  |  |  |  |  | |  |
| No loneliness | 191,058 | as reference |  | as reference |  | as reference |  | as reference |  | as reference |  | |
| Loneliness | 4,952 | **1.151(1.003,1.320)** | **4.46E-02** | 1.102(0.99,1.227) | 7.64E-02 | **1.991(1.389,2.853)** | **1.77E-04** | 1.143(0.797,1.641) | 4.67E-01 | 1.467(0.606,3.550) | | 3.95E-01 |
| Moderate isolated |  |  |  |  |  |  |  |  |  |  | |  |
| No loneliness | 164,000 | as reference |  | as reference |  | as reference |  | as reference |  | as reference |  | |
| Loneliness | 8,214 | **1.127(1.019,1.247)** | **2.01E-02** | **1.12(1.039,1.207)** | **3.09E-03** | **1.777(1.361,2.320)** | **2.36E-05** | 0.864(0.657,1.137) | 2.97E-01 | 1.028(0.514,2.054) | | 9.38E-01 |
| Most isolated |  |  |  |  |  |  |  |  |  |  | |  |
| No loneliness | 53,390 | as reference |  | as reference |  | as reference |  | as reference |  | as reference |  | |
| Loneliness | 5,994 | 1.122(1.000,1.259) | 5.07E-02 | 1.043(0.962,1.132) | 3.07E-01 | **1.498(1.122,2.000)** | **6.12E-03** | 1.031(0.771,1.379) | 8.38E-01 | 1.009(0.519,1.963) | | 9.78E-01 |
| P interaction |  |  | 0.818 |  | 0.874 |  | 0.405 |  | 0.708 |  | | 0.828 |

Abbreviations: Type 2 diabetes mellitus, T2DM; Hazard ratio, HR; Confidence interval, CI.

# Supplementary Table 7. The effects of the scores and items of loneliness and social isolation on the risk of five transitions (Model 1).

|  | *N* | Baseline to incident T2DM | | Baseline to death | | Incident T2DM to subsequent depression | | Incident T2DM to death | | Subsequent depression to death | |
| --- | --- | --- | --- | --- | --- | --- | --- | --- | --- | --- | --- |
|  |  | HR(95%CI) | P value | HR(95%CI) | P value | HR(95%CI) | P value | HR(95%CI) | P value | HR(95%CI) | P value |
| **Loneliness scores** |  |  |  |  |  |  |  |  |  |  |  |
| 0 point | 310,200 | as reference |  | as reference |  | as reference |  | as reference |  | as reference |  |
| 1 point | 98,234 | **1.137(1.095,1.181)** | **2.79E-11** | **1.137(1.108,1.167)** | **3.64E-22** | **1.446(1.270,1.645)** | **2.30E-08** | 1.067(0.967,1.178) | 1.99E-01 | 1.322(0.972,1.798) | 7.49E-02 |
| 2 points | 19,160 | **1.292(1.208,1.381)** | **6.41E-14** | **1.243(1.183,1.305)** | **4.33E-18** | **2.095(1.751,2.505)** | **5.56E-16** | 1.020(0.856,1.215) | 8.26E-01 | 1.284(0.838,1.966) | 2.50E-01 |
| P trend |  |  | **3.41E-20** |  | **2.76E-33** |  | **3.95E-18** |  | 3.64E-01 |  | 1.08E-01 |
| **Items of loneliness** |  |  |  |  |  |  |  |  |  |  |  |
| Feeling lonely |  |  |  |  |  |  |  |  |  |  |  |
| No | 351,789 | as reference |  | as reference |  | as reference |  | as reference |  | as reference |  |
| Yes | 75,805 | **1.226(1.178,1.277)** | **4.88E-23** | **1.174(1.140,1.208)** | **4.36E-27** | **1.819(1.608,2.058)** | **2.18E-21** | 0.952(0.852,1.064) | 3.86E-01 | 1.247(0.930,1.674) | 1.41E-01 |
| Willing to confide |  |  |  |  |  |  |  |  |  |  |  |
| Yes | 366,845 | as reference |  | as reference |  | as reference |  | as reference |  | as reference |  |
| No | 60,749 | **1.090(1.044,1.137)** | **7.50E-05** | **1.116(1.084,1.149)** | **1.35E-13** | **1.279(1.112,1.471)** | **5.56E-04** | **1.129(1.016,1.255)** | **2.42E-02** | 1.182(0.857,1.629) | 3.08E-01 |
| **Social isolation scores** |  |  |  |  |  |  |  |  |  |  |  |
| 0 point | 196,010 | as reference |  | as reference |  | as reference |  | as reference |  | as reference |  |
| 1 point | 172,200 | **1.094(1.055,1.135)** | **1.42E-06** | **1.153(1.124,1.182)** | **6.05E-29** | 1.103(0.965,1.261) | 1.50E-01 | **1.186(1.077,1.307)** | **5.37E-04** | 0.922(0.666,1.276) | 6.25E-01 |
| 2 points | 54,148 | **1.165(1.108,1.226)** | **3.11E-09** | **1.363(1.317,1.410)** | **4.31E-71** | **1.369(1.160,1.617)** | **2.11E-04** | **1.235(1.083,1.408)** | **1.61E-03** | 1.177(0.805,1.722) | 4.01E-01 |
| 3 points | 5,236 | **1.364(1.206,1.542)** | **7.51E-07** | **1.814(1.679,1.960)** | **1.67E-51** | **1.555(1.096,2.206)** | **1.34E-02** | **1.363(1.015,1.831)** | **3.95E-02** | 1.030(0.442,2.399) | 9.45E-01 |
| P trend |  |  | **6.98E-14** |  | **2.52E-102** |  | **6.50E-05** |  | **8.76E-05** |  | 5.35E-01 |
| **Items of social isolation** |  |  |  |  |  |  |  |  |  |  |  |
| Visit friends |  |  |  |  |  |  |  |  |  |  |  |
| More | 335,586 | as reference |  | as reference |  | as reference |  | as reference |  | as reference |  |
| Less | 92,008 | 1.024(0.983,1.067) | 2.55E-01 | **1.135(1.104,1.166)** | **2.56E-19** | 1.098(0.954,1.264) | 1.92E-01 | 1.110(0.999,1.234) | 5.26E-02 | 1.200(0.867,1.660) | 2.72E-01 |
| Social activities |  |  |  |  |  |  |  |  |  |  |  |
| More | 300,378 | as reference |  | as reference |  | as reference |  | as reference |  | as reference |  |
| Less | 127,216 | **1.153(1.113,1.195)** | **2.19E-15** | **1.137(1.110,1.165)** | **3.78E-25** | **1.184(1.049,1.335)** | **6.05E-03** | 1.054(0.962,1.156) | 2.61E-01 | 0.948(0.709,1.266) | 7.16E-01 |
| Living alone |  |  |  |  |  |  |  |  |  |  |  |
| No | 350,614 | as reference |  | as reference |  | as reference |  | as reference |  | as reference |  |
| Yes | 76,980 | **1.080(1.036,1.125)** | **3.12E-04** | **1.296(1.261,1.331)** | **8.34E-79** | **1.222(1.068,1.398)** | **3.49E-03** | **1.233(1.113,1.365)** | **5.55E-05** | 1.083(0.792,1.481) | 6.17E-01 |

Abbreviations: Type 2 diabetes mellitus, T2DM; Hazard ratio, HR; Confidence interval, CI.

# Supplementary Table 8. The effects of the scores and items of loneliness and social isolation on the risk of five transitions (Model 2).

|  | *N* | Baseline to incident T2DM | | Baseline to death | | Incident T2DM to subsequent depression | | Incident T2DM to death | | Subsequent depression to death | |
| --- | --- | --- | --- | --- | --- | --- | --- | --- | --- | --- | --- |
|  |  | HR(95%CI) | P value | HR(95%CI) | P value | HR(95%CI) | P value | HR(95%CI) | P value | HR(95%CI) | P value |
| **Loneliness scores** |  |  |  |  |  |  |  |  |  |  |  |
| 0 point | 310,200 | as reference |  | as reference |  | as reference |  | as reference |  | as reference |  |
| 1 point | 98,234 | **1.107(1.066,1.150)** | **1.28E-07** | **1.129(1.100,1.158)** | **8.10E-20** | **1.427(1.254,1.624)** | **6.92E-08** | 1.077(0.976,1.189) | 1.41E-01 | 1.262(0.926,1.720) | 1.40E-01 |
| 2 points | 19,160 | **1.199(1.122,1.283)** | **1.02E-07** | **1.213(1.155,1.274)** | **1.50E-14** | **2.074(1.734,2.481)** | **1.39E-15** | 1.050(0.881,1.251) | 5.87E-01 | 1.330(0.865,2.044) | 1.94E-01 |
| P trend |  |  | **6.44E-12** |  | **4.25E-28** |  | **1.86E-17** |  | 2.12E-01 |  | 1.08E-01 |
| **Items of loneliness** |  |  |  |  |  |  |  |  |  |  |  |
| Feeling lonely |  |  |  |  |  |  |  |  |  |  |  |
| No | 351,789 | as reference |  | as reference |  | as reference |  | as reference |  | as reference |  |
| Yes | 75,805 | **1.160(1.114,1.208)** | **7.58E-13** | **1.137(1.104,1.171)** | **6.22E-18** | **1.786(1.578,2.021)** | **3.67E-20** | 0.958(0.857,1.070) | 4.45E-01 | 1.231(0.912,1.662) | 1.74E-01 |
| Willing to confide |  |  |  |  |  |  |  |  |  |  |  |
| Yes | 366,845 | as reference |  | as reference |  | as reference |  | as reference |  | as reference |  |
| No | 60,749 | **1.072(1.027,1.118)** | **1.47E-03** | **1.126(1.094,1.159)** | **1.38E-15** | **1.282(1.115,1.475)** | **4.89E-04** | **1.156(1.040,1.285)** | **7.42E-03** | 1.203(0.872,1.658) | 2.60E-01 |
| **Social isolation scores** |  |  |  |  |  |  |  |  |  |  |  |
| 0 point | 196,010 | as reference |  | as reference |  | as reference |  | as reference |  | as reference |  |
| 1 point | 172,200 | **1.078(1.039,1.118)** | **5.68E-05** | **1.152(1.123,1.181)** | **1.24E-28** | 1.090(0.954,1.247) | 2.04E-01 | **1.166(1.058,1.284)** | **1.88E-03** | 0.981(0.707,1.361) | 9.08E-01 |
| 2 points | 54,148 | **1.151(1.094,1.211)** | **5.11E-08** | **1.362(1.316,1.409)** | **1.38E-70** | **1.344(1.138,1.587)** | **5.01E-04** | **1.245(1.092,1.420)** | **1.06E-03** | 1.283(0.875,1.881) | 2.03E-01 |
| 3 points | 5,236 | **1.317(1.164,1.489)** | **1.15E-05** | **1.806(1.672,1.951)** | **9.23E-51** | **1.575(1.110,2.234)** | **1.09E-02** | **1.458(1.086,1.959)** | **1.21E-02** | 1.163(0.498,2.717) | 7.27E-01 |
| P trend |  |  | **2.02E-11** |  | **2.88E-101** |  | **1.25E-04** |  | **3.95E-05** |  | 2.66E-01 |
| **Items of social isolation** |  |  |  |  |  |  |  |  |  |  |  |
| Visit friends |  |  |  |  |  |  |  |  |  |  |  |
| More | 335,586 | as reference |  | as reference |  | as reference |  | as reference |  | as reference |  |
| Less | 92,008 | 1.032(0.990,1.075) | 1.37E-01 | **1.148(1.117,1.181)** | **7.00E-23** | 1.091(0.948,1.255) | 2.24E-01 | 1.097(0.987,1.219) | 8.75E-02 | 1.205(0.869,1.669) | 2.63E-01 |
| Social activities |  |  |  |  |  |  |  |  |  |  |  |
| More | 300,378 | as reference |  | as reference |  | as reference |  | as reference |  | as reference |  |
| Less | 127,216 | **1.130(1.091,1.170)** | **1.19E-11** | **1.121(1.094,1.149)** | **2.82E-20** | **1.176(1.043,1.327)** | **8.33E-03** | 1.075(0.980,1.178) | 1.25E-01 | 1.008(0.754,1.349) | 9.55E-01 |
| Living alone |  |  |  |  |  |  |  |  |  |  |  |
| No | 350,614 | as reference |  | as reference |  | as reference |  | as reference |  | as reference |  |
| Yes | 76,980 | **1.068(1.024,1.113)** | **2.01E-03** | **1.300(1.265,1.335)** | **1.34E-80** | **1.214(1.061,1.388)** | **4.76E-03** | **1.236(1.116,1.369)** | **4.54E-05** | 1.160(0.845,1.592) | 3.58E-01 |

Abbreviations: Type 2 diabetes mellitus, T2DM; Hazard ratio, HR; Confidence interval, CI.

# Supplementary Table 9. Separate effects of loneliness and social isolation on the risk of five transitions under different glycemic statuses (Model 2).

|  | | *N* | Baseline to incident T2DM | | Baseline to death |  | Incident T2DM to subsequent depression | | Incident T2DM to death | | Subsequent depression to death | |
| --- | --- | --- | --- | --- | --- | --- | --- | --- | --- | --- | --- | --- |
|  | |  | HR(95%CI) | P value | HR(95%CI) | P value | HR(95%CI) | P value | HR(95%CI) | P value | HR(95%CI) | P value |
| **NGT** | |  |  |  |  |  |  |  |  |  |  |  |
| Loneliness | |  |  |  |  |  |  |  |  |  |  |  |
| No loneliness | | 312,017 | as reference |  | as reference |  | as reference |  | as reference |  | as reference |  |
| Loneliness | | 14,161 | **1.172(1.048,1.310)** | **5.31E-03** | **1.200(1.132,1.272)** | **1.03E-09** | **1.771(1.346,2.330)** | **4.52E-05** | 0.943(0.696,1.278) | 7.05E-01 | 1.591(0.839,3.017) | 1.55E-01 |
| Social isolation | |  |  |  |  |  |  |  |  |  |  |  |
| Least isolated | | 151,185 | as reference |  | as reference |  | as reference |  | as reference |  | as reference |  |
| Moderate isolated | | 130,774 | **1.067(1.003,1.135)** | **3.91E-02** | **1.156(1.122,1.191)** | **1.35E-21** | 1.009(0.812,1.253) | 9.39E-01 | 1.124(0.956,1.320) | 1.56E-01 | 0.682(0.393,1.181) | 1.72E-01 |
| Most isolated | | 44,219 | **1.205(1.110,1.308)** | **9.01E-06** | **1.410(1.355,1.467)** | **4.10E-65** | **1.301(1.006,1.683)** | **4.49E-02** | **1.276(1.034,1.576)** | **2.34E-02** | 1.226(0.687,2.187) | 4.90E-01 |
| P trend | |  |  | **1.51E-05** |  | **1.35E-65** |  | 7.09E-02 |  | **2.04E-02** |  | 6.33E-01 |
| **Prediabetes** | |  |  |  |  |  |  |  |  |  |  |  |
| Loneliness |  |  |  |  |  |  |  |  |  |  |  |  |
| No loneliness | | 96,417 | as reference |  | as reference |  | as reference |  | as reference |  | as reference |  |
| Loneliness | | 4,999 | **1.119(1.031,1.214)** | **6.89E-03** | **1.101(1.010,1.201)** | **2.92E-02** | **1.818(1.463,2.258)** | **6.55E-08** | 1.069(0.867,1.318) | 5.30E-01 | 1.075(0.628,1.842) | 7.91E-01 |
| Social isolation |  | |  |  |  |  |  |  |  |  |  |  |
| Least isolated | | 44,825 | as reference |  | as reference |  | as reference |  | as reference |  | as reference |  |
| Moderate isolated | | 41,426 | **1.060(1.013,1.109)** | **1.12E-02** | **1.132(1.082,1.185)** | **8.35E-08** | 1.134(0.956,1.344) | 1.48E-01 | **1.191(1.055,1.344)** | **4.75E-03** | 1.193(0.779,1.826) | 4.16E-01 |
| Most isolated | | 15,165 | **1.092(1.028,1.160)** | **4.29E-03** | **1.375(1.297,1.457)** | **6.76E-27** | **1.401(1.141,1.719)** | **1.25E-03** | **1.270(1.085,1.486)** | **2.87E-03** | 1.260(0.761,2.088) | 3.69E-01 |
| P trend | |  |  | **1.50E-03** |  | **4.29E-26** |  | **1.63E-03** |  | **9.15E-04** |  | 3.45E-01 |

Abbreviations: Type 2 diabetes mellitus, T2DM; Hazard ratio, HR; Confidence interval, CI; Normal glucose tolerance, NGT.

# Supplementary Table 10. Joint effects of loneliness and social isolation on the risk of five transitions under different glycemic statuses (Model 2).

|  | *N* | Baseline to incident T2DM | | Baseline to death | | Incident T2DM to subsequent depression | | Incident T2DM to death | | Subsequent depression to death | |
| --- | --- | --- | --- | --- | --- | --- | --- | --- | --- | --- | --- |
|  |  | HR(95%CI) | P value | HR(95%CI) | P value | HR(95%CI) | P value | HR(95%CI) | P value | HR(95%CI) | P value |
| **NGT** |  |  |  |  |  |  |  |  |  |  |  |
| No loneliness |  |  |  |  |  |  |  |  |  |  |  |
| Least isolated | 147,447 | as reference |  | as reference |  | as reference |  | as reference |  | as reference |  |
| Moderate isolated | 124,688 | 1.061(0.995,1.130) | 6.99E-02 | **1.149(1.114,1.184)** | **4.60E-19** | 0.966(0.768,1.215) | 7.66E-01 | 1.146(0.972,1.352) | 1.05E-01 | 0.586(0.321,1.072) | 8.27E-02 |
| Most isolated | 39,882 | **1.208(1.107,1.317)** | **1.99E-05** | **1.387(1.331,1.446)** | **4.91E-54** | 1.248(0.942,1.652) | 1.22E-01 | **1.316(1.057,1.639)** | **1.41E-02** | 1.164(0.621,2.182) | 6.35E-01 |
| Loneliness |  |  |  |  |  |  |  |  |  |  |  |
| Least isolated | 3,738 | 1.166(0.929,1.465) | 1.86E-01 | 1.023(0.896,1.168) | 7.34E-01 | 1.658(0.895,3.071) | 1.08E-01 | 1.242(0.696,2.216) | 4.64E-01 | 1.108(0.247,4.963) | 8.94E-01 |
| Moderate isolated | 6,086 | **1.277(1.079,1.511)** | **4.42E-03** | **1.326(1.212,1.451)** | **7.71E-10** | **1.910(1.253,2.911)** | **2.64E-03** | 0.955(0.605,1.507) | 8.42E-01 | 1.396(0.548,3.557) | 4.84E-01 |
| Most isolated | 4,337 | **1.248(1.031,1.511)** | **2.32E-02** | **1.608(1.464,1.766)** | **2.63E-23** | **1.830(1.174,2.853)** | **7.67E-03** | 1.060(0.604,1.860) | 8.39E-01 | 1.552(0.564,4.271) | 3.95E-01 |
| P trend |  |  | **1.98E-06** |  | **2.02E-56** |  | **5.67E-05** |  | 2.09E-01 |  | 1.82E-01 |
| **Prediabetes** |  |  |  |  |  |  |  |  |  |  |  |
| No loneliness |  |  |  |  |  |  |  |  |  |  |  |
| Least isolated | 43,611 | as reference |  | as reference |  | as reference |  | as reference |  | as reference |  |
| Moderate isolated | 39,298 | **1.061(1.013,1.111)** | **1.28E-02** | **1.136(1.085,1.19)** | **7.30E-08** | 1.144(0.955,1.369) | 1.44E-01 | **1.202(1.062,1.361)** | **3.61E-03** | 1.230(0.787,1.920) | 3.64E-01 |
| Most isolated | 13,508 | **1.075(1.008,1.146)** | **2.77E-02** | **1.400(1.318,1.488)** | **1.19E-27** | **1.348(1.075,1.689)** | **9.64E-03** | **1.246(1.054,1.474)** | **1.01E-02** | 1.278(0.727,2.244) | 3.94E-01 |
| Loneliness |  |  |  |  |  |  |  |  |  |  |  |
| Least isolated | 1,214 | 1.108(0.934,1.315) | 2.38E-01 | **1.254(1.045,1.506)** | **1.52E-02** | **2.222(1.430,3.452)** | **3.81E-04** | 1.076(0.679,1.705) | 7.54E-01 | 1.269(0.439,3.667) | 6.60E-01 |
| Moderate isolated | 2,128 | 1.125(0.991,1.276) | 6.80E-02 | **1.205(1.053,1.379)** | **6.63E-03** | **1.837(1.295,2.606)** | **6.55E-04** | 1.082(0.765,1.528) | 6.57E-01 | 1.120(0.389,3.231) | 8.33E-01 |
| Most isolated | 1,657 | **1.245(1.089,1.422)** | **1.30E-03** | **1.274(1.106,1.467)** | **7.76E-04** | **2.199(1.556,3.107)** | **8.03E-06** | **1.428(1.038,1.965)** | **2.84E-02** | 1.321(0.594,2.936) | 4.95E-01 |
| P trend |  |  | **8.64E-05** |  | **7.99E-18** |  | **6.42E-09** |  | **1.05E-02** |  | 4.72E-01 |

Abbreviations: Type 2 diabetes mellitus, T2DM; Hazard ratio, HR; Confidence interval, CI; Normal glucose tolerance, NGT.

# Supplementary Table 11. Risk of the five transitions according to groups of social isolation in different category of loneliness and groups of loneliness in different category of social isolation under different glycemic statuses (Model 2).

|  | *N* | | Baseline to incident T2DM | | Baseline to death | | Incident T2DM to subsequent depression | | Incident T2DM to death | | Subsequent depression to death | |
| --- | --- | --- | --- | --- | --- | --- | --- | --- | --- | --- | --- | --- |
|  |  |  | HR(95%CI) | P value | HR(95%CI) | P value | HR(95%CI) | P value | HR(95%CI) | P value | HR(95%CI) | P value |
| **NGT** |  | |  |  |  |  |  |  |  |  |  |  |
| No loneliness |  | |  |  |  |  |  |  |  |  |  |  |
| Least isolated | 147,447 | | as reference |  | as reference |  | as reference |  | as reference |  | as reference |  |
| Moderate isolated | 124,688 | | 1.059(0.994,1.128) | 7.79E-02 | **1.148(1.114,1.184)** | **5.79E-19** | 0.969(0.770,1.219) | 7.86E-01 | 1.143(0.969,1.349) | 1.12E-01 | 0.582(0.316,1.072) | 8.24E-02 |
| Most isolated | 39,882 | | **1.203(1.103,1.312)** | **3.04E-05** | **1.387(1.331,1.446)** | **7.93E-54** | 1.249(0.942,1.656) | 1.23E-01 | **1.308(1.050,1.629)** | **1.67E-02** | 1.171(0.621,2.207) | 6.26E-01 |
| P trend |  | |  | **6.59E-05** |  | **6.29E-55** |  | 2.06E-01 |  | **1.30E-02** |  | 8.54E-01 |
| Loneliness |  | |  |  |  |  |  |  |  |  |  |  |
| Least isolated | 3,738 | | as reference |  | as reference |  | as reference |  | as reference |  |  |  |
| Moderate isolated | 6,086 | | 1.126(0.853,1.488) | 4.02E-01 | **1.288(1.099,1.509)** | **1.73E-03** | 1.079(0.517,2.255) | 8.39E-01 | 0.735(0.350,1.544) | 4.16E-01 |  |  |
| Most isolated | 4,337 | | 1.131(0.840,1.523) | 4.16E-01 | **1.555(1.319,1.833)** | **1.41E-07** | 0.944(0.434,2.052) | 8.84E-01 | 0.882(0.381,2.042) | 7.69E-01 |  |  |
| P trend |  | |  | 4.45E-01 |  | **1.06E-07** |  | 8.14E-01 |  | 7.75E-01 |  |  |
| **Prediabetes** |  | |  |  |  |  |  |  |  |  |  |  |
| No loneliness |  | |  |  |  |  |  |  |  |  |  |  |
| Least isolated | 43,611 | | as reference |  | as reference |  | as reference |  | as reference |  | as reference |  |
| Moderate isolated | 39,298 | | **1.061(1.013,1.111)** | **1.29E-02** | **1.135(1.083,1.189)** | **9.80E-08** | 1.139(0.951,1.363) | 1.58E-01 | **1.207(1.066,1.366)** | **3.01E-03** | 1.212(0.774,1.898) | 4.01E-01 |
| Most isolated | 13,508 | | **1.075(1.008,1.146)** | **2.83E-02** | **1.397(1.314,1.484)** | **3.77E-27** | **1.333(1.062,1.672)** | **1.30E-02** | **1.253(1.059,1.482)** | **8.60E-03** | 1.289(0.729,2.277) | 3.83E-01 |
| P trend |  | |  | **7.86E-03** |  | **3.57E-26** |  | **1.27E-02** |  | **1.75E-03** |  | 3.41E-01 |
| Loneliness |  |  |  |  |  |  |  |  |  |  |  |  |
| Least isolated | 1,214 | | as reference |  | as reference |  | as reference |  | as reference |  | as reference |  |
| Moderate isolated | 2,128 | | 1.010(0.820,1.244) | 9.29E-01 | 0.978(0.781,1.223) | 8.43E-01 | 0.86(0.505,1.466) | 5.80E-01 | 1.115(0.622,1.999) | 7.14E-01 | 2.297(0.295,17.860) | 4.27E-01 |
| Most isolated | 1,657 | | 1.112(0.894,1.384) | 3.41E-01 | 1.043(0.824,1.321) | 7.25E-01 | 1.057(0.612,1.824) | 8.43E-01 | 1.422(0.789,2.565) | 2.42E-01 | 1.082(0.230,5.092) | 9.20E-01 |
| P trend |  | |  | 2.96E-01 |  | 6.63E-01 |  | 7.38E-01 |  | 2.04E-01 |  | 8.96E-01 |
| **NGT** |  |  |  |  |  |  |  |  |  |  |  |  |
| Least isolated |  | |  |  |  |  |  |  |  |  |  |  |
| No loneliness | 147,447 | | as reference |  | as reference |  | as reference |  | as reference |  | as reference |  |
| Loneliness | 3,738 | | 1.177(0.937,1.480) | 1.62E-01 | 1.032(0.904,1.178) | 6.46E-01 | 1.628(0.872,3.039) | 1.26E-01 | 1.265(0.707,2.265) | 4.28E-01 | 1.014(0.198,5.181) | 9.87E-01 |
| Moderate isolated |  | |  |  |  |  |  |  |  |  |  |  |
| No loneliness | 124,688 | | as reference |  | as reference |  | as reference |  | as reference |  | as reference |  |
| Loneliness | 6,086 | | **1.193(1.008,1.413)** | **4.01E-02** | **1.149(1.050,1.257)** | **2.49E-03** | **2.068(1.356,3.156)** | **7.47E-04** | 0.845(0.535,1.334) | 4.70E-01 | 1.704(0.542,5.352) | 3.62E-01 |
| Most isolated |  | |  |  |  |  |  |  |  |  |  |  |
| No loneliness | 39,882 | | as reference |  | as reference |  | as reference |  | as reference |  | as reference |  |
| Loneliness | 4,337 | | 1.027(0.841,1.255) | 7.93E-01 | **1.123(1.018,1.238)** | **2.04E-02** | 1.418(0.883,2.276) | 1.48E-01 | 0.815(0.454,1.462) | 4.92E-01 | 1.287(0.392,4.230) | 6.78E-01 |
| P interaction |  | |  | 0.447 |  | 0.120 |  | 0.769 |  | 0.323 |  | 0.790 |
| **Prediabetes** |  | |  |  |  |  |  |  |  |  |  |  |
| Least isolated |  | |  |  |  |  |  |  |  |  |  |  |
| No loneliness | 43,611 | | as reference |  | as reference |  | as reference |  | as reference |  | as reference |  |
| Loneliness | 1,214 | | 1.110(0.935,1.318) | 2.32E-01 | **1.253(1.043,1.505)** | **1.61E-02** | **2.239(1.437,3.491)** | **3.72E-04** | 1.071(0.674,1.701) | 7.71E-01 | 1.433(0.480,4.279) | 5.19E-01 |
| Moderate isolated |  | |  |  |  |  |  |  |  |  |  |  |
| No loneliness | 39,298 | | as reference |  | as reference |  | as reference |  | as reference |  | as reference |  |
| Loneliness | 2,128 | | 1.061(0.935,1.204) | 3.57E-01 | 1.061(0.927,1.214) | 3.88E-01 | **1.610(1.137,2.280)** | **7.30E-03** | 0.879(0.623,1.242) | 4.65E-01 | 1.049(0.361,3.048) | 9.30E-01 |
| Most isolated |  | |  |  |  |  |  |  |  |  |  |  |
| No loneliness | 13,508 | | as reference |  | as reference |  | as reference |  | as reference |  | as reference |  |
| Loneliness | 1,657 | | **1.158(1.006,1.334)** | **4.13E-02** | 0.895(0.773,1.036) | 1.38E-01 | **1.551(1.070,2.247)** | **2.04E-02** | 1.184(0.841,1.668) | 3.33E-01 | 1.167(0.463,2.942) | 7.43E-01 |
| P interaction |  | |  | 0.693 |  | 0.012 |  | 0.380 |  | 0.780 |  | 0.772 |

Abbreviations: Type 2 diabetes mellitus, T2DM; Hazard ratio, HR; Confidence interval, CI; Normal glucose tolerance, NGT.

# Supplementary Table 12. Subgroup analyses for the effects of loneliness on the risk of five transitions.

|  |  | *N* | Baseline to incident T2DM | | | Baseline to death | | | Incident T2DM to subsequent depression | | | Incident T2DM to death | | | Subsequent depression to death | | |
| --- | --- | --- | --- | --- | --- | --- | --- | --- | --- | --- | --- | --- | --- | --- | --- | --- | --- |
|  |  |  | HR(95%CI) | P value | P interaction | HR(95%CI) | P value | P interaction | HR(95%CI) | P value | P interaction | HR(95%CI) | P value | P interaction | HR(95%CI) | P value | P interaction |
| Sex |  |  |  |  | 0.910 |  |  | 0.263 |  |  | 0.925 |  |  | 0.126 |  |  | 0.525 |
| Female | No loneliness | 227,657 | as reference |  |  | as reference |  |  | as reference |  |  | as reference |  |  | as reference |  |  |
|  | Loneliness | 9,651 | **1.181(1.069,1.306)** | **1.13E-03** |  | **1.140(1.058,1.229)** | **6.00E-04** |  | **1.807(1.420,2.300)** | **1.52E-06** |  | 1.224(0.932,1.608) | 1.46E-01 |  | 0.984(0.526,1.841) | 9.59E-01 |  |
| Male | No loneliness | 180,777 | as reference |  |  | as reference |  |  | as reference |  |  | as reference |  |  | as reference |  |  |
|  | Loneliness | 9,509 | **1.137(1.042,1.240)** | **3.99E-03** |  | **1.174(1.102,1.251)** | **7.81E-07** |  | **1.756(1.381,2.232)** | **4.28E-06** |  | 0.908(0.727,1.134) | 3.93E-01 |  | 1.292(0.754,2.214) | 3.51E-01 |  |
| Age |  |  |  |  | 0.199 |  |  | 0.058 |  |  | 0.102 |  |  | 0.458 |  |  | 0.619 |
| ≤65 years | No loneliness | 351,231 | as reference |  |  | as reference |  |  | as reference |  |  | as reference |  |  | as reference |  |  |
|  | Loneliness | 16,839 | **1.156(1.075,1.243)** | **8.38E-05** |  | **1.084(1.023,1.149)** | **5.98E-03** |  | **2.023(1.692,2.419)** | **1.16E-14** |  | 1.036(0.844,1.271) | 7.36E-01 |  | 1.039(0.67,1.612) | 8.64E-01 |  |
| >65 years | No loneliness | 57,203 | as reference |  |  | as reference |  |  | as reference |  |  | as reference |  |  | as reference |  |  |
|  | Loneliness | 2,321 | 1.094(0.934,1.283) | 2.66E-01 |  | **1.231(1.125,1.346)** | **5.56E-06** |  | 1.191(0.689,2.058) | 5.32E-01 |  | 0.898(0.652,1.236) | 5.10E-01 |  | 1.274(0.442,3.672) | 6.54E-01 |  |
| Ethnicity |  |  |  |  | 0.151 |  |  | 0.975 |  |  | 0.240 |  |  | 0.853 |  |  | 0.989 |
| White | No loneliness | 391,533 | as reference |  |  | as reference |  |  | as reference |  |  | as reference |  |  | as reference |  |  |
|  | Loneliness | 18,114 | **1.170(1.092,1.253)** | **8.36E-06** |  | **1.168(1.111,1.226)** | **7.08E-10** |  | **1.744(1.459,2.085)** | **9.82E-10** |  | 1.033(0.866,1.232) | 7.21E-01 |  | 1.210(0.795,1.841) | 3.74E-01 |  |
| Non-white | No loneliness | 16,901 | as reference |  |  | as reference |  |  | as reference |  |  | as reference |  |  | as reference |  |  |
|  | Loneliness | 1,046 | 1.045(0.840,1.299) | 6.94E-01 |  | 1.218(0.920,1.612) | 1.68E-01 |  | **2.268(1.288,3.992)** | **4.55E-03** |  | 0.823(0.374,1.814) | 6.29E-01 |  | 2.085(0.261,16.674) | 4.88E-01 |  |
| Body mass index |  |  |  |  | 0.322 |  |  | 0.099 |  |  | 0.112 |  |  | 0.381 |  |  | 0.630 |
| Normal | No loneliness | 138,253 | as reference |  |  | as reference |  |  | as reference |  |  | as reference |  |  | as reference |  |  |
|  | Loneliness | 5,512 | 1.041(0.818,1.324) | 7.47E-01 |  | **1.177(1.075,1.289)** | **4.45E-04** |  | 1.148(0.526,2.504) | 7.29E-01 |  | 0.854(0.473,1.541) | 6.00E-01 |  | #NUM! | 9.99E-01 |  |
| Over weight | No loneliness | 183,186 | as reference |  |  | as reference |  |  | as reference |  |  | as reference |  |  | as reference |  |  |
|  | Loneliness | 8,326 | **1.144(1.025,1.276)** | **1.61E-02** |  | **1.197(1.114,1.287)** | **1.03E-06** |  | **1.623(1.203,2.189)** | **1.53E-03** |  | 1.032(0.784,1.360) | 8.21E-01 |  | 1.108(0.587,2.091) | 7.52E-01 |  |
| Obesity | No loneliness | 86,995 | as reference |  |  | as reference |  |  | as reference |  |  | as reference |  |  | as reference |  |  |
|  | Loneliness | 5,322 | **1.198(1.098,1.308)** | **5.26E-05** |  | **1.116(1.016,1.227)** | **2.23E-02** |  | **1.976(1.594,2.451)** | **5.56E-10** |  | 1.097(0.863,1.393) | 4.50E-01 |  | 1.333(0.764,2.329) | 3.12E-01 |  |
| Household income |  |  |  |  | 0.557 |  |  | 0.971 |  |  | 0.986 |  |  | 0.294 |  |  | 0.788 |
| Low | No loneliness | 295,659 | as reference |  |  | as reference |  |  | as reference |  |  | as reference |  |  | as reference |  |  |
|  | Loneliness | 15,750 | **1.195(1.114,1.281)** | **5.27E-07** |  | **1.197(1.137,1.260)** | **6.95E-12** |  | **1.839(1.54,2.195)** | **1.63E-11** |  | 1.059(0.885,1.266) | 5.32E-01 |  | 1.167(0.762,1.788) | 4.78E-01 |  |
| High | No loneliness | 112,775 | as reference |  |  | as reference |  |  | as reference |  |  | as reference |  |  | as reference |  |  |
|  | Loneliness | 3,410 | 1.080(0.884,1.320) | 4.52E-01 |  | **1.218(1.053,1.408)** | **7.99E-03** |  | 1.778(0.995,3.176) | 5.21E-02 |  | 0.734(0.390,1.383) | 3.39E-01 |  | 2.909(0.602,14.052) | 1.84E-01 |  |
| Employment status |  |  |  |  | 0.569 |  |  | **0.001** |  |  | 0.712 |  |  | 0.375 |  |  | 0.871 |
| Employed | No loneliness | 245,081 | as reference |  |  | as reference |  |  | as reference |  |  | as reference |  |  | as reference |  |  |
|  | Loneliness | 10,866 | **1.116(1.011,1.233)** | **3.02E-02** |  | 1.049(0.960,1.145) | 2.91E-01 |  | **1.743(1.313,2.313)** | **1.20E-04** |  | 1.180(0.877,1.588) | 2.75E-01 |  | 1.207(0.573,2.545) | 6.21E-01 |  |
| Others | No loneliness | 163,353 | as reference |  |  | as reference |  |  | as reference |  |  | as reference |  |  | as reference |  |  |
|  | Loneliness | 8,294 | **1.185(1.085,1.294)** | **1.58E-04** |  | **1.217(1.148,1.290)** | **3.94E-11** |  | **1.805(1.459,2.234)** | **5.47E-08** |  | 0.933(0.755,1.153) | 5.22E-01 |  | 1.234(0.754,2.020) | 4.03E-01 |  |
| Education level |  |  |  |  | 0.101 |  |  | 0.977 |  |  | 0.475 |  |  | 0.246 |  |  | 0.472 |
| College or above | No loneliness | 165,962 | as reference |  |  | as reference |  |  | as reference |  |  | as reference |  |  | as reference |  |  |
|  | Loneliness | 6,572 | **1.252(1.112,1.408)** | **1.91E-04** |  | **1.167(1.072,1.271)** | **3.56E-04** |  | **1.549(1.127,2.129)** | **7.00E-03** |  | 0.893(0.656,1.217) | 4.74E-01 |  | 1.500(0.742,3.033) | 2.59E-01 |  |
| Others | No loneliness | 242,472 | as reference |  |  | as reference |  |  | as reference |  |  | as reference |  |  | as reference |  |  |
|  | Loneliness | 12,588 | **1.123(1.037,1.215)** | **4.25E-03** |  | **1.169(1.102,1.240)** | **2.34E-07** |  | **1.917(1.568,2.344)** | **2.29E-10** |  | 1.096(0.891,1.349) | 3.87E-01 |  | 1.173(0.710,1.939) | 5.34E-01 |  |
| Smoking status |  |  |  |  | 0.599 |  |  | 0.018 |  |  | 0.072 |  |  | 0.601 |  |  | 0.804 |
| Never | No loneliness | 227,696 | as reference |  |  | as reference |  |  | as reference |  |  | as reference |  |  | as reference |  |  |
|  | Loneliness | 9,392 | **1.168(1.053,1.295)** | **3.36E-03** |  | **1.137(1.044,1.239)** | **3.09E-03** |  | **2.141(1.649,2.78)** | **1.10E-08** |  | 1.115(0.821,1.515) | 4.86E-01 |  | 1.301(0.622,2.725) | 4.85E-01 |  |
| Smoker | No loneliness | 180,738 | as reference |  |  | as reference |  |  | as reference |  |  | as reference |  |  | as reference |  |  |
|  | Loneliness | 9,768 | **1.173(1.077,1.277)** | **2.37E-04** |  | **1.228(1.157,1.302)** | **9.18E-12** |  | **1.587(1.27,1.983)** | **4.83E-05** |  | 1.010(0.820,1.243) | 9.26E-01 |  | 1.168(0.717,1.900) | 5.33E-01 |  |
| Drinking status |  |  |  |  | 0.012 |  |  | 0.111 |  |  | 0.684 |  |  | 0.281 |  |  | 0.551 |
| Never | No loneliness | 15,178 | as reference |  |  | as reference |  |  | as reference |  |  | as reference |  |  | as reference |  |  |
|  | Loneliness | 899 | 0.887(0.685,1.149) | 3.65E-01 |  | 1.041(0.830,1.305) | 7.29E-01 |  | 1.923(0.975,3.791) | 5.91E-02 |  | 1.303(0.667,2.544) | 4.39E-01 |  | 1.473(0.071,30.53) | 8.02E-01 |  |
| Drinker | No loneliness | 393,256 | as reference |  |  | as reference |  |  | as reference |  |  | as reference |  |  | as reference |  |  |
|  | Loneliness | 18,261 | **1.194(1.116,1.278)** | **3.18E-07** |  | **1.185(1.127,1.245)** | **2.15E-11** |  | **1.780(1.492,2.123)** | **1.47E-10** |  | 1.005(0.841,1.202) | 9.54E-01 |  | 1.148(0.757,1.739) | 5.16E-01 |  |
| Physical activity |  |  |  |  | 0.118 |  |  | 0.171 |  |  | 0.551 |  |  | 0.573 |  |  | 0.645 |
| Low | No loneliness | 203,427 | as reference |  |  | as reference |  |  | as reference |  |  | as reference |  |  | as reference |  |  |
|  | Loneliness | 10,370 | **1.121(1.028,1.222)** | **9.94E-03** |  | **1.132(1.060,1.209)** | **2.13E-04** |  | **1.921(1.542,2.394)** | **5.70E-09** |  | 1.059(0.844,1.328) | 6.20E-01 |  | 1.273(0.746,2.172) | 3.76E-01 |  |
| High | No loneliness | 205,007 | as reference |  |  | as reference |  |  | as reference |  |  | as reference |  |  | as reference |  |  |
|  | Loneliness | 8,790 | **1.230(1.112,1.361)** | **5.69E-05** |  | **1.225(1.140,1.316)** | **3.31E-08** |  | **1.635(1.251,2.136)** | **3.19E-04** |  | 0.971(0.744,1.266) | 8.26E-01 |  | 1.118(0.595,2.098) | 7.29E-01 |  |
| Sleep pattern |  |  |  |  | 0.798 |  |  | 0.220 |  |  | 0.744 |  |  | 0.076 |  |  | 0.840 |
| Unhealthy | No loneliness | 201,068 | as reference |  |  | as reference |  |  | as reference |  |  | as reference |  |  | as reference |  |  |
|  | Loneliness | 12,755 | **1.147(1.063,1.238)** | **4.28E-04** |  | **1.124(1.060,1.193)** | **9.38E-05** |  | **1.834(1.519,2.215)** | **2.87E-10** |  | 1.122(0.923,1.364) | 2.49E-01 |  | 1.231(0.787,1.924) | 3.62E-01 |  |
| Healthy | No loneliness | 207,366 | as reference |  |  | as reference |  |  | as reference |  |  | as reference |  |  | as reference |  |  |
|  | Loneliness | 6,405 | **1.176(1.033,1338)** | **1.43E-02** |  | **1.235(1.133,1.345)** | **1.42E-06** |  | **1.600(1.076,2.380)** | **2.03E-02** |  | 0.777(0.535,1.129) | 1.85E-01 |  | 1.086(0.382,3.086) | 8.77E-01 |  |
| Sun exposure time |  |  |  |  | 0.174 |  |  | 0.165 |  |  | 0.082 |  |  | 0.927 |  |  | 0.390 |
| Low | No loneliness | 173,761 | as reference |  |  | as reference |  |  | as reference |  |  | as reference |  |  | as reference |  |  |
|  | Loneliness | 8,314 | **1.232(1.109,1.368)** | **9.74E-05** |  | **1.112(1.025,1.206)** | **1.07E-02** |  | **1.495(1.123,1.991)** | **5.90E-03** |  | 1.021(0.763,1.366) | 8.91E-01 |  | 0.881(0.386,2.011) | 7.64E-01 |  |
| High | No loneliness | 234,673 | as reference |  |  | as reference |  |  | as reference |  |  | as reference |  |  | as reference |  |  |
|  | Loneliness | 10,846 | **1.117(1.026,1.216)** | **1.03E-02** |  | **1.200(1.130,1.275)** | **3.40E-09** |  | **1.989(1.610,2.456)** | **1.77E-10** |  | 1.021(0.825,1.265) | 8.46E-01 |  | 1.363(0.849,2.190) | 2.00E-01 |  |
| CVD |  |  |  |  | 0.159 |  |  | 0.387 |  |  | 0.512 |  |  | 0.219 |  |  | 0.445 |
| No | No loneliness | 276,953 | as reference |  |  | as reference |  |  | as reference |  |  | as reference |  |  | as reference |  |  |
|  | Loneliness | 11,803 | **1.228(1.096,1.375)** | **4.01E-04** |  | **1.202(1.102,1.311)** | **3.46E-05** |  | **1.674(1.194,2.348)** | **2.83E-03** |  | 0.784(0.479,1.283) | 3.34E-01 |  | 0.800(0.173,3.693) | 7.75E-01 |  |
| Yes | No loneliness | 131,481 | as reference |  |  | as reference |  |  | as reference |  |  | as reference |  |  | as reference |  |  |
|  | Loneliness | 7,357 | **1.134(1.046,1.229)** | **2.27E-03** |  | **1.150(1.085,1.219)** | **2.71E-06** |  | **1.847(1.517,2.249)** | **9.80E-10** |  | 1.074(0.894,1.291) | 4.46E-01 |  | 1.220(0.801,1.857) | 3.55E-01 |  |
| Hypertension |  |  |  |  | 0.998 |  |  | 0.229 |  |  | 0.700 |  |  | 0.718 |  |  | 0.293 |
| No | No loneliness | 260,087 | as reference |  |  | as reference |  |  | as reference |  |  | as reference |  |  | as reference |  |  |
|  | Loneliness | 10,990 | 1.133(0.977,1.313) | 9.89E-02 |  | **1.210(1.124,1.303)** | **3.86E-07** |  | **1.644(1.081,2.502)** | **2.02E-02** |  | 1.143(0.759,1.719) | 5.22E-01 |  | 2.282(0.875,5.949) | 9.15E-02 |  |
| Yes | No loneliness | 148,347 | as reference |  |  | as reference |  |  | as reference |  |  | as reference |  |  | as reference |  |  |
|  | Loneliness | 8,170 | **1.164(1.082,1.253)** | **4.84E-05** |  | **1.135(1.064,1.211)** | **1.16E-04** |  | **1.821(1.512,2.193)** | **2.69E-10** |  | 0.997(0.824,1.206) | 9.76E-01 |  | 1.097(0.697,1.726) | 6.90E-01 |  |
| Cancer |  |  |  |  | 0.561 |  |  | **<0.001** |  |  | 0.389 |  |  | 0.388 |  |  | 0.325 |
| No | No loneliness | 317,727 | as reference |  |  | as reference |  |  | as reference |  |  | as reference |  |  | as reference |  |  |
|  | Loneliness | 15,115 | **1.159(1.071,1.254)** | **2.57E-04** |  | **1.313(1.223,1.411)** | **8.99E-14** |  | **1.729(1.406,2.126)** | **2.08E-07** |  | 1.078(0.829,1.401) | 5.77E-01 |  | 1.010(0.542,1.883) | 9.75E-01 |  |
| Yes | No loneliness | 90,707 | as reference |  |  | as reference |  |  | as reference |  |  | as reference |  |  | as reference | 3.36E-01 |  |
|  | Loneliness | 4,045 | **1.163(1.033,1.309)** | **1.26E-02** |  | 1.058(0.991,1.131) | 9.15E-02 |  | **1.937(1.436,2.613)** | **1.50E-05** |  | 0.971(0.773,1.220) | 8.03E-01 |  | 1.303(0.760,2.234) | 3.36E-01 |  |

Abbreviations: Type 2 diabetes mellitus, T2DM; Hazard ratio, HR; Confidence interval, CI; Cardiovascular disease, CVD.

# Supplementary Table 13. Subgroup analyses for the effects of social isolation on the risk of five transitions.

|  |  | *N* | Baseline to incident T2DM | | | Baseline to death | | | Incident T2DM to subsequent depression | | | Incident T2DM to death | | | Subsequent depression to death | | |
| --- | --- | --- | --- | --- | --- | --- | --- | --- | --- | --- | --- | --- | --- | --- | --- | --- | --- |
|  |  |  | HR(95%CI) | P value | P interaction | HR(95%CI) | P value | P interaction | HR(95%CI) | P value | P interaction | HR(95%CI) | P value | P interaction | HR(95%CI) | P value | P interaction |
| Sex |  |  |  |  | 0.543 |  |  | **<0.001** |  |  | 0.262 |  |  | 0.094 |  |  | 0.100 |
| Female | Least isolated | 110,785 | as reference |  |  | as reference |  |  | as reference |  |  | as reference |  |  | as reference |  |  |
|  | Moderate isolated | 96,355 | **1.094(1.035,1.156)** | **1.42E-03** |  | **1.139(1.097,1.183)** | **1.08E-11** |  | 1.094(0.912,1.312) | 3.35E-01 |  | 1.105(0.940,1.300) | 2.27E-01 |  | 1.575(0.951,2.609) | 7.76E-02 |  |
|  | Most isolated | 30,168 | **1.170(1.085,1.261)** | **4.49E-05** |  | **1.298(1.233,1.366)** | **1.62E-23** |  | **1.276(1.016,1.603)** | **3.60E-02** |  | 1.087(0.873,1.353) | 4.57E-01 |  | 1.862(1.056,3.283) | 3.16E-02 |  |
| Male | Least isolated | 85,225 | as reference |  |  | as reference |  |  | as reference |  |  | as reference |  |  | as reference |  |  |
|  | Moderate isolated | 75,845 | **1.066(1.015,1.119)** | **1.04E-02** |  | **1.159(1.121,1.199)** | **4.17E-18** |  | 1.067(0.876,1.300) | 5.20E-01 |  | **1.196(1.060,1.350)** | **3.61E-03** |  | 0.691(0.440,1.086) | 1.09E-01 |  |
|  | Most isolated | 29,216 | **1.169(1.096,1.246)** | **1.84E-06** |  | **1.470(1.408,1.533)** | **3.81E-70** |  | **1.445(1.153,1.810)** | **1.41E-03** |  | **1.365(1.169,1.593)** | **7.90E-05** |  | 1.017(0.611,1.694) | 9.48E-01 |  |
| Age |  |  |  |  | 0.054 |  |  | 0.907 |  |  | 0.399 |  |  | 0.591 |  |  | 0.864 |
| ≤65 years | Least isolated | 167,203 | as reference |  |  | as reference |  |  | as reference |  |  | as reference |  |  | as reference |  |  |
|  | Moderate isolated | 148,529 | **1.075(1.031,1.121)** | **7.26E-04** |  | **1.136(1.102,1.171)** | **4.58E-16** |  | 1.154(0.995,1.339) | 5.81E-02 |  | **1.222(1.08,1.382)** | **1.45E-03** |  | 0.964(0.643,1.446) | 8.59E-01 |  |
|  | Most isolated | 52,338 | **1.180(1.118,1.246)** | **2.34E-09** |  | **1.377(1.324,1.432)** | **5.54E-57** |  | **1.477(1.241,1.757)** | **1.10E-05** |  | **1.207(1.033,1.41)** | **1.78E-02** |  | 1.210(0.775,1.887) | 4.02E-01 |  |
| >65 years | Least isolated | 28,807 | as reference |  |  | as reference |  |  | as reference |  |  | as reference |  |  | as reference |  |  |
|  | Moderate isolated | 23,671 | 1.070(0.993,1.154) | 7.65E-02 |  | **1.141(1.094,1.191)** | **1.22E-09** |  | 0.939(0.686,1.284) | 6.92E-01 |  | 1.095(0.936,1.281) | 2.56E-01 |  | 0.838(0.464,1.517) | 5.60E-01 |  |
|  | Most isolated | 7,046 | 1.073(0.960,1.199) | 2.17E-01 |  | **1.367(1.289,1.450)** | **2.09E-25** |  | 1.219(0.801,1.856) | 3.54E-01 |  | **1.387(1.117,1.722)** | **3.08E-03** |  | 1.381(0.653,2.922) | 3.98E-01 |  |
| Ethnicity |  |  |  |  | **<0.001** |  |  | 0.968 |  |  | 0.269 |  |  | 0.619 |  |  | 0.783 |
| White | Least isolated | 189,265 | as reference |  |  | as reference |  |  | as reference |  |  | as reference |  |  | as reference |  |  |
|  | Moderate isolated | 164,553 | **1.092(1.051,1.135)** | **6.17E-06** |  | **1.150(1.121,1.179)** | **1.85E-27** |  | 1.090(0.949,1.251) | 2.25E-01 |  | **1.175(1.064,1.297)** | **1.39E-03** |  | 0.956(0.683,1.338) | 7.92E-01 |  |
|  | Most isolated | 55,829 | **1.201(1.141,1.264)** | **2.83E-12** |  | **1.399(1.354,1.447)** | **5.58E-88** |  | **1.339(1.133,1.583)** | **6.22E-04** |  | **1.277(1.122,1.453)** | **2.04E-04** |  | 1.259(0.860,1.843) | 2.37E-01 |  |
| Non-white | Least isolated | 6,745 | as reference |  |  | as reference |  |  | as reference |  |  | as reference |  |  | as reference |  |  |
|  | Moderate isolated | 7,647 | 0.920(0.813,1.042) | 1.89E-01 |  | 1.162(0.976,1.384) | 9.18E-02 |  | 0.974(0.576,1.648) | 9.22E-01 |  | 0.868(0.545,1.384) | 5.52E-01 |  | 1.883(0.230,15.443) | 5.56E-01 |  |
|  | Most isolated | 3,555 | 0.905(0.775,1.056) | 2.04E-01 |  | **1.480(1.213,1.805)** | **1.09E-04** |  | 1.671(0.952,2.933) | 7.35E-02 |  | 1.019(0.566,1.836) | 9.50E-01 |  | 1.266(0.180,8.894) | 8.12E-01 |  |
| Body mass index |  |  |  |  | 0.421 |  |  | **0.003** |  |  | 0.817 |  |  | 0.291 |  |  | 0.649 |
| Normal | Least isolated | 65,489 | as reference |  |  | as reference |  |  | as reference |  |  | as reference |  |  | as reference |  |  |
|  | Moderate isolated | 58,001 | **1.137(1.010,1.280)** | **3.37E-02** |  | **1.166(1.114,1.221)** | **7.05E-11** |  | 1.180(0.750,1.857) | 4.73E-01 |  | 1.159(0.861,1.560) | 3.31E-01 |  | 0.353(0.093,1.332) | 1.24E-01 |  |
|  | Most isolated | 20,275 | 1.124(0.960,1.317) | 1.47E-01 |  | **1.452(1.370,1.539)** | **4.83E-36** |  | 1.379(0.792,2.400) | 2.56E-01 |  | **1.634(1.139,2.342)** | **7.59E-03** |  | 0.354(0.065,1.937) | 2.31E-01 |  |
| Over weight | Least isolated | 90,312 | as reference |  |  | as reference |  |  | as reference |  |  | as reference |  |  | as reference |  |  |
|  | Moderate isolated | 76,027 | **1.106(1.044,1.171)** | **5.84E-04** |  | **1.123(1.083,1.165)** | **4.31E-10** |  | 1.177(0.946,1.464) | 1.43E-01 |  | 1.118(0.965,1.294) | 1.37E-01 |  | 0.901(0.534,1.520) | 6.97E-01 |  |
|  | Most isolated | 25,173 | **1.216(1.125,1.313)** | **7.52E-07** |  | **1.374(1.308,1.443)** | **5.50E-37** |  | **1.403(1.075,1.832)** | **1.28E-02** |  | **1.207(0.999,1.458)** | **5.18E-02** |  | **1.754(1.017,3.025)** | **4.32E-02** |  |
| Obesity | Least isolated | 40,209 | as reference |  |  | as reference |  |  | as reference |  |  | as reference |  |  | as reference |  |  |
|  | Moderate isolated | 38,172 | **1.064(1.011,1.121)** | **1.82E-02** |  | **1.178(1.119,1.240)** | **3.05E-10** |  | 1.013(0.844,1.216) | 8.90E-01 |  | **1.199(1.039,1.384)** | **1.28E-02** |  | 1.160(0.715,1.882) | 5.47E-01 |  |
|  | Most isolated | 13,936 | **1.169(1.092,1.252)** | **7.26E-06** |  | **1.358(1.270,1.453)** | **3.90E-19** |  | **1.334(1.076,1.654)** | **8.57E-03** |  | **1.222(1.007,1.482)** | **4.19E-02** |  | 1.033(0.568,1.879) | 9.14E-01 |  |
| Household income |  |  |  |  | 0.157 |  |  | **0.003** |  |  | 0.443 |  |  | 0.318 |  |  | 0.290 |
| Low | Least isolated | 136,586 | as reference |  |  | as reference |  |  | as reference |  |  | as reference |  |  | as reference |  |  |
|  | Moderate isolated | 128,383 | **1.110(1.067,1.155)** | **3.07E-07** |  | **1.194(1.162,1.227)** | **3.30E-37** |  | 1.092(0.946,1.260) | 2.28E-01 |  | **1.183(1.065,1.313)** | **1.68E-03** |  | 0.955(0.674,1.353) | 7.94E-01 |  |
|  | Most isolated | 46,440 | **1.216(1.154,1.282)** | **2.24E-13** |  | **1.474(1.423,1.526)** | **7.55E-105** |  | **1.386(1.170,1.640)** | **1.53E-04** |  | **1.297(1.135,1.483)** | **1.36E-04** |  | 1.339(0.912,1.968) | 1.37E-01 |  |
| High | Least isolated | 59,424 | as reference |  |  | as reference |  |  | as reference |  |  | as reference |  |  | as reference |  |  |
|  | Moderate isolated | 43,817 | 1.026(0.939,1.120) | 5.75E-01 |  | **1.078(1.015,1.146)** | **1.50E-02** |  | 1.215(0.843,1.752) | 2.97E-01 |  | 1.052(0.818,1.352) | 6.95E-01 |  | 2.126(0.640,7.065) | 2.18E-01 |  |
|  | Most isolated | 12,944 | 1.127(0.992,1.281) | 6.67E-02 |  | **1.370(1.257,1.493)** | **8.89E-13** |  | **1.592(1.005,2.523)** | **4.75E-02** |  | 1.072(0.741,1.551) | 7.13E-01 |  | 0.338(0.039,2.926) | 3.24E-01 |  |
| Employment status |  |  |  |  | 0.094 |  |  | **<0.001** |  |  | 0.054 |  |  | 0.384 |  |  | 0.303 |
| Employed | Least isolated | 112,480 | as reference |  |  | as reference |  |  | as reference |  |  | as reference |  |  | as reference |  |  |
|  | Moderate isolated | 105,412 | 1.048(0.993,1.106) | 8.66E-02 |  | **1.124(1.078,1.173)** | **5.67E-08** |  | 1.091(0.887,1.341) | 4.10E-01 |  | 1.068(0.899,1.269) | 4.55E-01 |  | 0.630(0.340,1.166) | 1.41E-01 |  |
|  | Most isolated | 38,055 | **1.115(1.039,1.197)** | **2.50E-03** |  | **1.279(1.211,1.350)** | **6.23E-19** |  | 1.106(0.852,1.436) | 4.48E-01 |  | 1.239(0.999,1.536) | 5.08E-02 |  | 1.105(0.532,2.296) | 7.89E-01 |  |
| Others | Least isolated | 83,530 | as reference |  |  | as reference |  |  | as reference |  |  | as reference |  |  | as reference |  |  |
|  | Moderate isolated | 66,788 | **1.104(1.050,1.160)** | **9.65E-05** |  | **1.163(1.128,1.200)** | **9.08E-22** |  | 1.077(0.903,1.283) | 4.10E-01 |  | **1.228(1.093,1.380)** | **5.64E-04** |  | 1.108(0.748,1.643) | 6.08E-01 |  |
|  | Most isolated | 21,329 | **1.215(1.136,1.300)** | **1.61E-08** |  | **1.470(1.411,1.532)** | **5.45E-76** |  | **1.548(1.263,1.899)** | **2.65E-05** |  | **1.255(1.074,1.467)** | **4.25E-03** |  | 1.391(0.897,2.157) | 1.41E-01 |  |
| Education level |  |  |  |  | 0.902 |  |  | 0.093 |  |  | 0.127 |  |  | 0.338 |  |  | 0.774 |
| College or above | Least isolated | 77,752 | as reference |  |  | as reference |  |  | as reference |  |  | as reference |  |  | as reference |  |  |
|  | Moderate isolated | 69,614 | **1.074(1.007,1.145)** | **3.06E-02** |  | **1.133(1.088,1.181)** | **2.44E-09** |  | **1.288(1.010,1.642)** | **4.16E-02** |  | **1.219(1.028,1.446)** | **2.25E-02** |  | 1.013(0.528,1.942) | 9.70E-01 |  |
|  | Most isolated | 25,168 | **1.161(1.067,1.263)** | **5.44E-04** |  | **1.343(1.273,1.418)** | **6.77E-27** |  | **1.486(1.119,1.975)** | **6.28E-03** |  | **1.410(1.138,1.746)** | **1.68E-03** |  | 1.400(0.696,2.819) | 3.45E-01 |  |
| Others | Least isolated | 118,258 | as reference |  |  | as reference |  |  | as reference |  |  | as reference |  |  | as reference |  |  |
|  | Moderate isolated | 102,586 | **1.081(1.034,1.130)** | **5.47E-04** |  | **1.161(1.126,1.198)** | **7.78E-21** |  | 1.013(0.862,1.189) | 8.77E-01 |  | **1.139(1.013,1.281)** | **3.01E-02** |  | 0.956(0.647,1.411) | 8.19E-01 |  |
|  | Most isolated | 34,216 | **1.175(1.107,1.247)** | **1.28E-07** |  | **1.439(1.381,1.499)** | **1.89E-67** |  | **1.320(1.087,1.603)** | **5.09E-03** |  | **1.198(1.025,1.400)** | **2.33E-02** |  | 1.293(0.815,2.052) | 2.75E-01 |  |
| Smoking status |  |  |  |  | 0.232 |  |  | **<0.001** |  |  | 0.380 |  |  | 0.524 |  |  | **0.023** |
| Never | Least isolated | 111,979 | as reference |  |  | as reference |  |  | as reference |  |  | as reference |  |  | as reference |  |  |
|  | Moderate isolated | 94,620 | **1.067(1.011,1.126)** | **1.87E-02** |  | **1.116(1.074,1.160)** | **1.95E-08** |  | **1.278(1.045,1.564)** | **1.69E-02** |  | 1.127(0.961,1.321) | 1.40E-01 |  | 0.808(0.479,1.364) | 4.25E-01 |  |
|  | Most isolated | 30,489 | **1.165(1.082,1.254)** | **5.19E-05** |  | **1.293(1.225,1.364)** | **5.74E-21** |  | **1.493(1.161,1.920)** | **1.77E-03** |  | 1.231(0.997,1.519) | 5.33E-02 |  | 0.724(0.355,1.478) | 3.75E-01 |  |
| Smoker | Least isolated | 84,031 | as reference |  |  | as reference |  |  | as reference |  |  | as reference |  |  | as reference |  |  |
|  | Moderate isolated | 77,580 | **1.098(1.045,1.154)** | **2.25E-04** |  | **1.208(1.169,1.248)** | **1.75E-29** |  | 0.961(0.803,1.149) | 6.61E-01 |  | **1.222(1.082,1.381)** | **1.23E-03** |  | 1.076(0.699,1.658) | 7.38E-01 |  |
|  | Most isolated | 28,895 | **1.202(1.126,1.283)** | **2.81E-08** |  | **1.553(1.490,1.619)** | **8.44E-97** |  | **1.280(1.041,1.576)** | **1.95E-02** |  | **1.342(1.147,1.570)** | **2.44E-04** |  | **1.708(1.084,2.693)** | **2.10E-02** |  |
| Drinking status |  |  |  |  | **0.044** |  |  | 0.569 |  |  | 0.680 |  |  | 0.749 |  |  |  |
| Never | Least isolated | 6,453 | as reference |  |  | as reference |  |  | as reference |  |  | as reference |  |  |  |  |  |
|  | Moderate isolated | 6,706 | 1.011(0.873,1.171) | 8.84E-01 |  | **1.226(1.076,1.397)** | **2.23E-03** |  | 1.051(0.600,1.841) | 8.62E-01 |  | 1.105(0.717,1.704) | 6.50E-01 |  |  |  |  |
|  | Most isolated | 2,918 | 0.992(0.824,1.194) | 9.32E-01 |  | **1.549(1.329,1.805)** | **2.02E-08** |  | 1.165(0.609,2.228) | 6.44E-01 |  | 1.139(0.665,1.950) | 6.36E-01 |  |  |  |  |
| Drinker | Least isolated | 189,557 | as reference |  |  | as reference |  |  | as reference |  |  | as reference |  |  | as reference |  |  |
|  | Moderate isolated | 165,494 | **1.085(1.045,1.127)** | **2.33E-05** |  | **1.152(1.123,1.182)** | **9.37E-28** |  | 1.091(0.951,1.253) | 2.13E-01 |  | **1.169(1.058,1.290)** | **2.07E-03** |  | 0.879(0.627,1.233) | 4.55E-01 |  |
|  | Most isolated | 56,466 | **1.192(1.133,1.254)** | **9.22E-12** |  | **1.412(1.365,1.460)** | **4.94E-91** |  | **1.383(1.172,1.631)** | **1.20E-04** |  | **1.282(1.127,1.460)** | **1.67E-04** |  | 1.236(0.848,1.801) | 2.71E-01 |  |
| Physical activity |  |  |  |  | 0.379 |  |  | **0.043** |  |  | 0.640 |  |  | 0.182 |  |  | 0.933 |
| Low | Least isolated | 89,921 | as reference |  |  | as reference |  |  | as reference |  |  | as reference |  |  | as reference |  |  |
|  | Moderate isolated | 89,499 | **1.107(1.053,1.164)** | **6.45E-05** |  | **1.123(1.084,1.163)** | **8.95E-11** |  | 1.053(0.878,1.263) | 5.77E-01 |  | **1.203(1.054,1.374)** | **6.25E-03** |  | 0.976(0.615,1.550) | 9.19E-01 |  |
|  | Most isolated | 34,377 | **1.140(1.068,1.216)** | **7.33E-05** |  | **1.370(1.311,1.432)** | **1.76E-44** |  | **1.445(1.173,1.778)** | **5.24E-04** |  | **1.369(1.161,1.614)** | **1.87E-04** |  | 1.274(0.768,2.113) | 3.48E-01 |  |
| High | Least isolated | 106,089 | as reference |  |  | as reference |  |  | as reference |  |  | as reference |  |  | as reference |  |  |
|  | Moderate isolated | 82,701 | **1.048(0.993,1.106)** | **8.70E-02** |  | **1.186(1.145,1.229)** | **2.68E-21** |  | 1.136(0.933,1.385) | 2.05E-01 |  | 1.128(0.979,1.299) | 9.61E-02 |  | 0.930(0.578,1.497) | 7.64E-01 |  |
|  | Most isolated | 25,007 | **1.230(1.142,1.324)** | **4.69E-08** |  | **1.462(1.393,1.535)** | **4.40E-53** |  | 1.257(0.977,1.617) | 7.53E-02 |  | 1.133(0.930,1.380) | 2.17E-01 |  | 1.414(0.812,2.463) | 2.21E-01 |  |
| Sleep pattern |  |  |  |  | 0.559 |  |  | 0.122 |  |  | 0.675 |  |  | 0.250 |  |  | 0.298 |
| Unhealthy | Least isolated | 92,335 | as reference |  |  | as reference |  |  | as reference |  |  | as reference |  |  | as reference |  |  |
|  | Moderate isolated | 87,818 | **1.066(1.017,1.117)** | **7.62E-03** |  | **1.155(1.116,1.195)** | **1.03E-16** |  | 1.081(0.921,1.269) | 3.38E-01 |  | 1.113(0.983,1.259) | 9.12E-02 |  | 1.121(0.750,1.678) | 5.77E-01 |  |
|  | Most isolated | 33,670 | **1.145(1.078,1.217)** | **1.23E-05** |  | **1.404(1.345,1.466)** | **2.11E-54** |  | **1.352(1.121,1.631)** | **1.64E-03** |  | **1.212(1.034,1.421)** | **1.74E-02** |  | 1.376(0.888,2.132) | 1.53E-01 |  |
| Healthy | Least isolated | 103,675 | as reference |  |  | as reference |  |  | as reference |  |  | as reference |  |  | as reference |  |  |
|  | Moderate isolated | 84,382 | **1.081(1.020,1.145)** | **8.83E-03** |  | **1.143(1.102,1.186)** | **8.29E-13** |  | 1.074(0.842,1.370) | 5.65E-02 |  | **1.253(1.073,1.463)** | **4.32E-03** |  | 0.763(0.422,1.380) | 3.71E-01 |  |
|  | Most isolated | 25,714 | **1.170(1.078,1.270)** | **1.81E-04** |  | **1.389(1.320,1.462)** | **9.92E-37** |  | 1.323(0.973,1.800) | 7.47E-02 |  | **1.365(1.109,1.680)** | **3.30E-03** |  | 1.025(0.486,2.165) | 9.48E-01 |  |
| Sun exposure time |  |  |  |  | 0.153 |  |  | 0.348 |  |  | **0.023** |  |  | 0.643 |  |  | 0.644 |
| Low | Least isolated | 79,191 | as reference |  |  | as reference |  |  | as reference |  |  | as reference |  |  | as reference |  |  |
|  | Moderate isolated | 74,775 | 1.043(0.980,1.111) | 1.83E-01 |  | **1.150(1.102,1.201)** | **1.35E-10** |  | 0.939(0.752,1.172) | 5.78E-01 |  | 1.149(0.964,1.369) | 1.22E-01 |  | 1.247(0.692,2.248) | 4.63E-01 |  |
|  | Most isolated | 28,109 | **1.129(1.042,1.222)** | **2.84E-03** |  | **1.363(1.291,1.438)** | **1.64E-29** |  | 1.089(0.836,1.419) | 5.26E-01 |  | 1.185(0.951,1.476) | 1.30E-01 |  | 1.045(0.515,2.122) | 9.02E-01 |  |
| High | Least isolated | 116,819 | as reference |  |  | as reference |  |  | as reference |  |  | as reference |  |  | as reference |  |  |
|  | Moderate isolated | 97,425 | **1.096(1.048,1.147)** | **6.71E-05** |  | **1.150(1.115,1.186)** | **5.16E-19** |  | 1.178(0.996,1.393) | 5.60E-02 |  | **1.172(1.043,1.316)** | **7.43E-03** |  | 0.897(0.599,1.343) | 5.97E-01 |  |
|  | Most isolated | 31,275 | **1.191(1.120,1.267)** | **2.97E-08** |  | **1.423(1.365,1.482)** | **4.76E-63** |  | **1.529(1.250,1.869)** | **3.48E-05** |  | **1.315(1.127,1.534)** | **4.90E-04** |  | 1.229(0.786,1.922) | 3.66E-01 |  |
| CVD |  |  |  |  | 0.058 |  |  | 0.130 |  |  | 0.469 |  |  | 0.941 |  |  | 0.978 |
| No | Least isolated | 133,073 | as reference |  |  | as reference |  |  | as reference |  |  | as reference |  |  | as reference |  |  |
|  | Moderate isolated | 116,228 | 1.042(0.981,1.106) | 1.85E-01 |  | **1.177(1.128,1.228)** | **3.69E-14** |  | 1.054(0.821,1.352) | 6.79E-01 |  | 1.017(0.807,1.283) | 8.85E-01 |  | 0.623(0.192,2.016) | 4.29E-01 |  |
|  | Most isolated | 39,455 | **1.086(1.001,1.179)** | **4.82E-02** |  | **1.467(1.387,1.551)** | **2.28E-41** |  | 1.286(0.945,1.752) | 1.10E-01 |  | 1.294(0.963,1.738) | 8.68E-02 |  | 2.419(0.702,8.338) | 1.62E-01 |  |
| Yes | Least isolated | 62,937 | as reference |  |  | as reference |  |  | as reference |  |  | as reference |  |  | as reference |  |  |
|  | Moderate isolated | 55,972 | **1.100(1.050,1.151)** | **5.09E-05** |  | **1.137(1.103,1.173)** | **3.31E-16** |  | 1.106(0.943,1.296) | 2.15E-01 |  | **1.196(1.075,1.33)** | **9.75E-04** |  | 1.032(0.729,1.460) | 8.60E-01 |  |
|  | Most isolated | 19,929 | **1.213(1.142,1.289)** | **4.20E-10** |  | **1.378(1.323,1.434)** | **9.90E-55** |  | **1.412(1.170,1.703)** | **3.13E-04** |  | **1.261(1.097,1.45)** | **1.08E-03** |  | 1.241(0.836,1.843) | 2.84E-01 |  |
| Hypertension |  |  |  |  | 0.778 |  |  | 0.084 |  |  | 0.406 |  |  | 0.175 |  |  | 0.361 |
| No | Least isolated | 125,750 | as reference |  |  | as reference |  |  | as reference |  |  | as reference |  |  | as reference |  |  |
|  | Moderate isolated | 108,547 | 1.036(0.960,1.118) | 3.65E-01 |  | **1.134(1.093,1.176)** | **1.27E-11** |  | 0.960(0.712,1.295) | 7.91E-01 |  | 0.939(0.763,1.156) | 5.54E-01 |  | 0.901(0.392,2.070) | 8.05E-01 |  |
|  | Most isolated | 36,780 | **1.155(1.044,1.278)** | **5.24E-03** |  | **1.362(1.297,1.429)** | **5.27E-36** |  | 1.262(0.881,1.806) | 2.04E-01 |  | 1.230(0.940,1.610) | 1.32E-01 |  | 1.298(0.492,3.426) | 5.99E-01 |  |
| Yes | Least isolated | 70,260 | as reference |  |  | as reference |  |  | as reference |  |  | as reference |  |  | as reference |  |  |
|  | Moderate isolated | 63,653 | **1.091(1.046,1.137)** | **4.33E-05** |  | **1.165(1.126,1.206)** | **2.86E-18** |  | 1.123(0.966,1.304) | 1.30E-01 |  | **1.236(1.108,1.379)** | **1.52E-04** |  | 0.976(0.679,1.402) | 8.94E-01 |  |
|  | Most isolated | 22,604 | **1.166(1.103,1.232)** | **6.65E-08** |  | **1.437(1.374,1.502)** | **1.30E-57** |  | **1.394(1.166,1.668)** | **2.73E-04** |  | **1.296(1.123,1.494)** | **3.76E-04** |  | 1.336(0.890,2.004) | 1.62E-01 |  |
| Cancer |  |  |  |  | 0.994 |  |  | **<0.001** |  |  | 0.085 |  |  | 0.259 |  |  | 0.656 |
| No | Least isolated | 152,125 | as reference |  |  | as reference |  |  | as reference |  |  | as reference |  |  | as reference |  |  |
|  | Moderate isolated | 134,224 | **1.057(1.010,1.106)** | **1.63E-02** |  | **1.197(1.149,1.247)** | **8.47E-18** |  | **1.250(1.060,1.475)** | **8.04E-03** |  | 1.175(0.997,1.384) | 5.39E-02 |  | 0.909(0.551,1.499) | 7.08E-01 |  |
|  | Most isolated | 46,493 | **1.168(1.100,1.239)** | **3.01E-07** |  | **1.560(1.481,1.642)** | **4.43E-64** |  | **1.504(1.237,1.830)** | **4.43E-05** |  | **1.392(1.141,1.700)** | **1.14E-03** |  | 1.241(0.715,2.153) | 4.43E-01 |  |
| Yes | Least isolated | 43,885 | as reference |  |  | as reference |  |  | as reference |  |  | as reference |  |  | as reference |  |  |
|  | Moderate isolated | 37,976 | **1.118(1.051,1.190)** | **4.31E-04** |  | **1.122(1.087,1.157)** | **8.72E-13** |  | 0.818(0.649,1.031) | 8.95E-02 |  | **1.165(1.033,1.313)** | **1.25E-02** |  | 1.086(0.700,1.686) | 7.11E-01 |  |
|  | Most isolated | 12,891 | **1.164(1.068,1.268)** | **5.18E-04** |  | **1.296(1.242,1.352)** | **3.48E-33** |  | 1.114(0.841,1.474) | 4.52E-01 |  | 1.170(0.993,1.377) | 6.06E-02 |  | 1.310(0.783,2.190) | 3.04E-01 |  |

Abbreviations: Type 2 diabetes mellitus, T2DM; Hazard ratio, HR; Confidence interval, CI; Cardiovascular disease, CVD.

# Supplementary Table 14. Separate and joint effects of loneliness and social isolation on the risk of five transitions after further adjusting for glucose metabolism measures.

|  | *N* | Baseline to incident T2DM | | Baseline to death | | Incident T2DM to subsequent depression | | Incident T2DM to death | | Subsequent depression to death | |
| --- | --- | --- | --- | --- | --- | --- | --- | --- | --- | --- | --- |
|  |  | HR(95%CI) | P value | HR(95%CI) | P value | HR(95%CI) | P value | HR(95%CI) | P value | HR(95%CI) | P value |
| **Separate effects** | |  |  |  |  |  |  |  |  |  |  |
| Loneliness | |  |  |  |  |  |  |  |  |  |  |
| No loneliness | 408,434 | as reference |  | as reference |  | as reference |  | as reference |  | as reference |  |
| Loneliness | 19,160 | **1.124(1.053,1.201)** | **4.90E-04** | **1.169(1.114,1.227)** | **2.76E-10** | **1.800(1.519,2.133)** | **1.12E-11** | 1.026(0.864,1.219) | 7.69E-01 | 1.192(0.797,1.784) | 3.93E-01 |
| Social isolation | |  |  |  |  |  |  |  |  |  |  |
| Least isolated | 196,010 | as reference |  | as reference |  | as reference |  | as reference |  | as reference |  |
| Moderate isolated | 172,200 | **1.053(1.016,1.093)** | **5.26E-03** | **1.150(1.122,1.179)** | **4.21E-28** | 1.089(0.953,1.245) | 2.10E-01 | **1.163(1.056,1.281)** | **2.15E-03** | 0.991(0.714,1.377) | 9.59E-01 |
| Most isolated | 59,384 | **1.117(1.063,1.172)** | **9.22E-06** | **1.401(1.356,1.448)** | **3.88E-91** | **1.367(1.165,1.604)** | **1.28E-04** | **1.271(1.121,1.442)** | **1.88E-04** | 1.260(0.871,1.822) | 2.20E-01 |
| P trend |  |  | **4.98E-06** |  | **1.10E-90** |  | **2.64E-04** |  | **5.59E-05** |  | 2.61E-01 |
| **Joint effects** | |  |  |  |  |  |  |  |  |  |  |
| No loneliness | |  |  |  |  |  |  |  |  |  |  |
| Least isolated | 191,058 | as reference |  | as reference |  | as reference |  | as reference |  | as reference |  |
| Moderate isolated | 163,986 | **1.051(1.013,1.091)** | **9.08E-03** | **1.146(1.117,1.176)** | **9.58E-26** | 1.077(0.935,1.240) | 3.03E-01 | **1.178(1.067,1.301)** | **1.17E-03** | 1.008(0.711,1.427) | 9.66E-01 |
| Most isolated | 53,390 | **1.108(1.052,1.166)** | **1.03E-04** | **1.394(1.347,1.443)** | **6.37E-81** | **1.313(1.101,1.565)** | **2.42E-03** | **1.274(1.115,1.455)** | **3.65E-04** | 1.259(0.838,1.891) | 2.67E-01 |
| Loneliness | |  |  |  |  |  |  |  |  |  |  |
| Least isolated | 4,952 | 1.116(0.974,1.280) | 1.15E-01 | 1.095(0.984,1.219) | 9.59E-02 | **1.979(1.384,2.830)** | **1.83E-04** | 1.141(0.796,1.635) | 4.73E-01 | 1.356(0.579,3.177) | 4.83E-01 |
| Moderate isolated | 8,214 | **1.160(1.049,1.283)** | **3.95E-03** | **1.287(1.195,1.387)** | **3.53E-11** | **1.874(1.433,2.451)** | **4.57E-06** | 1.042(0.791,1.372) | 7.70E-01 | 1.076(0.547,2.117) | 8.31E-01 |
| Most isolated | 5,994 | **1.215(1.089,1.356)** | **4.78E-04** | **1.494(1.382,1.616)** | **5.98E-24** | **2.065(1.573,2.711)** | **1.78E-07** | 1.299(0.986,1.712) | 6.31E-02 | 1.395(0.755,2.578) | 2.88E-01 |
| P trend |  |  | **7.78E-08** |  | **1.27E-72** |  | **1.19E-12** |  | **5.05E-03** |  | 2.07E-01 |

Abbreviations: Type 2 diabetes mellitus, T2DM; Hazard ratio, HR; Confidence interval, CI.

# Supplementary Table 15. Risk of the five transitions according to groups of social isolation in different category of loneliness and groups of loneliness in different category of social isolation after further adjusting for glucose metabolism measures.

|  | *N* | Baseline to incident T2DM | | Baseline to death | | Incident T2DM to subsequent depression | | Incident T2DM to death | | Subsequent depression to death | |
| --- | --- | --- | --- | --- | --- | --- | --- | --- | --- | --- | --- |
|  |  | HR(95%CI) | P value | HR(95%CI) | P value | HR(95%CI) | P value | HR(95%CI) | P value | HR(95%CI) | P value |
| No loneliness | |  |  |  |  |  |  |  |  |  |  |
| Least isolated | 191,058 | as reference |  | as reference |  | as reference |  | as reference |  | as reference |  |
| Moderate isolated | 163,986 | **1.051(1.012,1.091)** | **9.64E-03** | **1.145(1.117,1.175)** | **1.66E-25** | 1.073(0.931,1.236) | 3.29E-01 | **1.180(1.069,1.303)** | **1.04E-03** | 0.987(0.696,1.400) | 9.41E-01 |
| Most isolated | 53,390 | **1.107(1.051,1.165)** | **1.21E-04** | **1.393(1.346,1.441)** | **3.49E-80** | **1.302(1.091,1.553)** | **3.40E-03** | **1.276(1.116,1.457)** | **3.44E-04** | 1.257(0.835,1.891) | 2.73E-01 |
| P trend |  |  | **5.52E-05** |  | **3.16E-80** |  | **5.77E-03** |  | **6.00E-05** |  | 3.38E-01 |
| Loneliness |  |  |  |  |  |  |  |  |  |  |  |
| Least isolated | 4,952 | as reference |  | as reference |  | as reference |  | as reference |  | as reference |  |
| Moderate isolated | 8,214 | 1.045(0.885,1.235) | 6.04E-01 | **1.175(1.033,1.337)** | **1.43E-02** | 0.921(0.602,1.409) | 7.03E-01 | 0.960(0.612,1.506) | 8.58E-01 | 0.375(0.113,1.246) | 1.09E-01 |
| Most isolated | 5,994 | 1.103(0.926,1.315) | 2.72E-01 | **1.364(1.192,1.561)** | **6.41E-06** | 1.033(0.667,1.600) | 8.84E-01 | 1.202(0.753,1.919) | 4.41E-01 | 0.566(0.184,1.746) | 3.22E-01 |
| P trend |  |  | 2.62E-01 |  | **4.41E-06** |  | 7.90E-01 |  | 3.73E-01 |  | 5.53E-01 |
| Least isolated |  |  |  |  |  |  |  |  |  |  |  |
| No loneliness | 191,058 | as reference |  | as reference |  | as reference |  | as reference |  | as reference |  |
| Loneliness | 4,952 | 1.118(0.974,1.282) | 1.12E-01 | 1.102(0.990,1.227) | 7.62E-02 | **1.981(1.382,2.840)** | **1.97E-04** | 1.145(0.798,1.644) | 4.62E-01 | 1.465(0.605,3.550) | 3.98E-01 |
| Moderate isolated |  |  |  |  |  |  |  |  |  |  |  |
| No loneliness | 164,000 | as reference |  | as reference |  | as reference |  | as reference |  | as reference |  |
| Loneliness | 8,214 | 1.105(0.999,1.223) | 5.29E-02 | **1.120(1.039,1.207)** | **2.97E-03** | **1.797(1.376,2.345)** | **1.66E-05** | 0.867(0.659,1.141) | 3.08E-01 | 1.044(0.523,2.083) | 9.02E-01 |
| Most isolated |  |  |  |  |  |  |  |  |  |  |  |
| No loneliness | 53,390 | as reference |  | as reference |  | as reference |  | as reference |  | as reference |  |
| Loneliness | 5,994 | 1.095(0.975,1.229) | 1.24E-01 | 1.043(0.962,1.132) | 3.06E-01 | **1.503(1.126,2.006)** | **5.69E-03** | 1.031(0.771,1.379) | 8.38E-01 | 1.062(0.545,2.070) | 8.61E-01 |
| P interaction |  |  | 0.858 |  | 0.865 |  | 0.407 |  | 0.706 |  | 0.847 |

Abbreviations: Type 2 diabetes mellitus, T2DM; Hazard ratio, HR; Confidence interval, CI.

# Supplementary Table 16. Separate and joint effects of loneliness and social isolation on the risk of five transitions after further adjusting for lipid metabolism measures.

|  | | *N* | Baseline to incident T2DM | | Baseline to death | | Incident T2DM to subsequent depression | | Incident T2DM to death | | Subsequent depression to death | |
| --- | --- | --- | --- | --- | --- | --- | --- | --- | --- | --- | --- | --- |
|  | |  | HR(95%CI) | P value | HR(95%CI) | P value | HR(95%CI) | P value | HR(95%CI) | P value | HR(95%CI) | P value |
| **Separate effects** | | |  |  |  |  |  |  |  |  |  |  |
| Loneliness | |  |  |  |  |  |  |  |  |  |  |  |
| No loneliness | | 408,434 | as reference |  | as reference |  | as reference |  | as reference |  | as reference |  |
| Loneliness | | 19,160 | **1.142(1.069,1.219)** | **7.75E-05** | **1.17(1.115,1.229)** | **2.04E-10** | **1.791(1.511,2.123)** | **1.80E-11** | 1.035(0.871,1.230) | 6.94E-01 | 1.168(0.780,1.748) | 4.51E-01 |
| Social isolation | | |  |  |  |  |  |  |  |  |  |  |
| Least isolated | | 196,010 | as reference |  | as reference |  | as reference |  | as reference |  | as reference |  |
| Moderate isolated | | 172,200 | **1.074(1.036,1.114)** | **1.23E-04** | **1.151(1.123,1.181)** | **1.44E-28** | 1.089(0.953,1.245) | 2.11E-01 | **1.166(1.058,1.284)** | **1.86E-03** | 1.002(0.719,1.395) | 9.93E-01 |
| Most isolated | | 59,384 | **1.160(1.105,1.218)** | **2.19E-09** | **1.405(1.360,1.452)** | **1.01E-92** | **1.369(1.166,1.606)** | **1.19E-04** | **1.267(1.118,1.438)** | **2.24E-04** | 1.296(0.894,1.879) | 1.71E-01 |
| P trend | |  |  | **6.28E-10** |  | **2.96E-92** |  | **2.49E-04** |  | **6.19E-05** |  | 2.05E-01 |
| **Joint effects** | | |  |  |  |  |  |  |  |  |  |  |
| No loneliness | | |  |  |  |  |  |  |  |  |  |  |
| Least isolated | | 191,058 | as reference |  | as reference |  | as reference |  | as reference |  | as reference |  |
| Moderate isolated | | 163,986 | **1.072(1.032,1.113)** | **2.84E-04** | **1.148(1.119,1.177)** | **3.09E-26** | 1.078(0.936,1.242) | 2.96E-01 | **1.181(1.070,1.304)** | **9.85E-04** | 1.008(0.710,1.431) | 9.65E-01 |
| Most isolated | | 53,390 | **1.147(1.090,1.208)** | **1.82E-07** | **1.398(1.351,1.447)** | **2.61E-82** | **1.316(1.103,1.568)** | **2.23E-03** | **1.272(1.114,1.453)** | **3.89E-04** | 1.285(0.855,1.932) | 2.28E-01 |
| Loneliness | |  |  |  |  |  |  |  |  |  |  |  |
| Least isolated | | 4,952 | 1.117(0.974,1.281) | 1.13E-01 | 1.099(0.987,1.223) | 8.47E-02 | **1.989(1.390,2.844)** | **1.66E-04** | 1.173(0.818,1.681) | 3.86E-01 | 1.201(0.509,2.833) | 6.76E-01 |
| Moderate isolated | | 8,214 | **1.185(1.071,1.310)** | **1.01E-03** | **1.287(1.195,1.387)** | **3.57E-11** | **1.852(1.415,2.423)** | **7.09E-06** | 1.055(0.801,1.390) | 7.02E-01 | 1.081(0.548,2.130) | 8.23E-01 |
| Most isolated | | 5,994 | **1.292(1.158,1.441)** | **4.42E-06** | **1.501(1.388,1.623)** | **2.02E-24** | **2.065(1.573,2.712)** | **1.80E-07** | 1.291(0.980,1.702) | 6.94E-02 | 1.418(0.765,2.629) | 2.68E-01 |
| P trend |  |  |  | **1.31E-11** |  | **7.72E-74** |  | **1.61E-12** |  | **4.26E-03** |  | 2.06E-01 |

Abbreviations: Type 2 diabetes mellitus, T2DM; Hazard ratio, HR; Confidence interval, CI.

# Supplementary Table 17. Risk of the five transitions according to groups of social isolation in different category of loneliness and groups of loneliness in different category of social isolation after further adjusting for lipid metabolism measures.

|  | *N* | Baseline to incident T2DM | | Baseline to death | | Incident T2DM to subsequent depression | | Incident T2DM to death | | Subsequent depression to death | |
| --- | --- | --- | --- | --- | --- | --- | --- | --- | --- | --- | --- |
|  |  | HR(95%CI) | P value | HR(95%CI) | P value | HR(95%CI) | P value | HR(95%CI) | P value | HR(95%CI) | P value |
| No loneliness | |  |  |  |  |  |  |  |  |  |  |
| Least isolated | 191,058 | as reference |  | as reference |  | as reference |  | as reference |  | as reference |  |
| Moderate isolated | 163,986 | **1.072(1.032,1.113)** | **3.03E-04** | **1.147(1.118,1.177)** | **5.32E-26** | 1.074(0.933,1.238) | 3.20E-01 | **1.184(1.072,1.308)** | **8.31E-04** | 0.993(0.698,1.412) | 9.67E-01 |
| Most isolated | 53,390 | **1.146(1.089,1.207)** | **2.26E-07** | **1.397(1.350,1.445)** | **1.46E-81** | **1.305(1.094,1.556)** | **3.14E-03** | **1.274(1.115,1.456)** | **3.63E-04** | 1.287(0.854,1.939) | 2.28E-01 |
| P trend |  |  | **4.35E-08** |  | **1.18E-81** |  | **5.34E-03** |  | **5.76E-05** |  | 2.89E-01 |
| Loneliness |  |  |  |  |  |  |  |  |  |  |  |
| Least isolated | 4,952 | as reference |  | as reference |  | as reference |  | as reference |  | as reference |  |
| Moderate isolated | 8,214 | 1.068(0.904,1.262) | 4.38E-01 | **1.167(1.026,1.328)** | **1.91E-02** | 0.932(0.608,1.429) | 7.48E-01 | 0.894(0.568,1.406) | 6.27E-01 | 0.550(0.16,1.898) | 3.44E-01 |
| Most isolated | 5,994 | 1.173(0.984,1.399) | 7.44E-02 | **1.365(1.193,1.562)** | **6.21E-06** | 1.068(0.689,1.655) | 7.69E-01 | 1.152(0.722,1.838) | 5.52E-01 | 0.671(0.18,2.502) | 5.52E-01 |
| P trend |  |  | 6.67E-02 |  | **3.80E-06** |  | 6.76E-01 |  | 4.52E-01 |  | 6.72E-01 |
| Least isolated | |  |  |  |  |  |  |  |  |  |  |
| No loneliness | 191,058 | as reference |  | as reference |  | as reference |  | as reference |  | as reference |  |
| Loneliness | 4,952 | 1.119(0.976,1.284) | 1.08E-01 | 1.104(0.991,1.229) | 7.14E-02 | **1.959(1.365,2.812)** | **2.62E-04** | 1.164(0.810,1.672) | 4.12E-01 | 1.418(0.580,3.465) | 4.44E-01 |
| Moderate isolated | |  |  |  |  |  |  |  |  |  |  |
| No loneliness | 164,000 | as reference |  | as reference |  | as reference |  | as reference |  | as reference |  |
| Loneliness | 8,214 | 1.102(0.996,1.219) | 5.96E-02 | **1.119(1.039,1.206)** | **3.16E-03** | **1.796(1.375,2.346)** | **1.75E-05** | 0.870(0.661,1.146) | 3.22E-01 | 0.976(0.489,1.948) | 9.45E-01 |
| Most isolated | |  |  |  |  |  |  |  |  |  |  |
| No loneliness | 53,390 | as reference |  | as reference |  | as reference |  | as reference |  | as reference |  |
| Loneliness | 5,994 | **1.124(1.002,1.261)** | **4.62E-02** | 1.045(0.963,1.134) | 2.89E-01 | **1.513(1.132,2.021)** | **5.08E-03** | 1.010(0.755,1.351) | 9.48E-01 | 0.978(0.498,1.923) | 9.49E-01 |
| P interaction |  |  | 0.906 |  | 0.857 |  | 0.402 |  | 0.602 |  | 0.999 |

Abbreviations: Type 2 diabetes mellitus, T2DM; Hazard ratio, HR; Confidence interval, CI.

# Supplementary Table 18. Separate effects of loneliness and social isolation on the risk of five transitions after further adjusting for glucose and lipid metabolism measures.

|  | *N* | Baseline to incident T2DM | | Baseline to death | | Incident T2DM to subsequent depression | | Incident T2DM to death | | Subsequent depression to death | |
| --- | --- | --- | --- | --- | --- | --- | --- | --- | --- | --- | --- |
|  |  | HR(95%CI) | P value | HR(95%CI) | P value | HR(95%CI) | P value | HR(95%CI) | P value | HR(95%CI) | P value |
| **Separate effects** |  |  |  |  |  |  |  |  |  |  |  |
| Loneliness |  |  |  |  |  |  |  |  |  |  |  |
| No loneliness | 408,434 | as reference |  | as reference |  | as reference |  | as reference |  | as reference |  |
| Loneliness | 19,160 | **1.105(1.035,1.180)** | **2.88E-03** | **1.170(1.115,1.228)** | **2.18E-10** | **1.796(1.516,2.129)** | **1.41E-11** | 1.037(0.873,1.232) | 6.81E-01 | 1.168(0.780,1.749) | 4.51E-01 |
| Social isolation |  |  |  |  |  |  |  |  |  |  |  |
| Least isolated | 196,010 | as reference |  | as reference |  | as reference |  | as reference |  | as reference |  |
| Moderate isolated | 172,200 | **1.053(1.015,1.092)** | **5.82E-03** | **1.150(1.122,1.180)** | **3.17E-28** | 1.088(0.952,1.244) | 2.18E-01 | **1.164(1.057,1.282)** | **2.06E-03** | 1.012(0.726,1.411) | 9.44E-01 |
| Most isolated | 59,384 | **1.116(1.063,1.172)** | **1.04E-05** | **1.402(1.357,1.449)** | **1.37E-91** | **1.366(1.164,1.602)** | **1.35E-04** | **1.271(1.120,1.441)** | **1.94E-04** | 1.286(0.888,1.864) | 1.83E-01 |
| P trend |  |  | **5.82E-06** |  | **4.03E-91** |  | **2.79E-04** |  | **5.64E-05** |  | 2.15E-01 |
| **Joint effects** |  |  |  |  |  |  |  |  |  |  |  |
| No loneliness |  |  |  |  |  |  |  |  |  |  |  |
| Least isolated | 191,058 | as reference |  | as reference |  | as reference |  | as reference |  | as reference |  |
| Moderate isolated | 163,986 | **1.051(1.012,1.091)** | **9.56E-03** | **1.147(1.118,1.176)** | **6.40E-26** | 1.076(0.934,1.239) | 3.09E-01 | **1.179(1.068,1.302)** | **1.12E-03** | 1.019(0.717,1.448) | 9.15E-01 |
| Most isolated | 53,390 | **1.108(1.052,1.166)** | **1.03E-04** | **1.395(1.349,1.444)** | **2.58E-81** | **1.312(1.100,1.564)** | **2.49E-03** | **1.276(1.117,1.457)** | **3.38E-04** | 1.270(0.844,1.909) | 2.51E-01 |
| Loneliness |  |  |  |  |  |  |  |  |  |  |  |
| Least isolated | 4,952 | 1.091(0.951,1.251) | 2.13E-01 | 1.100(0.988,1.224) | 8.25E-02 | **1.981(1.385,2.833)** | **1.81E-04** | 1.171(0.817,1.679) | 3.90E-01 | 1.197(0.507,2.824) | 6.82E-01 |
| Moderate isolated | 8,214 | **1.139(1.030,1.260)** | **1.14E-02** | **1.286(1.194,1.386)** | **4.09E-11** | **1.863(1.424,2.438)** | **5.77E-06** | 1.058(0.803,1.393) | 6.89E-01 | 1.083(0.549,2.136) | 8.17E-01 |
| Most isolated | 5,994 | **1.201(1.077,1.340)** | **1.03E-03** | **1.498(1.385,1.619)** | **3.51E-24** | **2.064(1.572,2.710)** | **1.84E-07** | 1.294(0.982,1.705) | 6.74E-02 | 1.425(0.769,2.644) | 2.61E-01 |
| P trend |  |  | **5.58E-07** |  | **4.44E-73** |  | **1.50E-12** |  | **3.91E-03** |  | 2.10E-01 |

Abbreviations: Type 2 diabetes mellitus, T2DM; Hazard ratio, HR; Confidence interval, CI.

# Supplementary Table 19. Risk of the five transitions according to groups of social isolation in different category of loneliness and groups of loneliness in different category of social isolation after further adjusting for glucose and lipid metabolism measures.

|  | *N* | Baseline to incident T2DM | | Baseline to death | | Incident T2DM to subsequent depression | | Incident T2DM to death | | Subsequent depression to death | |
| --- | --- | --- | --- | --- | --- | --- | --- | --- | --- | --- | --- |
|  |  | HR(95%CI) | P value | HR(95%CI) | P value | HR(95%CI) | P value | HR(95%CI) | P value | HR(95%CI) | P value |
| No loneliness | |  |  |  |  |  |  |  |  |  |  |
| Least isolated | 191,058 | as reference |  | as reference |  | as reference |  | as reference |  | as reference |  |
| Moderate isolated | 163,986 | **1.050(1.012,1.090)** | **1.05E-02** | **1.146(1.117,1.175)** | **1.12E-25** | 1.073(0.931,1.236) | 3.31E-01 | **1.182(1.071,1.305)** | **9.44E-04** | 1.005(0.706,1.431) | 9.78E-01 |
| Most isolated | 53,390 | **1.106(1.050,1.165)** | **1.30E-04** | **1.394(1.347,1.442)** | **1.52E-80** | **1.301(1.091,1.553)** | **3.44E-03** | **1.278(1.118,1.460)** | **3.12E-04** | 1.278(0.847,1.927) | 2.42E-01 |
| P trend |  |  | **6.13E-05** |  | **1.27E-80** |  | **5.84E-03** |  | **5.30E-05** |  | 2.96E-01 |
| Loneliness |  |  |  |  |  |  |  |  |  |  |  |
| Least isolated | 4,952 | as reference |  | as reference |  | as reference |  | as reference |  | as reference |  |
| Moderate isolated | 8,214 | 1.057(0.894,1.249) | 5.16E-01 | **1.166(1.024,1.327)** | **2.00E-02** | 0.930(0.607,1.426) | 7.40E-01 | 0.898(0.571,1.412) | 6.40E-01 | 0.424(0.124,1.445) | 1.70E-01 |
| Most isolated | 5,994 | 1.120(0.940,1.336) | 2.06E-01 | **1.361(1.189,1.558)** | **7.54E-06** | 1.059(0.683,1.641) | 7.99E-01 | 1.147(0.719,1.831) | 5.65E-01 | 0.760(0.222,2.601) | 6.63E-01 |
| P trend |  |  | 2.00E-01 |  | **4.67E-06** |  | 7.07E-01 |  | 4.68E-01 |  | 8.00E-01 |
| Least isolated |  |  |  |  |  |  |  |  |  |  |  |
| No loneliness | 191,058 | as reference |  | as reference |  | as reference |  | as reference |  | as reference |  |
| Loneliness | 4,952 | 1.092(0.952,1.253) | 2.08E-01 | 1.104(0.992,1.229) | 7.04E-02 | **1.947(1.356,2.794)** | **3.02E-04** | 1.164(0.810,1.673) | 4.11E-01 | 1.420(0.580,3.473) | 4.43E-01 |
| Moderate isolated |  |  |  |  |  |  |  |  |  |  |  |
| No loneliness | 164,000 | as reference |  | as reference |  | as reference |  | as reference |  | as reference |  |
| Loneliness | 8,214 | 1.085(0.980,1.200) | 1.15E-01 | **1.120(1.039,1.207)** | **3.06E-03** | **1.809(1.385,2.364)** | **1.36E-05** | 0.874(0.664,1.150) | 3.36E-01 | 1.014(0.506,2.034) | 9.68E-01 |
| Most isolated |  |  |  |  |  |  |  |  |  |  |  |
| No loneliness | 53,390 | as reference |  | as reference |  | as reference |  | as reference |  | as reference |  |
| Loneliness | 5,994 | 1.083(0.965,1.215) | 1.78E-01 | 1.045(0.963,1.134) | 2.88E-01 | **1.518(1.137,2.027)** | **4.65E-03** | 1.011(0.755,1.352) | 9.43E-01 | 1.023(0.517,2.026) | 9.47E-01 |
| P interaction |  |  | 0.961 |  | 0.847 |  | 0.411 |  | 0.605 |  | 0.976 |

Abbreviations: Type 2 diabetes mellitus, T2DM; Hazard ratio, HR; Confidence interval, CI.

# Supplementary Table 20. Separate and joint effects of loneliness and social isolation on the risk of five transitions after excluding those occurring T2DM within the first year (*n*=426,686, Model 2).

|  | *N* | Baseline to incident T2DM | | Baseline to death | | Incident T2DM to subsequent depression | | Incident T2DM to death | | Subsequent depression to death | |
| --- | --- | --- | --- | --- | --- | --- | --- | --- | --- | --- | --- |
|  |  | HR(95%CI) | P value | HR(95%CI) | P value | HR(95%CI) | P value | HR(95%CI) | P value | HR(95%CI) | P value |
| **Separate effects** | |  |  |  |  |  |  |  |  |  |  |
| Loneliness |  |  |  |  |  |  |  |  |  |  |  |
| No loneliness | 407,587 | as reference |  | as reference |  | as reference |  | as reference |  | as reference |  |
| Loneliness | 19,099 | **1.155(1.081,1.234)** | **2.02E-05** | **1.170(1.114,1.228)** | **3.97E-10** | **1.776(1.495,2.109)** | **6.10E-11** | 1.030(0.865,1.227) | 7.39E-01 | 1.120(0.734,1.709) | 5.99E-01 |
| Social isolation |  |  |  |  |  |  |  |  |  |  |  |
| Least isolated | 195,686 | as reference |  | as reference |  | as reference |  | as reference |  | as reference |  |
| Moderate isolated | 171,804 | **1.077(1.038,1.117)** | **8.14E-05** | **1.147(1.118,1.176)** | **1.80E-26** | 1.084(0.948,1.240) | 2.38E-01 | **1.171(1.062,1.290)** | **1.56E-03** | 0.981(0.705,1.366) | 9.10E-01 |
| Most isolated | 59,196 | **1.167(1.112,1.226)** | **5.83E-10** | **1.397(1.352,1.444)** | **1.57E-87** | **1.357(1.155,1.594)** | **2.03E-04** | **1.272(1.121,1.444)** | **1.98E-04** | 1.244(0.854,1.812) | 2.55E-01 |
| P trend |  |  | **1.66E-10** |  | **8.97E-87** |  | **4.13E-04** |  | **5.24E-05** |  | 3.04E-01 |
| **Joint effects** |  |  |  |  |  |  |  |  |  |  |  |
| No loneliness |  |  |  |  |  |  |  |  |  |  |  |
| Least isolated | 190,747 | as reference |  | as reference |  | as reference |  | as reference |  | as reference |  |
| Moderate isolated | 163,609 | **1.072(1.033,1.114)** | **2.86E-04** | **1.143(1.113,1.172)** | **3.46E-24** | 1.070(0.929,1.234) | 3.47E-01 | **1.186(1.073,1.311)** | **8.09E-04** | 0.986(0.695,1.398) | 9.36E-01 |
| Most isolated | 53,231 | **1.156(1.098,1.218)** | **4.06E-08** | **1.390(1.343,1.439)** | **8.66E-78** | **1.310(1.099,1.563)** | **2.67E-03** | **1.278(1.118,1.461)** | **3.19E-04** | 1.267(0.841,1.908) | 2.58E-01 |
| Loneliness |  |  |  |  |  |  |  |  |  |  |  |
| Least isolated | 4,939 | 1.120(0.975,1.287) | 1.10E-01 | 1.096(0.984,1.222) | 9.49E-02 | **2.007(1.390,2.900)** | **2.05E-04** | 1.181(0.814,1.712) | 3.81E-01 | 1.236(0.491,3.112) | 6.53E-01 |
| Moderate isolated | 8,195 | **1.222(1.104,1.352)** | **1.05E-04** | **1.287(1.194,1.388)** | **5.89E-11** | **1.842(1.408,2.411)** | **8.33E-06** | 1.055(0.801,1.390) | 7.03E-01 | 1.071(0.544,2.107) | 8.43E-01 |
| Most isolated | 5,965 | **1.287(1.153,1.437)** | **7.13E-06** | **1.489(1.375,1.611)** | **6.69E-23** | **2.003(1.517,2.644)** | **9.57E-07** | 1.286(0.971,1.703) | 7.93E-02 | 1.252(0.649,2.416) | 5.02E-01 |
| P trend |  |  | **1.13E-12** |  | **5.83E-70** |  | **8.00E-12** |  | **4.42E-03** |  | 3.34E-01 |

Abbreviations: Type 2 diabetes mellitus, T2DM; Hazard ratio, HR; Confidence interval, CI.

# Supplementary Table 21. Risk of the five transitions according to groups of social isolation in different category of loneliness and groups of loneliness in different category of social isolation after excluding those occurring T2DM within the first year (*n*=426,686, Model 2).

|  | *N* | Baseline to incident T2DM | | Baseline to death | | Incident T2DM to subsequent depression | | Incident T2DM to death | | Subsequent depression to death | |
| --- | --- | --- | --- | --- | --- | --- | --- | --- | --- | --- | --- |
|  |  | HR(95%CI) | P value | HR(95%CI) | P value | HR(95%CI) | P value | HR(95%CI) | P value | HR(95%CI) | P value |
| No loneliness | |  |  |  |  |  |  |  |  |  |  |
| Least isolated | 190,747 | as reference |  | as reference |  | as reference |  | as reference |  | as reference |  |
| Moderate isolated | 163,609 | **1.072(1.032,1.113)** | **3.07E-04** | **1.142(1.113,1.172)** | **5.63E-24** | 1.065(0.924,1.228) | 3.83E-01 | **1.189(1.076,1.314)** | **6.99E-04** | 0.959(0.675,1.362) | 8.14E-01 |
| Most isolated | 53,231 | **1.155(1.097,1.217)** | **5.34E-08** | **1.389(1.342,1.438)** | **3.93E-77** | **1.297(1.087,1.548)** | **3.98E-03** | **1.281(1.121,1.465)** | **2.89E-04** | 1.252(0.830,1.890) | 2.84E-01 |
| P trend |  |  | **1.30E-08** |  | **8.50E-77** |  | **7.11E-03** |  | **4.43E-05** |  | 3.68E-01 |
| Loneliness |  |  |  |  |  |  |  |  |  |  |  |
| Least isolated | 4,939 | as reference |  | as reference |  | as reference |  | as reference |  | as reference |  |
| Moderate isolated | 8,195 | 1.100(0.930,1.302) | 2.66E-01 | **1.174(1.030,1.337)** | **1.60E-02** | 0.895(0.578,1.384) | 6.17E-01 | 0.874(0.552,1.384) | 5.67E-01 | 0.491(0.149,1.619) | 2.42E-01 |
| Most isolated | 5,965 | 1.171(0.981,1.399) | 8.11E-02 | **1.358(1.185,1.556)** | **1.10E-05** | 1.001(0.638,1.572) | 9.95E-01 | 1.053(0.653,1.699) | 8.33E-01 | 0.801(0.249,2.581) | 7.11E-01 |
| P trend |  |  | 8.31E-02 |  | **7.90E-06** |  | 8.79E-01 |  | 7.05E-01 |  | 9.48E-01 |
| Least isolated |  |  |  |  |  |  |  |  |  |  |  |
| No loneliness | 190,747 | as reference |  | as reference |  | as reference |  | as reference |  | as reference |  |
| Loneliness | 4,939 | 1.124(0.977,1.292) | 1.01E-01 | 1.103(0.990,1.229) | 7.59E-02 | **2.002(1.383,2.898)** | **2.36E-04** | 1.197(0.824,1.737) | 3.45E-01 | 1.374(0.529,3.567) | 5.14E-01 |
| Moderate isolated |  |  |  |  |  |  |  |  |  |  |  |
| No loneliness | 163,609 | as reference |  | as reference |  | as reference |  | as reference |  | as reference |  |
| Loneliness | 8,195 | **1.136(1.027,1.258)** | **1.33E-02** | **1.123(1.041,1.211)** | **2.65E-03** | **1.780(1.363,2.325)** | **2.31E-05** | 0.873(0.664,1.150) | 3.34E-01 | 1.052(0.525,2.107) | 8.86E-01 |
| Most isolated |  |  |  |  |  |  |  |  |  |  |  |
| No loneliness | 53,231 | as reference |  | as reference |  | as reference |  | as reference |  | as reference |  |
| Loneliness | 5,965 | 1.112(0.990,1.249) | 7.25E-02 | 1.044(0.961,1.134) | 3.10E-01 | **1.458(1.085,1.958)** | **1.23E-02** | 1.000(0.744,1.345) | 9.98E-01 | 0.885(0.434,1.803) | 7.37E-01 |
| P interaction |  |  | 0.925 |  | 0.847 |  | 0.334 |  | 0.575 |  | 0.803 |

Abbreviations: Type 2 diabetes mellitus, T2DM; Hazard ratio, HR; Confidence interval, CI.

# Supplementary Table 22. Separate and joint association of loneliness and social isolation with the risk of five transitions after further adjusting for neuroticism score.

|  | N | Baseline to incident T2DM | | Baseline to death | | Incident T2DM to subsequent depression | | Incident T2DM to death | | Subsequent depression to death | |
| --- | --- | --- | --- | --- | --- | --- | --- | --- | --- | --- | --- |
|  |  | HR(95%CI) | Pvalue | HR(95%CI) | Pvalue | HR(95%CI) | Pvalue | HR(95%CI) | Pvalue | HR(95%CI) | Pvalue |
| **Separate effects** | |  |  |  |  |  |  |  |  |  |  |
| Loneliness |  |  |  |  |  |  |  |  |  |  |  |
| No loneliness | 408,434 | as reference |  | as reference |  | as reference |  | as reference |  | as reference |  |
| Loneliness | 19,160 | **1.136(1.061,1.215)** | **2.27E-04** | **1.186(1.129,1.247)** | **1.85E-11** | **1.299(1.092,1.546)** | **3.22E-03** | 1.086(0.910,1.296) | 3.59E-01 | 1.294(0.858,1.951) | 2.19E-01 |
| Social isolation | |  |  |  |  |  |  |  |  |  |  |
| Least isolated | 196,010 | as reference |  | as reference |  | as reference |  | as reference |  | as reference |  |
| Moderate isolated | 172,200 | **1.075(1.037,1.115)** | **1.04E-04** | **1.152(1.124,1.181)** | **8.96E-29** | 1.054(0.922,1.204) | 4.43E-01 | **1.171(1.063,1.290)** | **1.34E-03** | 0.986(0.711,1.369) | 9.34E-01 |
| Most isolated | 59,384 | **1.161(1.106,1.219)** | **2.09E-09** | **1.407(1.362,1.454)** | **4.36E-93** | **1.244(1.060,1.460)** | **7.62E-03** | **1.279(1.127,1.451)** | **1.31E-04** | 1.278(0.884,1.850) | 1.92E-01 |
| P trend |  |  | **5.62E-10** |  | **1.27E-92** |  | **1.14E-02** |  | **3.38E-05** |  | 2.34E-01 |
| **Joint effects** | |  |  |  |  |  |  |  |  |  |  |
| No loneliness | |  |  |  |  |  |  |  |  |  |  |
| Least isolated | 191,058 | as reference |  | as reference |  | as reference |  | as reference |  | as reference |  |
| Moderate isolated | 163,986 | **1.073(1.034,1.114)** | **2.16E-04** | **1.148(1.119,1.178)** | **1.99E-26** | 1.053(0.914,1.213) | 4.73E-01 | **1.186(1.074,1.310)** | **7.30E-04** | 1.004(0.710,1.421) | 9.81E-01 |
| Most isolated | 53,390 | **1.152(1.094,1.214)** | **7.37E-08** | **1.400(1.353,1.449)** | **8.95E-83** | **1.235(1.036,1.473)** | **1.87E-02** | **1.275(1.116,1.456)** | **3.44E-04** | 1.260(0.840,1.892) | 2.64E-01 |
| Loneliness |  |  |  |  |  |  |  |  |  |  |  |
| Least isolated | 4,952 | 1.124(0.979,1.29) | 9.70E-02 | **1.119(1.004,1.246)** | **4.16E-02** | 1.396(0.974,2.003) | 6.95E-02 | 1.228(0.854,1.766) | 2.67E-01 | 1.505(0.639,3.544) | 3.50E-01 |
| Moderate isolated | 8,214 | **1.189(1.074,1.318)** | **9.04E-04** | **1.314(1.218,1.417)** | **1.65E-12** | **1.324(1.009,1.737)** | **4.27E-02** | 1.104(0.836,1.457) | 4.87E-01 | 1.131(0.574,2.228) | 7.23E-01 |
| Most isolated | 5,994 | **1.273(1.140,1.422)** | **1.89E-05** | **1.526(1.410,1.651)** | **8.80E-26** | **1.468(1.114,1.933)** | **6.34E-03** | **1.382(1.045,1.826)** | **2.31E-02** | 1.548(0.830,2.887) | 1.69E-01 |
| P trend |  |  | **3.03E-11** |  | **1.70E-77** |  | **2.62E-04** |  | **7.49E-04** |  | 1.13E-01 |

Abbreviations: Type 2 diabetes mellitus, T2DM; Hazard ratio, HR; Confidence interval, CI.

# Supplementary Table 23. Leave‑one‑item sensitivity analysis for the social isolation score assessing associations with the risk of five transitions (Model 2).

|  | *N* | Baseline to incident T2DM | | Baseline to death | | Incident T2DM to subsequent depression | | Incident T2DM to death | | Subsequent depression to death | |
| --- | --- | --- | --- | --- | --- | --- | --- | --- | --- | --- | --- |
|  |  | HR(95%CI) | Pvalue | HR(95%CI) | Pvalue | HR(95%CI) | Pvalue | HR(95%CI) | Pvalue | HR(95%CI) | Pvalue |
| Visit friends & Social activities | | |  |  |  |  |  |  |  |  |  |
| 0 point | 241,547 | as reference |  | as reference |  | as reference |  | as reference |  | as reference |  |
| 1 point | 152,870 | **1.103(1.065,1.143)** | **4.63E-08** | **1.127(1.101,1.155)** | **1.38E-22** | 1.112(0.982,1.259) | 9.43E-02 | 1.080(0.985,1.184) | 1.01E-01 | 1.238(0.922,1.663) | 1.56E-01 |
| 2 points | 33,177 | **1.136(1.068,1.208)** | **4.86E-05** | **1.262(1.209,1.316)** | **4.80E-27** | **1.303(1.069,1.590)** | **8.91E-03** | 1.166(0.993,1.369) | 6.03E-02 | 1.004(0.601,1.676) | 9.89E-01 |
| P trend |  |  | **3.56E-09** |  | **7.98E-39** |  | **6.42E-03** |  | **2.61E-02** |  | 4.57E-01 |
| Visit friends & Living alone | | |  |  |  |  |  |  |  |  |  |
| 0 point | 273,311 | as reference |  | as reference |  | as reference |  | as reference |  | as reference |  |
| 1 point | 139,578 | 1.027(0.991,1.064) | 1.51E-01 | **1.193(1.165,1.222)** | **5.86E-47** | **1.148(1.014,1.298)** | **2.87E-02** | **1.175(1.072,1.288)** | **5.79E-04** | 1.088(0.808,1.466) | 5.77E-01 |
| 2 points | 14,705 | **1.169(1.079,1.267)** | **1.30E-04** | **1.580(1.502,1.661)** | **5.10E-71** | **1.330(1.040,1.700)** | **2.28E-02** | **1.327(1.094,1.611)** | **4.15E-03** | 1.582(0.934,2.679) | 8.83E-02 |
| P trend |  |  | **0.001242** |  | **1.2E-92** |  | **4.32E-03** |  | **4.38E-05** |  | 1.53E-01 |
| Living alone & Social activities | | |  |  |  |  |  |  |  |  |  |
| 0 point | 245,372 | as reference |  | as reference |  | as reference |  | as reference |  | as reference |  |
| 1 point | 160,248 | **1.100(1.063,1.140)** | **8.87E-08** | **1.168(1.141,1.197)** | **5.21E-37** | **1.146(1.011,1.299)** | **3.35E-02** | **1.163(1.061,1.275)** | **1.27E-03** | 0.927(0.682,1.261) | 6.30E-01 |
| 2 points | 21,974 | **1.233(1.154,1.318)** | **5.78E-10** | **1.506(1.443,1.573)** | **6.74E-78** | **1.493(1.227,1.818)** | **6.32E-05** | **1.293(1.097,1.524)** | **2.15E-03** | 1.282(0.829,1.983) | 2.64E-01 |
| P trend |  |  | **4.05E-13** |  | **5.8E-86** |  | **1.03E-04** |  | **9.9E-05** |  | 5.18E-01 |

Abbreviations: Type 2 diabetes mellitus, T2DM; Hazard ratio, HR; Confidence interval, CI.
